# Widespread occurrence of microRNA-mediated target cleavage on membrane-bound polysomes

**DOI:** 10.1186/s13059-020-02242-6

**Published:** 2021-01-05

**Authors:** Xiaoyu Yang, Chenjiang You, Xufeng Wang, Lei Gao, Beixin Mo, Lin Liu, Xuemei Chen

**Affiliations:** 1 Guangdong Provincial Key Laboratory for Plant Epigenetics, Longhua Bioindustry and Innovation Research Institute, College of Life Sciences and Oceanography, Shenzhen University, Shenzhen, 518060 China; 2 Department of Botany and Plant Sciences, Institute of Integrative Genome Biology, University of California, Riverside, CA 92521 USA

**Keywords:** *Zea mays*, *Oryza sativa*, Membrane-bound polysome, miRNA, phasiRNA, siRNA, PARE, Target cleavage

## Abstract

**Background:**

Small RNAs (sRNAs) including microRNAs (miRNAs) and small interfering RNAs (siRNAs) serve as core players in gene silencing at transcriptional and post-transcriptional levels in plants, but their subcellular localization has not yet been well studied, thus limiting our mechanistic understanding of sRNA action.

**Results:**

We investigate the cytoplasmic partitioning of sRNAs and their targets globally in maize (*Zea mays*, inbred line “B73”) and rice (*Oryza sativa*, cv. “Nipponbare”) by high-throughput sequencing of polysome-associated sRNAs and 3′ cleavage fragments, and find that both miRNAs and a subset of 21-nucleotide (nt)/22-nt siRNAs are enriched on membrane-bound polysomes (MBPs) relative to total polysomes (TPs) across different tissues. Most of the siRNAs are generated from transposable elements (TEs), and retrotransposons positively contributed to MBP overaccumulation of 22-nt TE-derived siRNAs (TE-siRNAs) as opposed to DNA transposons. Widespread occurrence of miRNA-mediated target cleavage is observed on MBPs, and a large proportion of these cleavage events are MBP-unique. Reproductive *21PHAS* (*21-nt phasiRNA-generating*) and *24PHAS* (*24-nt phasiRNA-generating*) precursors, which were commonly considered as noncoding RNAs, are bound by polysomes, and high-frequency cleavage of *21PHAS* precursors by miR2118 and *24PHAS* precursors by miR2275 is further detected on MBPs. Reproductive 21-nt phasiRNAs are enriched on MBPs as opposed to TPs, whereas 24-nt phasiRNAs are nearly completely devoid of polysome occupancy.

**Conclusions:**

MBP overaccumulation is a conserved pattern for cytoplasmic partitioning of sRNAs, and endoplasmic reticulum (ER)-bound ribosomes function as an independent regulatory layer for miRNA-induced gene silencing and reproductive phasiRNA biosynthesis in maize and rice.

## Background

RNA silencing via 21–24-nucleotide (nt) small RNAs (sRNAs) is a fundamental mechanism regulating plant gene expression and plays a crucial role in a wide range of plant developmental processes and in responses to a variety of biotic and abiotic stresses [1, 2]. In general, plant sRNAs can be divided into two main categories, microRNAs (miRNAs) and small interfering RNAs (siRNAs). The biosynthesis of plant miRNAs begins with the transcription of miRNA genes (*MIRs*) that are usually located in euchromatic regions of plant chromosomes by RNA polymerase II (Pol II) [1, 2]. The resulting primary miRNAs are processed to hairpin precursor miRNAs (pre-miRNAs) by an RNase III family protein DICER-LIKE 1 (DCL1) [1, 2], which further processes pre-miRNAs into miRNA/miRNA* duplexes [2]. Subsequently, the miRNA/miRNA* duplexes are 2′-O-methylated on the 3′ terminal ribose by the methyltransferase HUA ENHANCER 1 (HEN1) [3]. The miRNA strands are selectively assembled into ARGONAUTE1 (AGO1) to form miRNA-induced silencing complexes (miRISCs) [4, 5]. The miRISCs recognize target genes through base pairing and modulate their expression post-transcriptionally by mRNA cleavage or translation repression in the cytoplasm [1, 2].

In comparison to miRNAs, the origins, biosynthesis, and molecular functions of plant siRNAs are more diverse. Heterochromatic siRNAs are derived from repetitive sequences and transposable elements (TEs). Their biogenesis entails transcription by RNA polymerase IV (Pol IV), double-stranded RNA (dsRNA) formation by RNA-directed RNA polymerase 2 (RDR2), processing of dsRNAs into 24-nt siRNA duplexes by DCL3, methylation of siRNA duplexes by HEN1, and loading of siRNAs into AGO4 [2]. The heterochromatic siRNAs are recruited to target loci by nascent transcripts generated by RNA polymerase V (Pol V) [6] and guide the deposition of repressive chromatin marks such as DNA methylation or histone modifications [7]. Phased secondary siRNAs (phasiRNAs), including trans-acting siRNAs (tasiRNAs), are a second class of siRNAs. tasiRNAs have been extensively studied in *Arabidopsis* and are similar to miRNAs in that they are loaded into AGO1 and repress target genes *in trans* at the post-transcriptional level [2]. The biogenesis of tasiRNAs is triggered by miRNA-guided cleavage of precursor transcripts, which are usually long noncoding transcripts. The cleavage fragments are converted by RDR6 to dsRNAs, which are processed into 21-nt siRNAs by DCL4 [5]. In *Arabidopsis*, *TAS1 (tasiRNA-generating 1)*, *TAS2*, *TAS3*, and *TAS4* loci are targeted for tasiRNA biogenesis by miR173, miR390, and miR828, respectively [8]. In maize and rice, a large number of *PHAS* (*phasiRNA-generating*) loci located in non-repetitive genomic regions generate 21-nt and 24-nt phasiRNAs in anthers [9–11]. There are approximately 460 and 2000 *21PHAS* loci and approximately 170 and 50 *24PHAS* loci in the genomes of maize and rice, respectively [9–11]. The transcripts from *21PHAS* and *24PHAS* loci in maize and rice are cleaved by miR2118- and miR2275-engaged RISCs, respectively [9–11]. The cleavage fragments are further processed by RDR6 and DCL4 to generate 21-nt phasiRNAs or by RDR6 and DCL5 to generate 24-nt phasiRNAs [9–11]. Both 21-nt and 24-nt phasiRNAs act in germline development of maize and rice [12–16]. Reproductive phasiRNAs in plants and piwi-interacting RNAs (piRNAs) in animals share some characteristics, such as presence in the germline, lack of sequence conservation, and being phased [17, 18].

In *Arabidopsis*, the *TAS* transcripts (the precursors to tasiRNAs) contain short open reading frames (ORFs) and are associated with polysomes [19–21]. In fact, isolation of total polysomes (TPs) and membrane-bound polysomes (MBPs) followed by sRNA sequencing revealed the overaccumulation of miRNAs, including those that trigger phasiRNA biogenesis, in the MBP fraction. Indeed, 5′ RACE-PCR detected miRNA-guided cleavage events in the MBP fraction. Sequencing of ribosome-protected fragments in the MBP fraction revealed the presence of *TAS* transcripts on MBPs. These findings suggest that phasiRNA biogenesis is initiated on the rough endoplasmic reticulum (ER) [19]. In contrast, *Arabidopsis* Pol IV-dependent siRNAs, a major component of the 24-nt sRNA population, are not associated with polysomes [19]. Strikingly, a recent study reveals the association of mouse piRNA precursors with ribosomes [22]. The intriguing connection between sRNAs and ribosomes in *Arabidopsis* and mouse prompted us to ask whether such a connection is widespread. We chose to study maize and rice, two model monocots, because they exhibit many differences in sRNA populations from *Arabidopsis*. For example, besides conserved miRNAs, maize and rice have a large number of miRNAs not found in *Arabidopsis*, including miR2118 and miR2275, which serve as triggers for 21-nt and 24-nt phasiRNA biosynthesis in reproductive tissues of diverse monocots and dicots but not in *Arabidopsis* [9–11, 23, 24]. TEs, a source of siRNAs, account for approximately 85% of the maize genome [25] but only approximately 10% of *Arabidopsis* genome [26], suggesting different compositions of sRNAs between the two species. Hundreds or even thousands of *PHAS* loci have been identified in maize and rice [9–11], whereas only a very small number of *PHAS* loci are present in *Arabidopsis* [19]. Furthermore, the observation of the enrichment of miRNAs on MBPs is derived from *Arabidopsis* seedlings, while the cytoplasmic partitioning of sRNAs remains elusive in plant reproductive tissues [19]. These considerations thus promoted us to explore the subcellular compartmentation of sRNAs in monocots across different tissues.

In the present study, we investigated the cytoplasmic partitioning of sRNAs via sequencing of polysome-associated sRNAs in seedling shoots, immature ears, and immature tassels of maize (*Zea mays*, inbred line “B73”), as well as seedling shoots and immature panicles of rice (*Oryza sativa*, cv. “Nipponbare”). MBP overaccumulation was observed for miRNAs and a subset of 21-nt/22-nt siRNAs relative to TP across different tissues. A large proportion of MBP-enriched siRNAs was derived from TEs, in particular retrotransposons as opposed to DNA transposons. We also performed parallel analysis of RNA ends (PARE) to globally detect miRNA-guided cleavage events in input, TP, and MBP samples. Results showed that miRNA-mediated target cleavage for RNA degradation or reproductive phasiRNA biosynthesis widely occurred on MBPs. Distinct cytoplasmic partitioning was observed for reproductive 21-nt and 24-nt phasiRNAs, with 21-nt phasiRNAs but not 24-nt phasiRNAs being enriched on MBPs. PARE analyses detected widespread target cleavage on MBPs mediated by reproductive 21-nt phasiRNAs, thus revealing their functionality in gene regulation. Altogether, our observations uncovered the profound and widespread involvement of ER-bound ribosomes in subcellular accumulation of sRNAs and sRNA-target interactions in plants.

## Results

### Preparation of high-quality polysomal fractions from different tissues of maize and rice

**Fig. 1:**
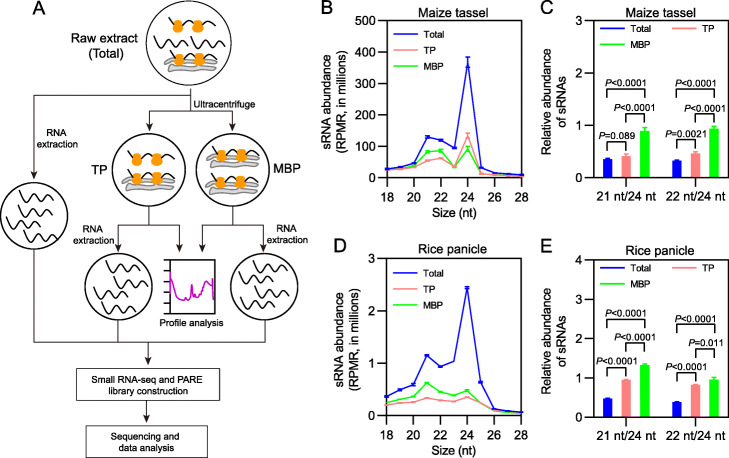
Distinct subcellular partitioning of 21-nt/22-nt and 24-nt sRNAs in maize and rice. **a** Schematic illustration of experimental design for this study. **b, c** Size distribution of sRNAs (**b**) and relative abundance of 21-nt/22-nt and 24-nt sRNAs (**c**) in various fractions from maize immature tassels. **d, e** Size distribution of sRNAs (**d**) and relative abundance of 21-nt/22-nt and 24-nt sRNAs (**e**) in various fractions from rice immature panicles. In **b** and **d**, sRNA abundance is displayed as mean ± standard deviation (SD) of three biological repeats, and “RPMR” is short for “reads per million rRNA fragments”. In **c** and **e**, comparisons between Total, TP, and MBP were performed by two-tailed unpaired *t*-test, and *P* values are displayed above the corresponding comparisons

To begin to understand the cytoplasmic partitioning of sRNAs in monocots, we sought to profile sRNAs from raw extracts (Total), and TP and MBP fractions from various maize and rice tissues (Fig. 1a). Three independent preparations of Total, TP, and MBP from immature ears and immature tassels of maize, and seedling shoots and immature panicles of rice, and two independent preparations of Total, TP, and MBP from maize seedling shoots were generated. The quality of TPs and MBPs was evaluated by both polysome profile analysis and Western and Northern blotting. The integrity and purity of polysomes were well documented in TP and MBP preparations from different tissues of maize and rice (Additional file 1: Fig. S1, S2). These results indicate that high-quality TPs and MBPs were successfully obtained for subsequent library construction.

### Association of small RNAs with MBPs in maize and rice

RNA preparations from Total, TP, and MBP samples were resolved by 15% urea-PAGE and sRNAs ranging from 15 to 40 nt were recovered for library construction. After removing low-quality reads, more than 6 million and 10 million genome-matched sequences for each of the maize and rice libraries, respectively, were obtained, which accounted for approximately 60% and 90% of the sequenced reads (Additional file 2: Table S1). Moreover, the biological repeats for each sample were highly reproducible (Additional file 1: Fig. S3). The relatively large proportion of genome-matched reads and good reproducibility suggested high-quality sRNA libraries in this study.

Normalization of sRNA read counts was performed against rRNAs instead of total reads, because our previous study has shown that rRNA-based normalization is superior when large differences in sRNA composition (miRNA vs. siRNA) are present among samples [19]. After normalization, we initially analyzed the sRNA size distribution in Total, TP, and MBP libraries of maize and rice. The Total sRNA population was characterized by two peak size classes of 24 nt and 21 nt for immature tassels and immature panicles, of 24 nt and 22 nt for maize seedling shoots and immature ears, and of 24 nt and 20 nt for rice seedling shoots (Fig. 1b, d; Additional file 1: Fig. S4A, C, E). These peak sizes are largely consistent with previous findings; variable peak sizes have been observed in previous studies and might depend on plant species, tissue types, developmental stages, environmental conditions, etc. [27–29]. The association of sRNAs with TPs and MBPs was clearly revealed in all maize and rice tissues (Fig. 1b, d; Additional file 1: Fig. S4A, C, E), an observation consistent with previous findings in *Arabidopsis* [19], suggesting that polysome association is a conserved pattern for sRNA cytoplasmic partitioning in plants.

The sRNA populations in TP and MBP samples were dramatically distinct from Total samples. For TP samples from maize and rice, the abundance of the three functional size classes (21 nt, 22 nt, and 24 nt) was all decreased in comparison to Total samples (Fig. 1b, d; Additional file 1: Fig. S4A, C, E). This is similar to the observation from *Arabidopsis* showing lower levels of 21-nt and 24-nt sRNAs in TP as compared to Total [19]. The incorporation of different tissues in this study allowed for comparison to derive trends and differences. Among all tissues, the reduction in sRNA abundance was much greater for the 24-nt size class than that for the other two functional size classes, and as a result, sRNAs in TP displayed an obviously skewed size distribution towards 21 nt and/or 22 nt relative to Total samples (Fig. 1b, d; Additional file 1: Fig. S4A, E). To quantify this trend, we examined the ratios of 21-nt to 24-nt sRNAs and 22-nt to 24-nt sRNAs. These ratios were significantly higher in TP vs. Total for most samples (Fig. 1c, e; Additional file 1: Fig. S4B, F). The only exception was immature ears (Additional file 1: Fig. S4C, D). Thus, it can be concluded that most 24-nt sRNAs are not polysome-associated. This is consistent with observations from *Arabidopsis* [19, 30, 31]. The sRNA profiles of MBP samples were largely similar to those of TP samples, except that the skewing towards higher abundance of 21-nt and 22-nt sRNAs was more pronounced than TP; the 21 nt/24 nt and 22 nt/24 nt ratios were all significantly higher in MBP samples than Total (Fig. 1c, e; Additional file 1: Fig. S4B, D, F). The skewing towards higher abundance of 21-nt sRNAs in the MBP fraction is also observed in *Arabidopsis* [19].

However, we noted one difference between the MBP sRNA profiles from maize and rice and those from *Arabidopsis*. In *Arabidopsis*, the 21-nt peak is much larger for MBP sRNAs than both Total and TP sRNAs, reflecting the enrichment of miRNAs in the MBP fraction [19], but this was not observed in maize and rice. This discrepancy might be attributed to the different proportions of miRNAs in the sRNA populations between *Arabidopsis* and the two grasses used in this study. We categorized the sRNAs in each functional class (21 nt, 22 nt, and 24 nt) based on their genomic origins into the following groups: (I) miRNAs; (II) TE-derived siRNAs (TE-siRNAs), sRNAs mapped to annotated TE loci; (III) gene-derived siRNAs (G-siRNAs), sRNAs mapped to annotated genes; (IV) promoter-derived siRNAs (P-siRNA), sRNAs mapped to promoter regions, which were defined as 1-kb regions upstream of translation start codons of annotated genes in maize and rice genomes; and (V) intergenic region-derived siRNAs (I-siRNAs), sRNAs mapped to genomic regions except *MIR*, TE, gene loci and promoter regions. In MBP samples of both grasses, we found that the majority of 21-nt sRNAs were siRNAs including TE-siRNAs, I-siRNAs, G-siRNAs, and P-siRNAs, while miRNAs only occupied a very small proportion (Additional file 1: Fig. S5); on the contrary, miRNAs are the major component of 21-nt sRNAs in the MBP fraction of *Arabidopsis*, while siRNAs account for a minor proportion [19]. As shown below, although miRNAs were enriched in MBP fractions of maize and rice, their small proportion in the 21-nt class was likely the cause of a less prominent 21-nt peak in the MBP samples.

### Retrotransposon and DNA transposon siRNAs are differentially associated with MBPs in maize

Because of the large contribution of TEs to the maize genome [25] and the sRNA population (Additional file 1: Fig. S5A–C), we characterized siRNA-generating TE loci and the cytoplasmic partitioning of TE-siRNAs in maize. In Total samples, TE-siRNAs consisted of two prominent size classes (22 nt and 24 nt) (Additional file 1: Fig. S6A–C), which were generated from 43,802 and 107,166 TE loci for maize seedling shoots, 81,127 and 152,987 TE loci for immature ears, and 82,890 and 154,256 TE loci for immature tassels, respectively (Additional file 1: Fig. S6K, L). Similar size distribution of TE-siRNAs was also observed in rice (Additional file 1: Fig. S6D, E) and *Arabidopsis* [32]. In comparison to maize seedling shoots, much more TE loci gave rise to siRNAs in immature ears and immature tassels (Additional file 1: Fig. S6K, L). This probably accounted for the differences in sRNA abundance among different maize tissues (Additional file 1: Fig. S6A–C). TE-siRNAs were associated with TPs and MBPs in maize, but abundance of the two predominant size classes (22 nt and 24 nt) was decreased relative to Total samples (Additional file 1: Fig. S6A–C), suggesting that most siRNAs are not polysome-associated. A greater reduction for the 24-nt size class was observed than that for the 22-nt size class of TE-siRNAs (Additional file 1: Fig. S6A–C). As a result, a skewing towards the 22-nt size class was observed for polysome-associated TE-siRNAs, especially MBP-associated TE-siRNAs from immature ears and immature tassels, in comparison to Total samples (Additional file 1: Fig. S6F–H). Similar skewing of TE-siRNAs was observed in rice as well (Additional file 1: Fig. S6I, J).

**Fig. 2:**
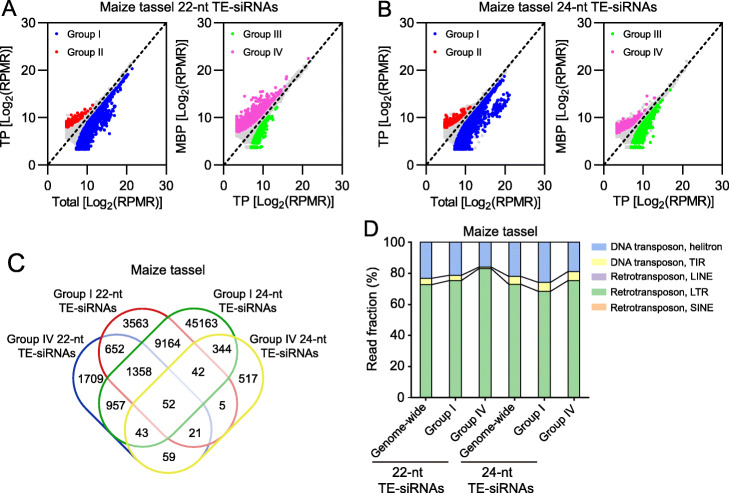
Retrotransposons and DNA transposons contribute differentially to polysome association of TE-derived siRNAs in maize. **a** Identification of 22-nt TE-siRNAs that were differentially accumulated between TP and input (Total) (left panel), and between MBP and TP (right panel) in maize immature tassels. **b** Identification of 24-nt TE-siRNAs that were differentially accumulated between TP and Total (left panel), and between MBP and TP (right panel) in maize immature tassels. TE-siRNA abundance is displayed as the mean of three biological repeats. “RPMR” is short for “reads per million rRNA fragments”. **c** Overlap of TE loci that generated differentially accumulated siRNAs in the various comparisons in **a** and **b**. **d** Contributions of retrotransposons and DNA transposons to the differentially accumulated 22-nt and 24-nt TE-siRNAs in maize immature tassels. “Genome-wide” denotes the contributions of the transposon types to total TE-siRNAs. LINE: long interspersed nuclear element; LTR: long terminal repeat; SINE: short interspersed nuclear element; TIR: terminal inverted repeat. The cutoff parameters for differentially accumulated TE-siRNAs are fold change ≥ 2 and *P* value ≤ 0.05. “Group I,” “Group II,” “Group III,” and “Group IV” represent “TE loci with 22-nt or 24-nt siRNAs that were polysome-depleted,” “TE loci producing 22-nt or 24-nt siRNAs that were polysome-associated,” “TE loci with 22-nt or 24-nt siRNAs that were likely on free polysomes (FPs),” and “TE loci with 22-nt or 24-nt siRNAs enriched on MBPs,” respectively

We next sought to deduce the subcellular distribution of TE-siRNAs by comparing their abundance between TP and Total, and between MBP and TP. We classified TE loci based on fold change of siRNA abundance ≥ 2 and *P* value ≤ 0.05 into four groups: (I) TE loci with siRNAs that were polysome-depleted, (II) TE loci producing siRNAs that were polysome-associated, (III) TE loci with siRNAs that were likely on free polysomes (FPs), and (IV) TE loci with siRNAs enriched on MBPs (Fig. 2a, b; Additional file 1: Fig. S7A, B, S8A, B).

For the 22-nt size class, Group I constituted the largest proportion among the four groups regardless of the tissue type (8958 TE loci for maize seedling shoots, 41,197 for immature ears, and 14,857 for immature tassels) (Fig. 2a; Additional file 1: Fig. S7A, S8A; Additional file 3: Table S2), suggesting that most 22-nt TE-siRNAs are not polysome-associated. Intriguingly, Group IV occupied the second largest proportion, with 466 loci for maize seedling shoots, 12,223 loci for immature ears, and 4851 loci for immature tassels (Fig. 2a; Additional file 1: Fig. S7A, S8A; Additional file 4: Table S3), suggesting that the 22-nt TE-siRNAs, if polysome-associated, are on MBPs. The MBP overaccumulation of 22-nt TE-siRNAs suggested that ER might be a novel regulatory layer for TE-siRNA-mediated PTGS (post-transcriptional gene silencing), which deserves future investigation.

For the 24-nt size class, Group I also constituted an overwhelming majority among the four groups (Fig. 2b; Additional file 1: Fig. S7B, S8B; Additional file 5: Table S4), suggesting that most 24-nt TE-siRNAs are not polysome-associated. This is consistent with the known functions of 24-nt siRNAs in RdDM (RNA-directed DNA methylation) in the nucleus [33, 34]. In fact, most of the Group I loci for 22-nt siRNAs were identical to Group I loci for 24-nt siRNAs (Fig. 2c; Additional file 1: Fig. S7C, S8C), indicating that some of the loci generating 24-nt, non-polysome-associated siRNAs also generated 22-nt siRNAs that were non-polysome-associated. While an overwhelming preference for MBP was observed for the polysome-associated, 22-nt siRNAs, this was not found for polysome-associated, 24-nt siRNAs (compare Fig. 2a, b; Additional file 1: Fig. S8A, B). In fact, most TE loci that produced 22-nt, MBP-associated siRNAs generated 24-nt, non-polysome-associated siRNAs (Fig. 2c; Additional file 1: Fig. S7C, S8C). As 22-nt siRNAs likely function in PTGS, it is possible that the MBP-associated 22-nt siRNAs repress TE RNAs and protein-coding genes through RNA cleavage or translation repression. Consistent with this, the loci that generated 22-nt, MBP-associated siRNAs are enriched for retrotransposons and depleted for DNA transposons (Fig. 2d; Additional file 1: Fig. S7D, S8D). We prepared PARE libraries with the same RNAs used for sRNA libraries with the initial goal of identifying miRNA-guided cleavage. Here, we also took advantage of the PARE libraries to identify potential targets of MBP-associated 22-nt siRNAs in maize. Among the 100 most abundant MBP-associated, 22-nt siRNAs, 6, 11, and 10 were found to target TEs for cleavage in Total, TP, and MBP samples, respectively; and among the 500 most abundant MBP-associated, 22-nt siRNAs, 17, 16, and 22 were found to target protein-coding genes for cleavage in Total, TP, and MBP samples, respectively (Additional file 6: Table S5; Additional file 7: Table S6).

### Association of miRNAs with MBPs displays tissue-to-tissue variations in maize and rice

**Fig. 3:**
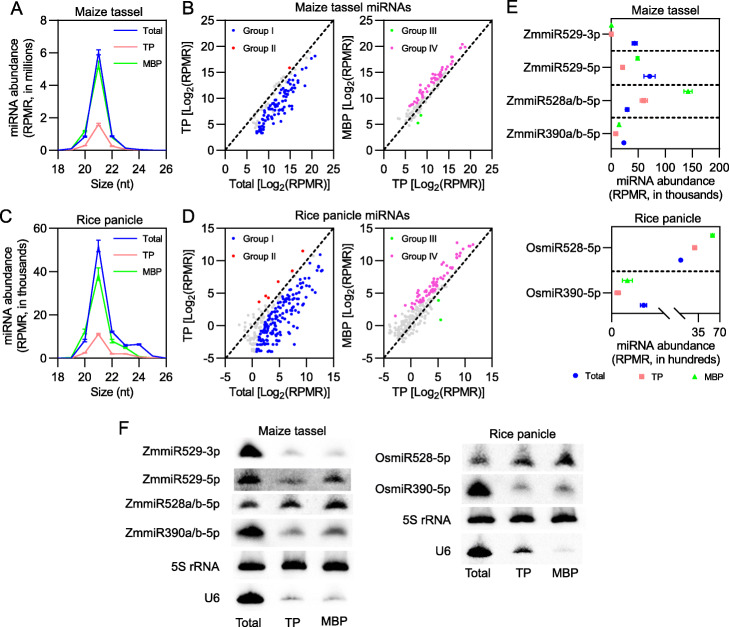
Enrichment of miRNAs on membrane-bound polysomes in maize and rice. **a, c** Size and abundance of all miRNAs in various samples from maize immature tassels (**a**) and rice immature panicles (**c**). **b, d** Identification of differentially accumulated miRNAs between TP and input (Total) (left panels), and between MBP and TP (right panels) in maize immature tassels (**b**) and rice immature panicles (**d**). The cutoff parameters for differentially accumulated miRNAs are fold change ≥ 2 and *P* value ≤ 0.05. “Group I,” “Group II,” “Group III,” and “Group IV” represent “miRNAs that were polysome-depleted,” “miRNAs enriched on polysomes,” “miRNAs that were associated with polysomes but MBP-depleted,” and “miRNAs that were MBP-enriched,” respectively. **e** Abundance of representative miRNAs (ZmmiR390a/b-5p, ZmmiR528a/b-5p, ZmmiR529-5p, ZmmiR529-3p, OsmiR390-5p, and OsmiR528-5p) in Total, TP, and MBP samples from maize immature tassels and rice immature panicles. miRNA abundance is displayed as mean ± standard deviation (SD) (**a**, **c**, and **e**) or the mean of three biological repeats (**b** and **d**). “RPMR” is short for “reads per million rRNA fragments.” **f** Northern blotting verification for miRNAs in (**e**) with Total, TP, and MBP RNA preparations that were used for sRNA library construction. 5S rRNA was used as an internal control, and U6 was used as a nuclear RNA marker

In *Arabidopsis*, miRNAs display a striking MBP enrichment relative to polysomes in general in an AGO1-dependent manner [19]. We sought to test whether miRNAs in maize and rice were also polysome-associated, and if so, whether they were enriched in the MBP fraction. We determined miRNA abundance in Total, TP, and MBP sRNA libraries from the five maize and rice tissues. Indeed, miRNAs were detectable in both TP and MBP fractions, demonstrating the association of miRNAs with polysomes in both grasses (Fig. 3a, c; Additional file 1: Fig. S9A, C, E). In all five tissues, the relative abundance of miRNAs peaked at 21 nt followed by 20-nt and 22-nt size classes regardless of their cellular fractions (Fig. 3a, c; Additional file 1: Fig. S9A, C, E). Comparing the overall abundance of miRNAs among Total, TP, and MBP, we found two commonalities among the five tissues: (1) the abundance of miRNAs in Total samples was constantly higher than that in TP factions and (2) the abundance of miRNAs in MBP fractions was generally higher than that in TP fractions with the only exception being maize shoots (Fig. 3a, c; Additional file 1: Fig. S9A, C, E). Higher overall abundance in the MBP fraction than the TP fraction was also observed for *Arabidopsis* miRNAs [19].

We then deduced the subcellular partitioning of miRNAs by comparing miRNA abundance between TP and Total, and between MBP and TP in the five tissues. Based on fold change ≥ 2 and *P* value ≤ 0.05 in the comparisons, miRNAs were assigned into the following four groups: (I) miRNAs that were polysome-depleted, (II) miRNAs enriched on polysomes, (III) miRNAs that were associated with polysomes but MBP-depleted, and (IV) miRNAs that were MBP-enriched (Fig. 3b, d; Additional file 1: Fig. S9B, D, F). We found that Group I (96 miRNAs for maize seedling shoots, 95 for immature ears, 100 for immature tassels, 111 for rice seedling shoots, and 188 for immature panicles) accounted for the largest proportion among the four groups (Fig. 3b, d; Additional file 1: Fig. S9B, D, F; Additional file 8: Table S7), suggesting that many miRNAs accumulate in the polysome-depleted fraction (perhaps cytosol) in the cell. Group IV (19 for maize seedling shoots, 23 for immature ears, 45 for immature tassels, 53 for rice seedling shoots, and 68 for immature panicles) was the second largest among the four groups (Fig. 3b, d; Additional file 1: Fig. S9B, D, F; Additional file 9: Table S8), further supporting the notion that MBP represents a major subcellular pool for miRNAs in both grasses.

Northern blotting was then applied to specific miRNAs exhibiting particular patterns of subcellular partitioning as determined by sRNA-seq: miR390a/b-5p (polysome-depleted), miR528a/b-5p (MBP-enriched), miR529-5p (polysome-depleted and MBP-enriched), and miR529-3p (polysome-depleted) in immature tassels, and miR390-5p (polysome-depleted) and miR528-5p (MBP-enriched) in immature panicles (Fig. 3e; Additional file 8: Table S7; Additional file 9: Table S8). The levels of these selected miRNAs in Total, TP, and MBP samples as determined by Northern blotting were consistent with results from sRNA-seq (Fig. 3e, f).

### miRNA-mediated target cleavage is widespread on MBPs in maize and rice

**Fig. 4:**
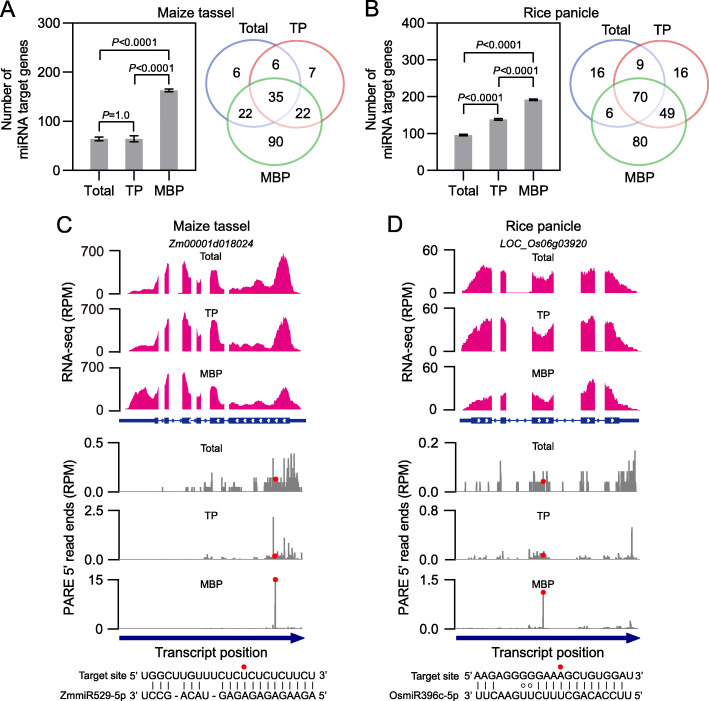
Widespread occurrence of miRNA-mediated target cleavage on membrane-bound polysomes in maize and rice. **a, b** Number (left panels) and overlap of identified miRNA target genes (right panels) in input (Total), TP, and MBP samples from maize immature tassels (**a**) and rice immature panicles (**b**). Cleavage sites that are classified as category 0 with *P* value ≤ 0.05 in at least two biological repeats were filtered as miRNA target sites. Category 0 means that the 3′ cleavage fragments with the maximum read count (> 1) are mapped to only one indicated position on the transcript. In the left panels of (**a**) and (**b**), the number of miRNA target genes is displayed as mean ± standard deviation (SD) of three biological repeats, and comparisons between Total, TP, and MBP were performed by two-tailed unpaired *t*-test and *P* values are displayed above the corresponding comparisons. **c** PolyA RNA-seq read (top panel) and PARE 3′ cleavage fragment (bottom panel) coverage for *Zm00001d018024*, an identified MBP-unique target transcript cleaved by miR529-5p in Total, TP and MBP samples from maize immature tassels. **d** PolyA RNA-seq read (top panel) and PARE 3′ cleavage fragment (bottom panel) coverage for *LOC_Os06g03920*, an identified MBP-unique target transcript cleaved by miR396c-5p, in Total, TP, and MBP samples from rice immature panicles. The gene models are shown below the RNA-seq panels, with the thicker rectangles, lines, and thinner rectangles representing exons, introns, and UTR regions, respectively. In the PARE panels, the red dots indicate the cleavage sites on the transcripts targeted by miRNAs. The sequences of the target sites and the miRNAs are shown below the PARE panels. “PARE” and “RPM” are short for “parallel analysis of RNA ends” and “reads per million mapped reads,” respectively

Plant miRNAs repress target gene expression through either transcript cleavage or translation repression. The polysome association of miRNAs or AGO proteins is usually thought to reflect the translation repression activity of miRNAs. In *Arabidopsis*, miRNA-mediated target cleavage on MBPs has been revealed by 5′ RACE assay for a small number of miRNA targets [19], suggesting that miRNAs can guide the cleavage of target mRNAs that are on polysomes. But it is not clear whether miRNA-guided cleavage of polysome-associated target mRNAs is a widespread phenomenon. Our observation that miRNAs were associated with polysomes and enriched on MBPs prompted us to further explore cleavage events by miRNAs on polysomes, especially MBPs, in maize and rice at the genome-wide level. In vivo, transcriptome-wide detection of 3′ cleavage fragments has been achieved through PARE and similar analyses in a plethora of plants such as *Arabidopsis* [35, 36], maize [9, 11], and rice [11, 37]. In this study, PARE libraries for the five maize and rice tissues were constructed with RNAs from Total, TP, and MBP fractions, the same as those for sRNA library construction (Additional file 2: Table S1). PARE data were processed by the CleaveLand pipeline [38], and 3′ fragments from miRNA-mediated target cleavage were identified by following three criteria: (1) category = 0, which meant that the 3′ cleavage fragments with the maximum read count (> 1) were mapped to only one indicated position on the transcript [38]; (2) *P* value ≤ 0.05; and (3) cleavage was detected in at least two biological repeats. In most cases, more miRNA-guided cleavage events were found in polysomal fractions, and particularly MBP fractions, relative to Total samples (Fig. 4a, b; Additional file 1: Fig. S10A–C; Additional file 10: Table S9), demonstrating the widespread occurrence of miRNA-mediated target cleavage on polysomes in both grasses. We also noted more cleavage events for MBP relative to TP in immature tassels (169 vs. 70) and immature panicles (205 vs. 144), but more cleavage events for TP relative to MBP in maize seedling shoots (86 vs. 60) and immature ears (129 vs. 115) and rice seedling shoots (91 vs. 42) (Fig. 4a, b; Additional file 1: Fig. S10A–C; Additional file 10: Table S9). This shifting between TP and MBP across different tissues might be attributed to variations in the number of miRNAs enriched in the MBP fraction (Additional file 9: Table S8).

The PARE analyses revealed highly compartmentalized miRNA-guided cleavage events at the subcellular level in both grasses. To examine the compartmentalization and explore the relationship between miRNAs and their target genes in each compartment, we first categorized miRNA target genes into the following groups based on the sample in which 3′ cleavage fragments were detected: (I) Total-unique miRNA target genes, for which 3′ cleavage fragments were only detected in the Total sample, suggesting miRNA-guided cleavage in the polysome-depleted fraction; (II) TP-unique miRNA target genes, whose transcripts probably underwent miRNA-guided cleavage on FPs; (III) MBP-unique miRNA target genes, whose transcripts were cleaved by miRNAs only on MBPs; (IV) Total-TP-MBP-common miRNA target genes, whose transcripts were cleaved by miRNAs in polysome-depleted as well as polysomal fractions; (V) Total-TP-common miRNA target genes, whose transcripts were cleaved by miRNAs in membrane-depleted fraction and on FPs; (VI) Total-MBP-common miRNA target genes, whose transcripts were cleaved by miRNAs in polysome-depleted fraction and on MBPs; and (VII) TP-MBP-common miRNA target genes, whose transcripts were cleaved by miRNAs on polysomes (Fig. 4a, b; Additional file 1: Fig. S10A–C). To gain functional insights into the subcellular compartmentalization of cleavage events, Gene Ontology (GO) term analysis was carried out for each group of miRNA target genes and overrepresented terms (*FDR* ≤ 0.05) were observed (Additional file 11: Table S10). For example, MBP-unique target genes in immature tassels were overrepresented in hormone signaling-related GO terms such as “cellular response to auxin stimulus,” “auxin-activated signaling pathway,” and “hormone-mediated signaling pathway,” while TP-MBP-common target genes in immature panicles were associated with regulation-related GO terms such as “regulation of primary metabolic process,” “regulation of cellular biosynthetic process,” and “regulation of cellular metabolic process” (Additional file 11: Table S10).

We focused on the miRNA target genes on MBPs of immature tassels and immature panicles because of the striking overaccumulation of MBP-unique cleavage events relative to other tissues (Fig. 4a, b), and attempted to determine the reasons for MBP-unique cleavage. One possibility is that the target transcripts are only present in the MBP fraction. To this end, polyA RNA-seq was performed for Total, TP, and MBP RNA preparations from immature tassels and immature panicles, the same samples as those for sRNA and PARE library construction, to monitor the transcript levels of MBP-unique target genes. Two MBP-unique targets, *Zm00001d018024* cleaved by miR529-5p in immature tassels and *LOC_Os06g03920* cleaved by miR396c-5p in immature panicles, were selected as representatives for displaying their polyA RNA-seq read and PARE 3′ cleavage fragment coverage, respectively (Fig. 4c, d). Comparable transcript levels were observed in Total, TP, and MBP samples for *Zm00001d018024* and *LOC_Os06g03920*, while the cleavage signals for both were only detected in MBP fractions (Fig. 4c, d). Similar results were also obtained for both MBP-unique and TP-unique targets in maize seedling shoots and immature ears, and rice seedling shoots (Additional file 1: Fig. S10D–I). Then a global-scale expression analysis was done for MBP-unique targets in immature tassels and immature panicles, and no apparent expression bias was observed among Total, TP, and MBP fractions (Additional file 1: Fig. S11A, B), indicating that the MBP-unique cleavage was not due to MBP-enriched accumulation of target transcripts. In contrast, we found that there was a positive relationship between the number of MBP-enriched miRNAs and the number of MBP-unique target genes in both grasses (Additional file 1: Fig. S11C), indicating that the occurrence of MBP-unique cleavage might be at least partially due to the overaccumulation of miRNAs on MBPs.

### Widespread cleavage of reproductive *PHAS* precursors by miR2118 and miR2275 on MBPs in maize and rice

**Fig. 5:**
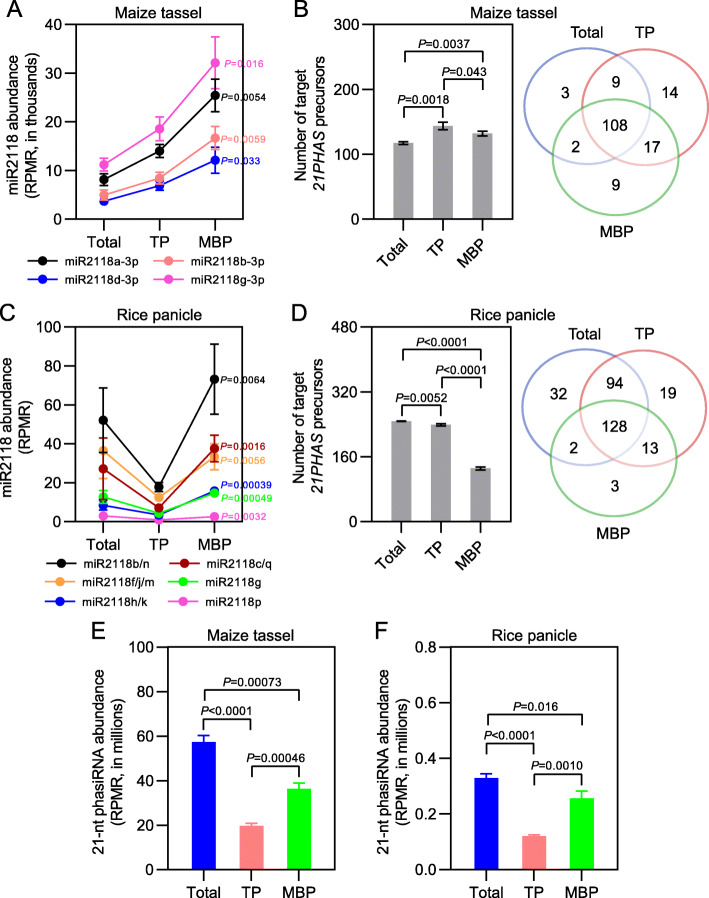
Reproductive *21PHAS* precursors are widely cleaved by miR2118 on membrane-bound polysomes in maize and rice. **a, c** Abundance of different miR2118 members in input (Total), TP, and MBP samples from maize immature tassels (**a**) and rice immature panicles (**c**). **b, d** Number (left panels) and overlap of *21PHAS* precursors cleaved by miR2118 (right panels) in Total, TP, and MBP samples from maize immature tassels (**b**) and rice immature panicles (**d**). Cleavage sites with category = 0, which means that the reads with the maximum count (> 1) are mapped to only one indicated position on the transcript, and *P* value ≤ 0.05 in at least two biological repeats were filtered as miR2118 target sites. **e, f** Abundance of reproductive 21-nt phasiRNAs in Total, TP, and MBP samples from maize immature tassels (**e**) and rice immature panicles (**f**). Abundance of miR2118 and reproductive 21-nt phasiRNAs (**a**, **c**, **e**, and **f**), and number of target *21PHAS* precursors (left panels of **b** and **d**) are displayed as mean ± standard deviation (SD) of three biological repeats. “RPMR” is short for “reads per million rRNA fragments” (**a**, **c**, **e**, and **f**). Comparisons between MBP and TP (**a** and **c**), and between Total, TP, and MBP (left panels of **b** and **d**, **e** and **f**) were performed by two-tailed unpaired *t*-test. *P* values are displayed to the right of the corresponding MBP miR2118s (**a** and **c**) or above the corresponding comparisons (left panels of **b** and **d**, **e** and **f**)

**Fig. 6:**
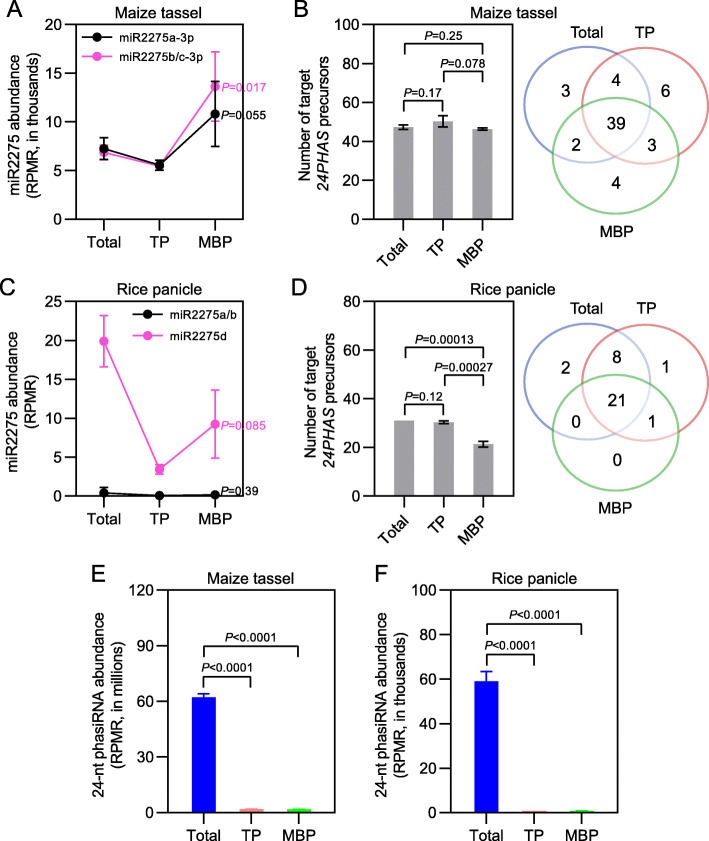
Reproductive *24PHAS* precursors are widely cleaved by miR2275 on membrane-bound polysomes in maize and rice. **a, c** Abundance of different miR2275 members in input (Total), TP, and MBP samples from maize immature tassels (**a**) and rice immature panicles (**c**). **b, d** Number (left panels) and overlap of *24PHAS* precursors cleaved by miR2275 (right panels) in Total, TP, and MBP samples from maize immature tassels (**b**) and rice immature panicles (**d**). Cleavage sites with category = 0, which means that the reads with the maximum count (> 1) are mapped to only one indicated position on the transcript, and *P* value ≤ 0.05 in at least two biological repeats were filtered as miR2275 target sites. **e, f** Abundance of reproductive 24-nt phasiRNAs in Total, TP, and MBP samples from maize immature tassels (**e**) and rice immature panicles (**f**). Abundance of miR2275 and reproductive 24-nt phasiRNAs (**a**, **c**, **e**, and **f**), and the number of target *24PHAS* precursors (left panels of **b** and **d**) are displayed as mean ± standard deviation (SD) of three biological repeats. “RPMR” is short for “reads per million rRNA fragments” (**a**, **c**, **e**, and **f**). Comparisons between MBP and TP (**a** and **c**) and between Total, TP, and MBP (left panels of **b** and **d**, **e** and **f**) were performed by two-tailed unpaired *t*-test, and *P* values are displayed to the right of the corresponding MBP miR2275s (**a** and **c**) or above the corresponding comparisons (left panels of **b** and **d**, **e** and **f**)

In *Arabidopsis*, miRNA-mediated cleavage of *PHAS* precursors occurs on MBPs [19]. We wondered whether *21PHAS* and *24PHAS* precursors, which generated reproductive 21-nt and 24-nt phasiRNAs, respectively, were cleaved on MBPs in immature tassels and immature panicles, two tissues with the most abundant reproductive phasiRNAs (Additional file 1: Fig. S12, S13). To address this question, we first explored subcellular distribution of miR2118 and miR2275, which targeted *21PHAS* and *24PHAS* precursors to trigger phasiRNA biosynthesis [9–11]. For immature tassels, seven miR2118 members (miR2118a-3p–miR2118g-3p) and three miR2275 members (miR2275a-3p–miR2275c-3p) were detectable in Total samples, and all seven miR2118 members and three miR2275 members were observed in TP and MBP fractions; further miR2118 and miR2275 members displayed MBP enrichment to different extents (Figs. 5a and 6a). For immature panicles, eighteen miR2118 members (miR2118a–miR2118r) and four miR2275 members (miR2275a–miR2275d) were present in Total samples; further, the association of miR2118 and miR2275 members with TPs and MBPs was observed, and these miRNAs displayed MBP overaccumulation to different extents relative to TP (Fig. 5c, 6c). Then the expression of *21PHAS* and *24PHAS* loci (Additional file 12: Table S11), reported in previous studies [9–11], was investigated in Total, TP, and MBP samples from immature tassels and immature panicles. The polyA RNA-seq results showed that a large proportion of *21PHAS* and *24PHAS* precursors detected in Total samples were also observed in TP and MBP fractions of immature tassels and immature panicles (Additional file 1: Fig. S14). The polysome association of long noncoding RNAs was observed in previous studies [19–21].

We next sought to determine whether the *21PHAS* and *24PHAS* precursors were cleaved by miR2118 and miR2275, respectively, in the Total, TP, and MBP samples. By analyzing the PARE libraries from immature tassels with the cutoff parameters of category = 0 and *P* value ≤ 0.05 in at least two biological repeats, we observed 122, 148, and 136 miR2118-mediated cleavage events from *21PHAS* precursors in Total, TP, and MBP samples, respectively, and 48, 52, and 48 miR2275-mediated cleavage events from *24PHAS* precursors in Total, TP, and MBP samples, respectively (Figs. 5b and 6b; Additional file 13: Table S12). Similarly for immature panicles, 256, 254, and 146 miR2118-mediated cleavage events were identified for *21PHAS* precursors in Total, TP, and MBP samples, respectively, and 31, 31, and 22 miR2275-mediated cleavage events were identified for *24PHAS* precursors in Total, TP, and MBP samples, respectively (Figs. 5d and 6d; Additional file 13: Table S12). Two *21PHAS* precursors (*Zm21PHAS_NO231* cleaved by miR2118a-3p in immature tassels and *Os21PHAS_NO819* cleaved by miR2118e/r in immature panicles) and two *24PHAS* precursors (*Zm24PHAS_NO236* cleaved by miR2275a-3p in immature tassels and *Os24PHAS_NO5* cleaved by miR2275d in immature panicles) were selected as representatives to display their 3′ cleavage fragment coverage as determined by PARE in Total, TP, and MBP samples (Additional file 1: Fig. S15). Strong cleavage signals for the four selected target *PHAS* precursors were detected in the three samples (Additional file 1: Fig. S15). These data demonstrated widespread miRNA-mediated cleavage of target *PHAS* precursors in Total, TP, and MBP samples in both grasses.

We noted that the number of detected PARE signals was similar in Total, TP, and MBP samples, despite the higher levels of miR2118 or miR2275 in the MBP samples (Figs. 5a–d and 6a–d). This contrasted the much higher number of PARE signals in the MBP samples for miRNAs that do not trigger phasiRNA biogenesis from the same tissues (Fig. 4a, b). We compared the *PHAS* precursor cleavage events identified in Total, TP, and MBP samples from immature tassels and immature panicles. A large degree of overlap in PARE signals was observed among the three samples (Figs. 5b, d and 6b, d), unlike the overwhelming number of MBP-only PARE signals for other miRNAs (Fig. 4a, b). The difference between regular miRNA targets and *PHAS* precursors is that the 3′ cleavage fragments from the latter undergo phasiRNA biogenesis after cleavage. If the subcellular locations of cleavage and subsequent phasiRNA biogenesis steps are different, then the 3′ cleavage fragments would reside at a different location as compared to the site of cleavage. Given that miRNA-mediated cleavage occurs overwhelmingly on MBPs (Fig. 4a, b), we speculate that this is also the case for miR2118 and miR2275, which is supported by the detection of PARE signals in the MBP samples (Figs. 5b, d and 6b, d). We further speculate that the 3′ fragments are released from MBPs upon cleavage to undergo phasiRNA biogenesis in the cytosol; hence, a large number of PARE signals were detected in the Total samples (Figs. 5b, d and 6b, d).

### Reproductive 21-nt and 24-nt phasiRNAs are differentially partitioned between cytosol and polysomes in maize and rice

To gain new insights into the modes of action of reproductive 21-nt and 24-nt phasiRNAs, we explored their abundance in Total, TP, and MBP sRNA libraries from immature tassels and immature panicles. Nearly all reproductive 21-nt phasiRNAs present in Total samples were detected in TP and MBP samples from both tissues (Additional file 14: Table S13), indicating that reproductive 21-nt phasiRNA were associated with polysomes. Comparing the overall abundance of reproductive 21-nt phasiRNAs among Total, TP, and MBP samples, the highest was observed in Total samples, and meanwhile, the overall abundance of reproductive 21-nt phasiRNAs in MBP fractions was significantly higher than that in TP fractions (Fig. 5e, f; Additional file 1: Fig. S16A, B). Such patterns suggested that reproductive 21-nt phasiRNAs were enriched not only in the polysome-depleted fraction but also on MBPs. In contrast, the overall abundance of reproductive 24-nt phasiRNAs in Total samples was overwhelmingly higher than that in TP and MBP fractions (Fig. 6e, f; Additional file 1: Fig. S16A, B), indicating apparent polysome depletion for reproductive 24-nt phasiRNAs.

We then asked which reproductive phasiRNAs displayed polysome depletion or MBP enrichment in immature tassels and immature panicles. To answer this question, we first performed two abundance comparisons for reproductive 21-nt phasiRNAs between TP and Total and between MBP and TP in the two tissues. Reproductive 21-nt phasiRNAs with fold change ≥ 2 and *P* value ≤ 0.05 were sorted into four groups: (I) polysome-depleted 21-nt phasiRNAs, (II) 21-nt phasiRNAs that were polysome-enriched, (III) polysome-associated but MBP-depleted 21-nt phasiRNAs, and (IV) MBP-enriched 21-nt phasiRNAs (Additional file 1: Fig. S16C, E). We found that Group I (388 reproductive 21-nt phasiRNAs for immature tassels and 1877 for immature panicles) was the largest group followed by Group IV (172 for immature tassels and 1574 for immature panicles), with Group II (11 for immature panicles) and Group III (1 for immature panicles) being negligible in numbers (Additional file 1: Fig. S16C, E). Thus, reproductive 21-nt phasiRNAs from both grasses accumulate in either the polysome-depleted or MBP fraction. On the other hand, comparison for reproductive 24-nt phasiRNAs between TP and Total found that nearly all were enriched in polysome-depleted fractions from immature tassels and immature panicles (Additional file 1: Fig. S16D, F). These results demonstrated distinct subcellular partitioning of reproductive 21-nt and 24-nt phasiRNAs in maize and rice.

**Fig. 7:**
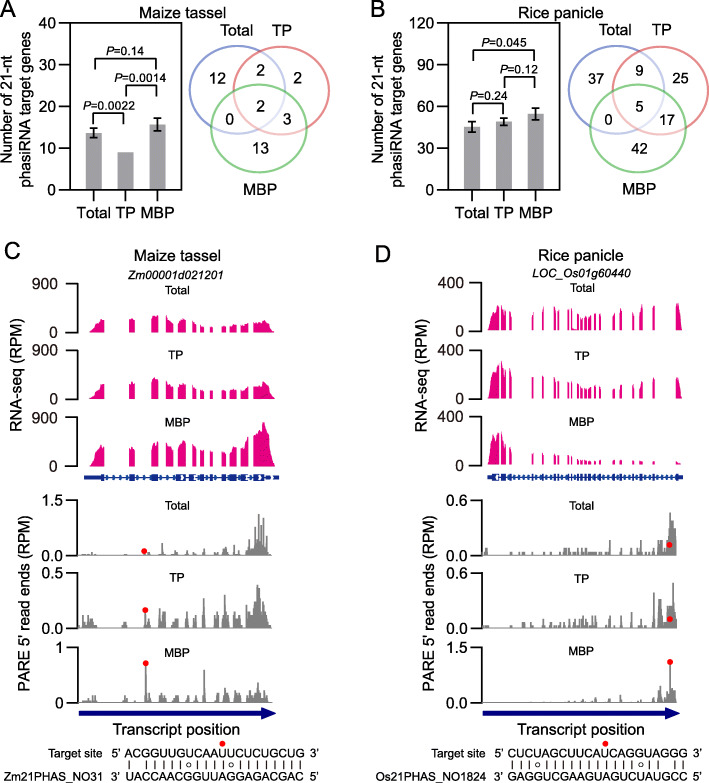
Widespread occurrence of reproductive 21-nt phasiRNA-mediated target cleavage on membrane-bound polysomes in maize and rice. **a, b** Number (left panels) and overlap of identified target genes of reproductive 21-nt phasiRNAs (right panels) in input (Total), TP, and MBP samples from maize immature tassels (**a**) and rice immature panicles (**b**). Cleavage sites with category = 0, which means that the reads with the maximum count (> 1) are mapped to only one indicated position on the transcript, and *P* value ≤ 0.05 in at least two biological repeats were filtered as 21-nt phasiRNA target sites. Number of 21-nt phasiRNA target genes is displayed as mean ± standard deviation (SD) of three biological repeats, and comparisons between Total, TP, and MBP were performed by two-tailed unpaired *t*-test and *P* values are displayed above the corresponding comparisons (left panels of **a** and **b**). **c** PolyA RNA-seq read (top panel) and PARE 3′ cleavage fragment (bottom panel) coverage for *Zm00001d021201*, an identified MBP-unique target transcript cleaved by the 21PHAS_NO31 21-nt phasiRNA, in Total, TP, and MBP samples from maize immature tassels. **d** PolyA RNA-seq read (top panel) and PARE 3′ cleavage fragment (bottom panel) coverage for *LOC_Os01g60440*, an identified MBP-unique target transcript cleaved by the 21PHAS_NO1824 21-nt phasiRNA, in Total, TP, and MBP samples from rice immature panicles. The gene models are shown below the RNA-seq panels, with the thicker rectangles, lines, and thinner rectangles representing exons, introns, and UTR regions, respectively. In the PARE panels, the red dots indicate the cleavage sites on the transcripts targeted by reproductive 21-nt phasiRNAs. The sequences of the target sites and the corresponding phasiRNAs are shown below the PARE panels. “PARE” and “RPM” are short for “parallel analysis of RNA ends” and “reads per million mapped reads,” respectively

Given the observation that many reproductive 21-nt phasiRNAs were associated with polysomes and displayed MBP enrichment, we explored their possible target genes using the PARE data from immature tassels and immature panicles. Sequences of reproductive 21-nt phasiRNAs (read count ≥ 5) were extracted from Total, TP, and MBP sRNA libraries (Additional file 15: Table S14), and then used as queries to search for their target genes (category = 0 and *P* value ≤ 0.05 in at least two biological repeats) in the corresponding PARE libraries with the aid of CleaveLand [38]. The largest number of cleavage events guided by 21-nt phasiRNAs was observed in MBP samples (18 for immature tassels and 64 for immature panicles) followed by Total samples (16 for immature tassels and 51 for immature panicles) and TP samples (9 for immature tassels and 56 for immature panicles) (Fig. 7a, b; Additional file 16: Table S15). This not only demonstrated that reproductive 21-nt phasiRNAs guided target cleavage but also showed widespread target cleavage on MBPs. We characterized the expression profile of these 21-nt phasiRNA target genes by polyA RNA-seq for Total RNA preparations, which were the same as those for sRNA and PARE library construction in the present study, from different tissues of both grasses. In contrast to the strong tissue-specific expression of reproductive 21-nt phasiRNAs (Additional file 1: Fig. S12), most of 21-nt phasiRNA target genes were expressed constitutively across the detected tissues (Additional file 1: Fig. S17).

The relationship of identified 21-nt phasiRNA target genes among Total, TP, and MBP was further investigated. Similar to miRNA target genes, the cleavage events by reproductive 21-nt phasiRNAs were subcellularly compartmentalized into six groups according to the Venn diagrams in Fig. 7a, b, and most target genes were observed in the MBP-unique group. *Zm00001d021201* and *LOC_Os01g60440*, two MBP-unique targets cleaved by maize 21PHAS_NO31 and rice 21PHAS_NO1824 21-nt phasiRNAs respectively, were selected as representatives to show their polyA RNA-seq read and PARE 3′ cleavage fragment coverage (Fig. 7c, d). Similar to MBP-unique miRNA targets, we observed comparable transcript levels for *Zm00001d021201* and *LOC_Os01g60440* in Total, TP, and MBP samples, while the cleavage signals for both were only detected in the MBP fractions (Fig. 7c, d). The overrepresentation of MBP-unique targets was possibly due to the striking overaccumulation of reproductive 21-nt phasiRNAs on MBPs (Additional file 1: Fig. S16C, E). We performed GO term analyses for the six groups of 21-nt phasiRNA target genes to better understand their biological functions, with no significantly enriched GO terms (*FDR* ≤ 0.05) observed (Additional file 17: Table S16), which was consistent with the previous study by Zhai et al. [9]. However, some intriguing target genes associated with plant fertility were observed, possibly providing some new insights into functions of reproductive 21-nt phasiRNAs (Additional file 16: Table S15). For example, maize *Zm00001d035787*, a homolog of the meiotic recombination protein *DMC1*, was cleaved uniquely on MBPs by the 21PHAS_NO105 21-nt phasiRNA. Disturbance of *Arabidopsis DMC1* expression can result in aberrant chromosome behaviors during male and female meiosis, leading to dramatically decreased plant fertility [39, 40]. *SEPALLATA 2* (*Zm00001d021057*) and *SKP1 (LOC_Os07g43230*), another two examples cleaved by 21-nt phasiRNAs of maize 21PHAS_NO248 and rice 21PHAS_NO1658 respectively, have been reported to be involved in reproductive development in *Arabidopsis* [41, 42].

## Discussion

ER is a large membrane-bound organelle in eukaryotic cells and can be divided into smooth and rough ER, depending on polysome association [43]. The biological significance of ER has been well documented in protein biosynthesis, protein folding and trafficking, post-translational modifications of proteins, cellular ion signaling, as well as metabolic processes for cellular carbohydrates, lipids, and sterols [44–49]. ER-related defects can result in severe consequences such as human inflammatory diseases [50] and plant male sterility [51]. Recently, increasing evidence links the rough ER to sRNA-mediated RNA silencing, a conserved regulatory mechanism for gene expression, in eukaryotes, expanding our knowledge of ER’s biological functions. For example, Stalder et al. [52] reported that in human cells siRNAs/miRNAs and their effectors are commonly restricted to the rough ER, whereon RISC assembly and AGO2-mediated cleavage are completed, and TAR RNA binding protein (TRBP) and protein activator of the interferon-induced protein kinase (PACT) play critical roles in associating RISCs with the rough ER. Barman and Bhattacharyya [53] reported that, also in human cells, miRNA-mediated translation repression occurs after target mRNAs are occupied by MBPs, suggesting that translation repression is localized to the rough ER. In *Arabidopsis*, Li et al. [54] reported that miRNA-mediated translation repression of target genes requires ALTERED MERISTEM PROGRAM 1 (AMP1), an integral membrane protein located to the rough ER, and increased levels of miRNA target transcripts were observed on MBPs in *amp1* mutants. Furthermore, a genome-wide analysis of polysome association of sRNAs was conducted in *Arabidopsis* by Li et al. [19] and the results showed that miRNAs are associated with MBPs as opposed to polysomes in general while 24-nt siRNAs are in general non-polysome-associated. Being consistent with the MBP enrichment of miRNAs, the cleavage capacity of membrane-associated miRISCs was confirmed by in vitro slicing assay and 5′ RACE on a small number of miRNA targets [19]. However, the scale of MBP-associated miRNA-guided target cleavage or the generality of MBP-associated miRNA activities across plant species has been unknown.

In this study, we globally investigated the subcellular partitioning of sRNAs in different tissues of maize and rice by profiling sRNAs in total cell extracts versus TP and MBP. A clear, general conclusion is that polysomes are enriched for 21-nt/22-nt sRNAs (both miRNAs and siRNAs) and depleted of 24-nt siRNAs (Fig. 1b–e; Additional file 1: Fig. S4), which is also true for *Arabidopsis* sRNAs [19]. Furthermore, most 21-nt/22-nt miRNAs and some 21-nt/22-nt siRNAs are prone to be enriched on MBPs (Fig. 1b–e; Additional file 1: Fig. S4). siRNAs were the predominant fraction in the sRNA populations associated with TPs and MBPs in both grasses (Additional file 1: Fig. S5), while miRNAs account for the largest proportion of the sRNA populations on TPs and MBPs in *Arabidopsis* [19]. This discrepancy might be mainly due to the different compositions of the sRNA populations between the two grasses (Additional file 1: Fig. S5) and *Arabidopsis* [19], with siRNAs accounting for the majority of 21-nt/22-nt sRNAs in the two grasses but miRNAs accounting for the majority of 21-nt/22-nt sRNAs in *Arabidopsis*. For miRNAs, as in *Arabidopsis* [19], there is a clear trend towards MBP enrichment despite tissue-to-tissue variations in both grasses (Fig. 3b, d; Additional file 1: Fig. S9B, D, F; Additional file 9: Table S8). These results, together with the previous observations in *Arabidopsis*, suggest that MBP enrichment is a conserved pattern for miRNAs and some 21-nt/22-nt siRNAs in plants. However, it is worth noting that the MBP enrichment of miRNAs is relative to other sRNAs such as 24-nt siRNAs or relative to polysomes in general. Many miRNAs also accumulate in polysome-depleted fractions, probably the cytosol (Fig. 3b, d; Additional file 1: Fig. S9B, D, F; Additional file 8: Table S7).

Previous studies that observed the polysome association of miRNAs or ARGONAUTE proteins usually link the association to the translation repression activity of miRNAs [54–56]. In this study, we performed PARE to globally detect the 3′ fragments generated by miRNA-guided RNA cleavage in Total, TP, and MBP samples. To our knowledge, this is the first global examination of miRNA-guided cleavage on polysomes. This analysis, strikingly, revealed not only widespread occurrence of miRNA-mediated target cleavage on MBPs but also an overwhelming number of cleavage events only observed in the MBP fraction (Fig. 4). We then focused on MBP-unique target genes and attempted to find out the reasons why these genes were cleaved uniquely on MBPs. PolyA RNA-seq results showed that transcript levels of MBP-unique target genes were similar in Total and TP as compared to MBP samples (Additional file 1: Fig. S11A, B), indicating that the occurrence of MBP-unique cleavage events is not attributable to the overaccumulation of target mRNAs on the rough ER. Instead, a positive relationship was revealed between the number of miRNAs enriched on MBPs and the number of MBP-unique target genes in both grasses (Additional file 1: Fig. S11C), suggesting that the occurrence of MBP-unique cleavage events might at least partially result from the overaccumulation of miRNAs on MBPs.

MBP enrichment of miRNAs, together with the overwhelming number of MBP-unique target cleavage events, led us to ponder the relationship between the mRNA cleavage and translation repression activities of plant miRNAs. At present, these two activities are thought to be independent. In mutants in genes such as *KATANIN 1 (KTN1)*, *SUO*, and *AMP1*, miRNA target genes are derepressed at the protein but not the transcript level [54–56], suggesting that these genes are only required for the translation repression activity of plant miRNAs. In fact, when examined, miRNA-guided cleavage was found to be still occurring in these mutants [54–56]. However, when considering the subcellular locations, these two activities could be independent or connected. For example, miRNA-guided cleavage may occur to transcripts that are not polysome-associated, in which case the cleavage activity is independent of the translation repression activity. On the other hand, when cleavage occurs on polysomes, it is conceivable that the cleavage activity contributes to translation repression, perhaps by not only eliminating the mRNAs being translated but also preventing the re-initiation of translation of the same mRNAs after a round of translation. The connection between RNA cleavage and translation repression activities of plant miRNAs needs to be explored in the future. Intriguingly, we found that miR156, miR164, miR166, miR172, and miR398, which have been experimentally validated as both cleavage triggers and translation repressors [54, 55, 57–63], were all associated with MBPs, and most of them were enriched on MBPs in the examined tissues of both grasses (Additional file 9: Table S8).

In addition to cleaving target RNAs for degradation, a small portion of miRNAs can serve as triggers to initiate phasiRNA biosynthesis, but the subcellular localization of these events is still largely unknown in plants. Li and co-authors [19] investigated well-known miRNA and phasiRNA precursor pairs such as miR161.1 and *PPR* genes, miR168 and *AGO1*, miR173 and *TAS1*/*TAS2*, miR393 and *AFB2*/*AFB3*, miR472/miR825* and *NBS-LRR* genes, as well as miR828 and *TAS4*, in *Arabidopsis*. They observed that the phasiRNA precursors are MBP-associated and that the generation of phasiRNAs is greatly inhibited in an *ago1* mutant with decreased membrane association of the trigger miRNAs, suggesting that miRNA-mediated cleavage of phasiRNA precursors occurs on MBPs in *Arabidopsis* [19]. In this study, we examined the MBP-phasiRNA connection in maize and rice, which produced numerous phasiRNAs uniquely in reproductive tissues (Additional file 1: Fig. S12, S13). We observed that miR2118 and miR2275, two well-known triggers of phasiRNA biosynthesis cleaving *21PHAS* and *24PHAS* respectively [9–11], were MBP-associated in both immature tassels and immature panicles (Figs. 5a, c and 6a, c). In the two tissues, both miRNAs were MBP-enriched to different extents (Fig. 5a, c and 6a, c). Through PARE, we observed widespread cleavage of *21PHAS* and *24PHAS* transcripts on MBPs by miR2118 and miR2275, respectively (Fig. 5b, d and 6b, d). We also observed a striking difference in the distribution of 3′ cleavage fragments in Total, TP, and MBP fractions from *PHAS* transcripts, as compared to those from other miRNA target transcripts. The latter showed an overwhelming association with MBPs (Fig. 4a, b), while the 3′ cleavage fragments from *PHAS* transcripts were not (Fig. 5b, d and 6b, d). We propose that this difference reflects events that happen after miRNA-guided cleavage. For most targets of miRNAs, presumably, the 3′ cleavage fragments remain polysome-associated until the ribosomes move to the stop codons to be released, which allows for the detection of the 3′ cleavage fragments in the MBP fraction. For *PHAS* transcripts, the lack of MBP enrichment of 3′ cleavage fragments probably reflects their rapid dissociation from polysomes upon cleavage. In fact, in *Arabidopsis*, ribo-seq revealed that ribosomes occupy the portion of *TAS* transcripts 5′ to the miRNA target sites, with the 3′ portion unprotected by ribosomes giving rise to phasiRNAs [19]. Thus, our data are consistent with the biosynthesis of reproductive 21-nt and 24-nt phasiRNAs being initiated on the rough ER in both grasses. After being cleaved, the *21PHAS* and *24PHAS* fragments undergo phasiRNA production at another subcellular location, perhaps associated with membrane vesicles as shown in *Arabidopsis* [64].

We further explored the cytoplasmic partitioning of reproductive 21-nt and 24-nt phasiRNAs in immature tassels and immature panicles. The results showed that reproductive 21-nt phasiRNAs were enriched on MBPs as opposed to polysomes in general, while 24-nt phasiRNAs were restricted to polysome-depleted fractions (Fig. 5e, f and 6e, f; Additional file 1: Fig. S16). The distinct subcellular partitioning hints at different action mechanisms of reproductive phasiRNAs: 21-nt phasiRNAs may regulate gene expression via target cleavage and translation repression like miRNAs, while 24-nt phasiRNAs resemble RdDM 24-nt siRNAs in *Arabidopsis* in terms of their polysome depletion [19] and may regulate target genes at the chromatin level. Altogether, we conclude that ER-bound ribosomes are profoundly involved in subcellular regulation of reproductive phasiRNA biosynthesis as well as action.

Accumulating evidence indicates roles of reproductive phasiRNAs in male fertility in plants. For example, mutation of rice *MEIOSIS ARRESTED AT LEPTOTENE 1* (*MEL1*), which encodes an ARGONAUTE family protein capable of binding reproductive 21-nt phasiRNAs, leads to early meiotic arrest and malfunctional pollen mother cells [12, 13]. *PMS1T*, a rice locus encoding a *21PHAS* transcript, is a modulator of photoperiod-sensitive male sterility in rice [14]. Mutation of *ETERNAL TAPETUM 1* (*EAT1*), encoding a transcription factor activating *24PHAS* and *DCL5* expression, causes microspore abortion in rice [15]. Defects in *DCL5*, which produces 24-nt phasiRNAs, lead to abnormalities of tapetum and temperature-sensitive male sterility in maize [16]. More recently, deletion of the miR2118 cluster in rice chromosome 4, which contains 14 *MIR2118* loci (*MIR2118b*–*MIR2118n*), was demonstrated to cause severe defects in the development and morphology of somatic anther cell walls, and as a result, male and female sterility was observed for these rice mutants, revealing the biological significance of miR2118 and 21-nt phasiRNAs in rice reproduction [65]. However, mechanisms underlying the roles of phasiRNAs in male fertility remain elusive. In this study, we found that each *21PHAS* precursor yielded one predominant 21-nt phasiRNA relative to others (Additional file 15: Table S14), which is consistent with findings from a previous study [11]. PARE signatures consistent with mRNA cleavage guided by abundant 21-nt phasiRNAs were observed, particularly in the MBP fractions (Fig. 7a, b), suggesting that some 21-nt phasiRNAs are functionally similar to miRNAs. Intriguing target genes associated with reproductive development or plant fertility, such as maize *DMC1* (*Zm00001d035787*) and *SEPALLATA 2* (*Zm00001d021057*) as well as rice *SKP1* (*LOC_Os07g43230*) (Additional file 16: Table S15), were found [39–42], possibly providing new insights into functions of reproductive 21-nt phasiRNAs. Intriguingly, moderate expression of reproductive 21-nt and 24-nt phasiRNAs was observed in immature ears as well (Additional file 1: Fig. S12A, S13A), suggesting the potential roles of reproductive phasiRNAs in regulation of female gametogenesis or pistil development. Similar results have been observed in rice [13] and garden asparagus [23].

**Fig. 8:**
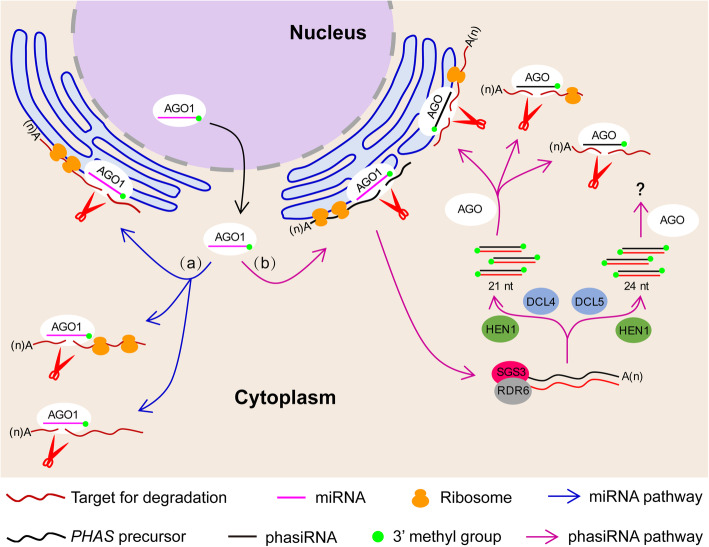
A proposed model of miRNA-mediated target cleavage for RNA degradation or reproductive phasiRNA biosynthesis in plants. miRNA-AGO1 complexes act on target transcripts at different locations in the cytoplasm with various outcomes: (a) miRNAs guide AGO1 proteins to independently cleave target transcripts for degradation on MBPs and FPs, and in the cytosol; (b) miRNAs such as miR2118 or miR2275 can guide AGO1 proteins to cleave reproductive *21PHAS* or *24PHAS* precursors on MBPs, then the phasiRNA-generating cleavage fragments dissociate from MBPs and undergo phasiRNA production through previously identified players such as SGS3, RDR6, HEN1, DCL4, and DCL5. The 21-nt phasiRNAs are loaded into AGO proteins such as rice MEL1 and can cleave target transcripts on MBPs and FPs, and in the cytosol; the 24-nt phasiRNAs are shown to be polysome-depleted in this study but their functions remain unknown. *21PHAS*: *21-nt phasiRNA-generating* locus; *24PHAS*: *24-nt phasiRNA-generating* locus; AGO: ARGONAUTE; DCL: DICER-LIKE; FP: free polysome; HEN1: HUA ENHANCER 1; MBP: membrane-bound polysome; MEL1: MEIOSIS ARRESTED AT LEPTOTENE 1; RDR6: RNA-directed RNA polymerase 6; SGS3: Suppressor of Gene Silencing 3

Integrating our new observations in the present study with previously described results, we propose a new model to explain miRNA-mediated target cleavage, leading to RNA degradation or initiation of reproductive phasiRNA biosynthesis in plants (Fig. 8): Given the presence of miRNAs as well as 3′ cleavage products in non-polysome and polysomal fractions, miRISCs probably guide target RNA cleavage both in the cytosol and on polysomes. On polysomes, miRNAs are enriched on MBPs and cause target RNA cleavage there, but target RNA cleavage on FPs probably also occurs. miR2118 or miR2275 guides the cleavage of *21PHAS* or *24PHAS* transcripts on MBPs, then the phasiRNA-generating cleavage fragments dissociate from polysomes to be processed into reproductive 21-nt phasiRNAs by DCL4 or 24-nt phasiRNAs by DCL5. The 21-nt phasiRNAs are loaded into AGO proteins such as rice MEL1 [13], associate with MBPs, FPs or reside in the cytosol, and guide target mRNA cleavage. The 24-nt phasiRNAs are loaded into AGO proteins such as maize AGO18 [9], are not polysome-associated, and perform as yet unknown functions.

This study also provides new insights into the methodologies for in vivo identification of miRNA target genes in plants. Up to date, PARE, degradome, and GMUCT (genome-wide mapping of uncapped and cleaved transcripts) sequencing have been developed for globally identifying miRNA target genes in vivo [66–68]. Total RNAs isolated from plant tissues of interest are commonly used as starting materials for identification of miRNA target genes via the abovementioned methods [66–68]. In this study, we integrated ultracentrifugation-based isolation of polysomes with PARE to achieve subcellular resolution in monitoring miRNA-mediated cleavage events in different tissues of maize and rice. Intriguingly, a large number of polysome-unique target genes (TP-unique, MBP-unique, and TP-MBP-common) were identified in comparison to those in Total samples of both grasses (Fig. 4a, b; Additional file 1: Fig. S10A–C). This not only uncovers previously unknown, global miRNA-guided cleavage of polysome-bound transcripts, but also provides a new strategy that greatly expands the detection of miRNA target interactions.

## Conclusions

To our knowledge, this is the first report on the subcellular compartmentation of sRNAs in monocots. MBP enrichment of miRNAs was observed across different tissues of maize and rice, suggesting that MBP enrichment is a conserved pattern for miRNAs’ cytoplasmic partitioning. Target transcripts are widely cleaved by miRNAs on MBPs in maize and rice, and particularly a large proportion of cleavage events are MBP-unique, demonstrating that ER-bound ribosomes function as an independent regulatory layer for miRNA-induced gene silencing. Widespread cleavage of reproductive *21PHAS* transcripts by miR2118 and *24PHAS* transcripts by miR2275 were observed on MBPs, and the yielded 21-nt and 24-nt phasiRNAs are distinctly partitioned between cytosol and polysomes, again indicating the profound involvement of ER-bound ribosomes in the biosynthesis and functions of phasiRNAs. These findings provide new insights into modes of action of sRNAs and deepen our understanding of the functions of the rough ER.

## Methods

### Plant materials and growth conditions

Maize (*Zea mays*, inbred line “B73”) and rice (*Oryza sativa*, cv. “Nipponbare”) were used in this study. Seeds were surface-sterilized and then sowed in pots containing nutritional soil in a growth chamber that was set as 16 h light/8 h dark, with air temperature of 28 °C for the light period and 25 °C for the dark period. After germination, seedlings were watered every 5 days and shoots were harvested at the three-leaf stage. The shoots of six seedlings were pooled together as one biological repeat, and totally two and three biological repeats were prepared for maize and rice seedlings, respectively. To harvest immature tassels (3.5 ± 1.5 cm length) and immature ears (3 ± 1 cm length), maize seedlings were grown in the experimental field of Shenzhen University, Guangdong, China, from September to December in 2017. Rice seedlings were grown in the greenhouse of Shenzhen University from January to March in 2018 to harvest immature panicles (3.5 ± 1.5 cm length). Twenty immature tassels and ten immature ears from maize plants, and twenty immature panicles from rice plants were pooled as one biological repeat, respectively, and totally three biological repeats were prepared for each reproductive tissue.

### TP isolation

TP fractions were isolated from different tissues of maize and rice according to a previous method [19] with minor modifications. Briefly, 2 g of plant materials was pulverized with mortar and pestle in liquid nitrogen, transferred to 16 mL polysome extraction buffer (PEB) [0.2 M Tris-HCl (pH 9.0), 0.2 M KCl, 25 mM EGTA, 35 mM MgCl_2_, 1% (W/V) Brij-35, 1% (V/V) Triton X-100, 1% (V/V) Igepal CA630, 1% (V/V) Tween 20, 1% (W/V) polyoxyethylene 10 tridecyl ether, 0.2% (W/V) deoxycholic acid, 5 mM DTT, 1 mM PMSF, 50 μg mL^− 1^ cycloheximide, 50 μg mL^− 1^ chloramphenicol, and 2.5 U mL^− 1^ Invitrogen SuperaseIN], and mixed well by inverting the tube vigorously. The slurry was clarified by centrifugation at 10,000×*g* for 10 min at 4 °C followed by filtering through two layers of miracloth and another centrifugation at 10,000×*g* for 10 min at 4 °C. In total, 500 μL of the supernatant was aliquoted as a “Total” sample. The remainder was then placed on an 8 mL 1.75 M sucrose cushion [0.4 M Tris-HCl (pH 9.0), 0.2 M KCl, 5 mM EGTA, 35 mM MgCl_2_, 5 mM DTT, 50 μg mL^− 1^ cycloheximide, and 50 μg mL^− 1^ chloramphenicol] to collect TPs by centrifugation at 170,000×*g* for 3 h at 4 °C in a Type70 Ti rotor (Beckman Coulter, USA). The resulting TP pellet was resuspended in 400 μL of resuspension buffer [0.2 M Tris-HCl (pH 9.0), 0.2 M KCl, 25 mM EGTA, 35 mM MgCl_2_, 5 mM DTT, 50 μg mL^− 1^ cycloheximide, and 50 μg mL^− 1^ chloramphenicol].

### MBP isolation

MBP fractions were isolated from different tissues of maize and rice according to the method described by Li et al. [19] with minor modifications. Briefly, 4 g of plant materials were pulverized with mortar and pestle in liquid nitrogen. The powder was then transferred to 14 mL microsome extraction buffer (MEB) [0.1 M Tris-HCl (pH 7.5), 5 mM EGTA, 15 mM MgCl_2_, 0.3 M sucrose, 5 mM DTT, 50 μg mL^− 1^ cycloheximide, 50 μg mL^− 1^ chloramphenicol, 2.5 U mL^− 1^ Invitrogen SuperaseIN, and 1 × Roche protease inhibitor cocktail without EDTA] and mixed well by inverting the tube vigorously. The slurry was clarified by a 10-min centrifugation at 10,000×*g* at 4 °C followed by filtering through two layers of miracloth and another 10-min centrifugation at 10,000×*g* at 4 °C. The supernatant was used for subsequent microsome and MBP isolation. To isolate microsomes, the supernatant was placed on a sucrose gradient that was composed of a 2.5 mL of 20% (W/V) sucrose cushion as the top layer and a 2.5 mL of 60% (W/V) sucrose cushion as the bottom layer, and then centrifuged at 100,000×*g* for 1 h at 4 °C in an SW41 rotor (Beckman Coulter, USA). The microsome layer between the 20% and 60% sucrose cushions was transferred to a new tube, diluted with 10 volumes of MEB, and pelleted by centrifugation at 100,000×*g* for 30 min at 4 °C. After 30-min incubation of the resuspended microsomes in 16 mL of PEB on ice, MBPs were isolated by loading the resuspension on an 8 mL 1.75 M sucrose cushion followed by centrifugation at 170,000×*g* for 3 h at 4 °C. The resulting MBP pellet was resuspended in 400 μL of resuspension buffer.

### Polysome profile analysis and RNA extraction

To perform polysome profile analysis, 1000 A260 units of TP or MBP fraction were loaded carefully on a 15–60% (W/V) sucrose gradient that was prepared in a polypropylene centrifuge tube (13 mm × 51 mm, Beckman Coulter, USA) by using a peristaltic pump (BT101S, Lead Fluid, China). The sucrose gradient with TPs or MBPs was then centrifuged in an SW-55 rotor (Beckmann Coulter, USA) at 300,000×*g* for 1.5 h at 4 °C. Fractionation, absorbance assay at 254 nm, and data acquisition of the resulting sample were performed by using a gradient fractionator system (BRANDEL, USA) with a UA-6 absorbance detector (Teledyne ISCO, USA).

RNAs were extracted from Total, TP, and MBP samples of different maize and rice tissues with TRI Reagent following the manufacturer’s instructions (Molecular Research Center, USA), and the concentration of each RNA preparation was determined with a NanoDrop One Microvolume UV-Vis Spectrophotometer (Thermo Fisher, USA).

### sRNA and PARE library construction

To construct sRNA libraries, approximately 20 μg of RNAs from Total, TP, and MBP samples were resolved in a 15% (W/V) TBE-urea polyacrylamide gel, and then the gel slice corresponding to 15-nt–40-nt RNA fragments was collected and smashed. The smashed gel was immersed in 400 μL 0.4 M NaCl solution, agitated at 40 rpm overnight at 4 °C, and spun through a 0.45-μm COSTAR Spin-X filter (Corning, USA) to collect the filtrate. Then sRNAs were recovered from the filtrate by overnight ethanol precipitation [19]. sRNA libraries were finally constructed using the NEBNext Multiplex Small RNA Library Prep Set for Illumina (NEB, USA).

PARE libraries for Total, TP, and MBP samples were constructed according to the method described by Zhai et al. [68]. Briefly, polyA RNAs were isolated from approximately 40 μg of RNA preparation with magnetic beads (Dynabeads, Invitrogen, USA) followed by ligation of 5′ RNA adaptors with the aid of T4 RNA ligase (NEB, USA). After reverse transcription and PCR amplification, double-stranded cDNAs were subjected to magnetic bead purification (Agencourt AMPure XP, Beckman Coulter, USA), *MmeI* (NEB, USA) digestion, and 3′ DNA adaptor ligation. The resulting DNAs were further purified with a 12% (W/V) non-denaturing TBE polyacrylamide gel and recovered by overnight ethanol precipitation [19]. Final PARE libraries were obtained after PCR enrichment and 6% (W/V) non-denaturing TBE polyacrylamide gel purification.

Both sRNA and PARE libraries were sequenced on an Illumina HiSeq2500 platform with the single-end 50-bp sequencing strategy at Berry Genomics Co., Ltd. (Beijing, China).

### RNA-seq library construction

RNA-seq libraries for Total, TP, and MBP samples were constructed with the NEBNext Ultra RNA Library Prep Kit for Illumina (NEB, USA) by following the manufacturer’s instructions. Briefly, polyA RNAs were isolated from approximately 1 μg of RNA preparation with polyT-coated magnetic beads and then treated for divalent cation-mediated fragmentation. After reverse transcription with random primers and second-strand cDNA synthesis, the double-stranded cDNAs were subjected to end repair followed by ligation of hairpin loop adaptors and magnetic bead purification (Agencourt AMPure XP, Beckman Coulter, USA). The yielded product was further treated with USER enzyme (NEB, USA) and enriched by PCR amplification with universal and index primers. Final RNA-seq libraries were recovered with the aid of Agencourt AMPure XP beads (Beckman Coulter, USA), and sequenced on an Illumina HiSeqX10 platform with the pair-end 150-bp sequencing strategy at Novogene Co., Ltd. (Beijing, China).

### Analysis of sRNA-seq

For raw reads from Illumina sRNA-seq datasets, the 3′ adaptor sequence was first trimmed, and then size selection from 18 nt to 42 nt was carried out for adaptor-trimmed reads by using Cutadapt v1.15 [69]. The retained reads were mapped to the AGPv4 genome for “B73” maize (https://www.maizegdb.org/) and MSU Release 7 genome for “Nipponbare” rice (http://rice.plantbiology.msu.edu/index.shtml), respectively, by using ShortStack v3.8.5 [70]. For both genomes, gene and TE annotations were obtained from Gene_model_set Zm00001d.2 for “B73” maize and Gene_model_set Release 7 for “Nipponbare” rice; promoters were manually defined as 1-kb genomic regions upstream of translation start codons of annotated maize and rice genes; and the information about annotated maize and rice miRNAs were adopted from both miRBase v21 (http://www.mirbase.org/) and miRNEST 2.0 (http://rhesus.amu.edu.pl/mirnest/copy/). Adaptor-trimmed reads that were mapped to each gene, TE, promoter, and miRNA were counted respectively and summarized separately for each size class ranging from 18 to 26 nt. Normalization was performed by calculating the RPMR (reads per million rRNA fragments) value for each size class, and comparison was carried out using the R package DESeq2 [71].

### Reproductive phasiRNA analysis

Reproductive phasiRNAs were analyzed according to previously described methods [72, 73]. The number of reproductive 21-nt or 24-nt phasiRNAs was counted by combining sRNAs that were mapped to positive and negative strands of reproductive *21PHAS* or *24PHAS* loci in maize and rice genomes, respectively [9–11]. Phasing score was calculated according to the formula described by Li et al. [19].

### Analysis of PARE data

Raw reads from PARE datasets were initially subjected to adaptor removal using an in-house script. Then the adaptor-trimmed reads were mapped to annotated transcripts of maize or rice to search for miRNA-mediated cleavage events via the CleaveLand4 pipeline [38]. Genes harboring cleavage sites with category = 0 and *P* value ≤ 0.05 in at least two biological repeats were filtered as miRNA targets.

GO term analysis for target genes was performed by using the on-line tool agriGO (http://bioinfo.cau.edu.cn/agriGO/) with default parameters. GO terms with FDR ≤ 0.05 were retained as ones that were statistically significant.

### RNA-seq analysis

Adaptor-trimmed reads from RNA-seq datasets were mapped to the AGPv4 genome for “B73” maize (https://www.maizegdb.org/) or MSU Release 7 genome for “Nipponbare” rice (http://rice.plantbiology.msu.edu/index.shtml) by using Bowtie2 [74], and reads mapped to multiple positions were discarded. Then the number of reads mapped to each gene was counted and used for comparison of normalized gene expression with the R package DESeq2 [71].

### Northern blotting

Northern blotting for select miRNAs was performed as previously described [75]. Briefly, approximately 10 μg of total RNAs were denatured at 70 °C for 10 min and then resolved in a 15% (W/V) TBE-urea polyacrylamide gel by electrophoresis at 150 V for approximately 1 h. The separated RNAs were transferred from the PAGE gel to a nylon membrane (Hybond-NX, GE Healthcare, USA) with the semi-dry method and subjected to 1-ethyl-3-(3-dimethylaminopropyl) carbodiimide hydrochloride (EDC)-mediated crosslinking at 65 °C. For probe preparation, DNA oligonucleotides complementary to targeted miRNAs were synthesized and labeled with biotin molecules at both 5′ and 3′ ends (Additional file 18: Table S17). After hybridization, the probes were detected with the Chemiluminescent Nucleic Acid Detection Module (Thermo Fisher, USA) following the manufacturer’s instructions. Signals were visualized by CheiScope 3300 Mini (CLINX, China). At least two biological repeats were performed for each miRNA.

### Western blotting

Western blotting was performed to investigate ribosomal protein L13 (RPL13) and phosphoenolpyruvate carboxylase (PEPC) levels in polysomal fractions according to the method described by Li et al. [19]. Briefly, approximately 10 μg of total proteins from Total, TP, and MBP samples were subjected to separation in an 8% (W/V) sodium dodecyl sulfate polyacrylamide gel (SDS-PAGE) by electrophoresis at 110 V for about 1 h. After proteins were transferred from the PAGE gel to a nitrocellulose membrane (Amersham Protran, GE Healthcare, USA), RPL13 and PEPC were detected with rabbit anti-RPL13 primary antibody (AS13 2650, Agrisera, USA) and rabbit anti-PEPC primary antibody (AS09 458, Agrisera, USA), respectively. The membranes were then incubated with goat anti-rabbit HRP-conjugated secondary antibody (AS09 602, Agrisera, USA). Blotting signals for RPL13 and PEPC were visualized by incubation with Agrisera ECL SuperBright detection reagent (AS16 ECL-S, Agrisera, USA) followed by exposure to CheiScope 3300 Mini (CLINX, China). Two biological repeats were performed for RPL13 and PEPC, respectively.

## Supplementary Information

**Additional file 1: Figure S1–S17.**

**Additional file 2: Table S1.** Raw data of maize and rice sRNA-seq and PARE libraries.

**Additional file 3: Table S2.** Complete lists of 21-nt and 22-nt differentially expressed transposable element-derived siRNAs between total polysome and input in maize and rice.

**Additional file 4: Table S3.** Complete lists of differentially expressed transposable element-derived siRNAs between membrane-bound polysome and total polysome in maize and rice.

**Additional file 5: Table S4.** Complete lists of 24-nt differentially expressed transposable element-derived siRNAs between total polysome and input in maize and rice.

**Additional file 6: Table S5.** Complete lists of 22-nt transposable element-derived siRNAs that were used for identification of target transcripts in maize.

**Additional file 7: Table S6.** Complete lists of target transcripts of 22-nt transposable element-derived siRNAs based on the maize PARE data.

**Additional file 8: Table S7.** Complete lists of differentially expressed miRNAs between total polysome and input in maize and rice.

**Additional file 9: Table S8.** Complete lists of differentially expressed miRNAs between membrane-bound polysome and total polysome in maize and rice.

**Additional file 10: Table S9.** Complete lists of miRNA target genes in maize and rice.

**Additional file 11: Table S10.** Complete lists of Gene Ontology terms for miRNA target genes in maize and rice.

**Additional file 12: Table S11.** Locations of reproductive *PHAS* loci in maize and rice genomes.

**Additional file 13: Table S12.** Complete lists of reproductive *PHAS* precursors that were cleaved by miR1859, miR2118 and miR2275 in maize immature tassels and rice immature panicles.

**Additional file 14: Table S13.** Abundance of reproductive phasiRNAs in maize and rice.

**Additional file 15: Table S14.** Complete lists of reproductive 21-nt phasiRNAs that were used for identification of target genes in maize immature tassels and rice immature panicles.

**Additional file 16: Table S15.** Complete lists of target genes of reproductive 21-nt phasiRNAs based on the PARE data from maize immature tassels and rice immature panicles.

**Additional file 17: Table S16.** Complete lists of Gene Ontology terms for target genes of reproductive 21-nt phasiRNAs in maize immature tassels and rice immature panicles.

**Additional file 18: Table S17.** DNA probes used for detection of miRNAs in maize and rice by Northern blotting.

**Additional file 19.** Review history.

## Data Availability

The data reported in this paper have been deposited in the NCBI Sequence Read Archive (SRA) database (https://submit.ncbi.nlm.nih.gov/subs/sra/) under the accession no. PRJNA635281 [76].

## References

[1] Song X, Li Y, Cao X, Qi Y (2019). MicroRNAs and their regulatory roles in plant-environment interactions. Annu Rev Plant Biol.

[2] Yu Y, Zhang Y, Chen X, Chen Y (2019). Plant noncoding RNAs: hidden players in development and stress responses. Annu Rev Cell Dev Bi.

[3] Yu B, Yang Z, Li J, Minakhina S, Yang M, Padgett RW (2005). Methylation as a crucial step in plant microRNA biogenesis. Science..

[4] Bologna NG, Voinnet O (2014). The diversity, biogenesis, and activities of endogenous silencing small RNAs in *Arabidopsis*. Annu Rev Plant Biol.

[5] Yu Y, Jia T, Chen X (2017). The ‘how’ and ‘where’ of plant microRNAs. New Phytol.

[6] Wang F, Axtell MJ (2017). AGO4 is specifically required for heterochromatic siRNA accumulation at Pol V-dependent loci in *Arabidopsis thaliana*. Plant J.

[7] Liu L, Chen X (2016). RNA quality control as a key to suppressing RNA silencing of endogenous genes in plants. Mol Plant.

[8] De Felippes FF, Marchais A, Sarazin A, Oberlin S, Voinnet O (2017). A single miR390 targeting event is sufficient for triggering TAS3-tasiRNA biogenesis in *Arabidopsis*. Nucleic Acids Res.

[9] Zhai J, Zhang H, Arikit S, Huang K, Nan GL, Walbot V (2015). Spatiotemporally dynamic, cell-type-dependent premeiotic and meiotic phasiRNAs in maize anthers. PNAS..

[10] Fei Q, Yang L, Liang W, Zhang D, Meyers BC (2016). Dynamic changes of small RNAs in rice spikelet development reveal specialized reproductive phasiRNA pathways. J Exp Bot.

[11] Tamim S, Cai Z, Mathioni SM, Zhai J, Teng C, Zhang Q (2018). *Cis*-directed cleavage and nonstoichiometric abundances of 21-nucleotide reproductive phased small interfering RNAs in grasses. New Phytol.

[12] Nonomura KI, Morohoshi A, Nakano M, Eiguchi M, Miyao A, Hirochika H (2007). A germ cell-specific gene of the ARGONAUTE family is essential for the progression of premeiotic mitosis and meiosis during sporogenesis in rice. Plant Cell.

[13] Komiya R, Ohyanagi H, Niihama M, Watanabe T, Nakano M, Kurata N (2014). Rice germline-specific Argonaute MEL1 protein binds to phasiRNAs generated from more than 700 lincRNAs. Plant J.

[14] Fan Y, Yang J, Mathioni SM, Yu J, Shen J, Yang X (2016). *PMS1T*, producing phased small-interfering RNAs, regulates photoperiod-sensitive male sterility in rice. PNAS..

[15] Ono S, Liu H, Tsuda K, Fukai E, Tanaka K, Sasaki T (2018). EAT1 transcription factor, a non-cell-autonomous regulator of pollen production, activates meiotic small RNA biogenesis in rice anther tapetum. PLoS Genet.

[16] Teng C, Zhang H, Hammond R, Huang K, Meyers BC, Walbot V. *Dicer-like 5* deficiency confers temperature-sensitive male sterility in maize. bioRxiv. 2018; 10.1101/498410. 10.1038/s41467-020-16634-6 PMC7283321 32518237

[17] Han BW, Wang W, Li C, Weng Z, Zamore PD (2015). piRNA-guided transposon cleavage initiates Zucchini-dependent, phased piRNA production. Science.

[18] Mohn F, Handler D, Brennecke J (2015). piRNA-guided slicing specifies transcripts for Zucchini-dependent, phased piRNA biogenesis. Science.

[19] Li S, Le B, Ma X, Li S, You C, Yu Y (2016). Biogenesis of phased siRNAs on membrane-bound polysomes in *Arabidopsis*. eLife..

[20] Bazin J, Baerenfaller K, Gosai SJ, Gregory BD, Crespi M, Bailey-Serres J (2017). Global analysis of ribosome-associated noncoding RNAs unveils new modes of translational regulation. PNAS..

[21] Traubenik S, Reynoso MA, Hobecker K, Lancia M, Hummel M, Rosen B (2020). Reprogramming of root cells during nitrogen-fixing symbiosis involves dynamic polysome association of coding and noncoding RNAs. Plant Cell.

[22] Sun YH, Zhu J, Xie LH, Li Z, Meduri R, Zhu X (2020). Ribosomes guide pachytene piRNA formation on long intergenic piRNA precursors. Nat Cell Biol.

[23] Kakrana A, Mathioni SM, Huang K, Hammond R, Vandivier L, Patel P (2018). Plant 24-nt reproductive phasiRNAs from intramolecular duplex mRNAs in diverse monocots. Genome Res.

[24] Xia R, Chen C, Pokhrel S, Ma W, Huang K, Patel P (2019). 24-nt reproductive phasiRNAs are broadly present in angiosperms. Nat Commun.

[25] Schnable PS, Ware D, Fulton RS, Stein JC, Wei F, Pasternak S (2009). The B73 maize genome: complexity, diversity, and dynamics. Science..

[26] The Arabidopsis Genome Initiative (2000). Analysis of the genome sequence of the flowering plant *Arabidopsis thaliana*. Nature..

[27] Jeong DH, Park S, Zhai J, Gurazada SGR, De Paoli E, Meyers BC (2011). Massive analysis of rice small RNAs: mechanistic implications of regulated microRNAs and variants for differential target RNA cleavage. Plant Cell.

[28] Lunardon A, Forestan C, Farinati S, Axtell MJ, Varotto S (2016). Genome-wide characterization of maize small RNA loci and their regulation in the *required to maintain repression 6-1*(*rmr6-1*) mutant and long-term abiotic stresses. Plant Physiol.

[29] He J, Jiang Z, Gao L, You C, Ma X, Wang X (2019). Genome-wide transcript and small RNA profiling reveals transcriptomic responses to heat stress. Plant Physiol.

[30] Dalmadi Á, Gyula P, Bálint J, Szittya G, Havelda Z (2019). AGO-unbound cytosolic pool of mature miRNAs in plant cells reveals a novel regulatory step at AGO1 loading. Nucleic Acids Res.

[31] Marchais A, Chevalier C, Voinnet O (2019). Extensive profiling in *Arabidopsis* reveals abundant polysome-associated 24-nt small RNAs including AGO5-dependent pseudogene-derived siRNAs. RNA..

[32] Borges F, Parent JS, Van Ex F, Wolff P, Martínez G, Köhler G (2018). Transposon-derived small RNAs triggered by miR845 mediate genome dosage response in *Arabidopsis*. Nat Genet.

[33] Wei L, Gu L, Song X, Cui X, Lu Z, Zhou M (2014). Dicer-like 3 produces transposable element-associated 24-nt siRNAs that control agricultural traits in rice. PNAS..

[34] Zhang H, Tao Z, Hong H, Chen Z, Wu C, Li X (2016). Transposon-derived small RNA is responsible for modified function of *WRKY45* locus. Nat Plants.

[35] Crisp PA, Ganguly DR, Smith AB, Murray KD, Estavillo GM, Searle I (2017). Rapid recovery gene downregulation during excess-light stress and recovery in *Arabidopsis*. Plant Cell.

[36] Nagarajan VK, Kukulich PM, Von Hagel B, Green PJ (2019). RNA degradomes reveal substrates and importance for dark and nitrogen stress responses of *Arabidopsis XRN4*. Nucleic Acids Res.

[37] Chung PJ, Jung H, Jeong D, Ha SH, Choi YD, Kim JK (2016). Transcriptome profiling of drought responsive noncoding RNAs and their target genes in rice. BMC Genomics.

[38] Addo-Quaye C, Miller W, Axtell MJ (2009). CleaveLand: a pipeline for using degradome data to find cleaved small RNA targets. Bioinformatics..

[39] Couteau F, Belzile F, Horlow C, Grandjean O, Vezon D, Doutriaux MP (1999). Random chromosome segregation without meiotic arrest in both male and female meiocytes of a *dmc1* mutant of *Arabidopsis*. Plant Cell.

[40] Da Ines O, Degroote F, Goubely C, Amiard S, Gallego ME, Charles I (2013). White meiotic recombination in *Arabidopsis* is catalysed by DMC1, with RAD51 playing a supporting role. Plos Genet.

[41] Yang M, Hu Y, Lodhi M, McCombie WR, Ma H (1999). The *Arabidopsis SKP1-LIKE1* gene is essential for male meiosis and may control homologue separation. PNAS..

[42] Pelaz S, Ditta G, Baumann E, Wisman E, Yanofsky MF (2000). B and C floral organ identity functions require *SEPALLATA* MADS-box genes. Nature..

[43] Wang PTC, Garcin PO, Fu M, Masoudi M, St-Pierre P, Panté N (2015). Distinct mechanisms controlling rough and smooth endoplasmic reticulum contacts with mitochondria. J Cell Sci.

[44] Wang M, Kaufman R (2016). Protein misfolding in the endoplasmic reticulum as a conduit to human disease. Nature..

[45] Monteith GR, Prevarskaya N, Roberts-Thomson SJ (2017). The calcium-cancer signaling nexus. Nat Rev Cancer.

[46] Braunger K, Pfeffer S, Shrimal S, Gilmore R, Berninghausen O, Mandon EC (2018). Structural basis for coupling protein transport and N-glycosylation at the mammalian endoplasmic reticulum. Science..

[47] Quon E, Sere YY, Chauhan N, Johansen J, Sullivan DP, Dittman JS (2018). Endoplasmic reticulum-plasma membrane contact sites integrate sterol and phospholipid regulation. PLoS Biol.

[48] Li H, Ericsson M, Rabasha B, Budnik B, Chan SH, Freinkman E (2019). Phosphogluconate dehydrogenase links cytosolic carbohydrate metabolism to protein secretion via modulation of glutathione levels. Cell Chem Biol.

[49] Wang L, Xu Y, Rogers H, Saidi L, Noguchi CT, Li H (2020). UFMylation of RPL26 links translocation-associated quality control to endoplasmic reticulum protein homeostasis. Cell Res.

[50] Keestra-Gounder AM, Byndloss MX, Seyffert N, Young BM, Chávez-Arroyo A, Tsai AY (2016). NOD1 and NOD2 signalling links ER stress with inflammation. Nature..

[51] Deng Y, Srivastava R, Howell SH (2013). Protein kinase and ribonuclease domains of IRE1 confer stress tolerance, vegetative growth, and reproductive development in *Arabidopsis*. PNAS..

[52] Stalder L, Heusermann W, Sokol L, Trojer D, Wirz J, Hean J (2013). The rough endoplasmatic reticulum is a central nucleation site of siRNA-mediated RNA silencing. EMBO J.

[53] Barman B, Bhattacharyya SN (2015). mRNA targeting to endoplasmic reticulum precedes ago protein interaction and microRNA (miRNA)-mediated translation repression in mammalian cells. J Biol Chem.

[54] Li S, Liu L, Zhuang X, Yu Y, Liu X, Cui X (2013). MicroRNAs inhibit the translation of target mRNAs on the endoplasmic reticulum in *Arabidopsis*. Cell..

[55] Brodersen P, Sakvarelidze-Achard L, Bruun-Rasmussen M, Dunoyer P, Yamamoto YY, Sieburth L (2008). Widespread translational inhibition by plant miRNAs and siRNAs. Science..

[56] Yang L, Wu G, Poethig RS (2012). Mutations in the GW-repeat protein SUO reveal a developmental function for microRNA-mediated translational repression in *Arabidopsis*. PNAS..

[57] Schwartz BW, Yeung EC, Meinke DW (1994). Disruption of morphogenesis and transformation of the suspensor in abnormal suspensor mutants of *Arabidopsis*. Development..

[58] Aukerman MJ, Sakai H (2003). Regulation of flowering time and floral organ identity by a microRNA and its *APETALA2*-like target genes. Plant Cell.

[59] Kasschau KD, Xie Z, Allen E, Llave C, Chapman EJ, Krizan KA (2003). P1/HC-Pro, a viral suppressor of RNA silencing, interferes with *Arabidopsis* development and miRNA unction. Dev Cell.

[60] Chen X (2004). A microRNA as a translational repressor of *APETALA2* in *Arabidopsis* flower development. Science..

[61] Chen J, Li WX, Xie D, Peng JR, Ding SW (2004). Viral virulence protein suppresses RNA silencing-mediated defense but upregulates the role of microRNA in host gene expression. Plant Cell.

[62] Sunkar R, Kapoor A, Zhu JK (2006). Posttranscriptional induction of two Cu/Zn superoxide dismutase genes in *Arabidopsis* is mediated by downregulation of miR398 and important for oxidative stress tolerance. Plant Cell.

[63] Gandikota M, Birkenbihl RP, Höhmann S, Cardon GH, Saedler H, Huijser P (2007). The miRNA156/157 recognition element in the 3′ UTR of the *Arabidopsis* SBP box gene *SPL3* prevents early flowering by translational inhibition in seedlings. Plant J.

[64] Jouannet V, Moreno AB, Elmayan T, Vaucheret H, Crespi MD, Maizel A (2012). Cytoplasmic *Arabidopsis* AGO7 accumulates in membrane-associated siRNA bodies and is required for ta-siRNA biogenesis. EMBO J.

[65] Araki S, Le NT, Koizumi K, Villar-Briones A, Nonomura KI, Endo M (2020). miR2118-dependent U-rich phasiRNA production in rice anther wall development. Nat Commun.

[66] Addo-Quaye C, Eshoo TW, Bartel DP, Axtell MJ (2008). Endogenous siRNA and miRNA targets identified by sequencing of the *Arabidopsis* degradome. Curr Biol.

[67] Li YF, Sunkar R (2013). Global identification of small RNA targets in plants by sequencing sliced ends of messenger RNAs. Methods Mol Biol.

[68] Zhai J, Arikit S, Simon SA, Kingham BF, Meyers BC (2014). Rapid construction of parallel analysis of RNA end (PARE) libraries for Illumina sequencing. Methods..

[69] Martin M (2011). Cutadapt removes adapter sequences from high-throughput sequencing reads. EMBnet J.

[70] Johnson NR, Yeoh JM, Coruh C, Axtell MJ (2016). Improved placement of multi-mapping small RNAs. G3.

[71] Love MI, Huber W, Anders S (2014). Moderated estimation of fold change and dispersion for RNA-seq data with DESeq2. Genome Biol.

[72] Howell MD, Fahlgren N, Chapman EJ, Cumbie JS, Sullivan CM, Givan SA (2007). Genome-wide analysis of the RNA-DEPENDENT RNA POLYMERASE6/DICER-LIKE4 pathway in *Arabidopsis* reveals dependency on miRNA- and tasiRNA-directed targeting. Plant Cell.

[73] De Paoli E, Dorantes-Acosta A, Zhai J, Accerbi M, Jeong DH, Park S (2009). Distinct extremely abundant siRNAs associated with cosuppression in petunia. RNA..

[74] Langmead B, Salzberg SL (2012). Fast gapped-read alignment with bowtie 2. Nat Methods.

[75] Cai Q, Liang C, Wang S, Hou Y, Gao L, Liu L (2018). The disease resistance protein SNC1 represses the biogenesis of microRNAs and phased siRNAs. Nat Commun.

[76] Yang X, You C, Wang X, Gao L, Mo B, Liu L (2020). Sequencing of polysome-associated sRNAs and 3′ cleavage fragments in maize and rice. Datasets. NCBI Sequence Read Archive.

