# Supplementary material for: Widespread occurrence of microRNA-mediated target cleavage on membrane-bound polysomes
Source: Genome Biol. 2021 Jan 5;22:15. doi: 10.1186/s13059-020-02242-6 (PMC7784310; doi:10.1186/s13059-020-02242-6)
Supplement: Supplementary file 1 — Additional file 1: Figure S1–S17. [file 13059_2020_2242_MOESM1_ESM.docx]

**Additional file 1**

**Table of contents**

**Fig. S1** Profiles for total polysomes and membrane-bound polysomes from different tissues of maize and rice along 15% – 60% sucrose gradients.

**Fig. S2** Effective isolation of total polysomes and membrane-bound polysomes from different tissues of maize and rice.

**Fig. S3** Clustering analysis for small RNA-seq datasets from input, total polysome and membrane-bound polysome samples from different tissues of maize and rice.

**Fig. S4** Distinct subcellular partitioning of 21-nt/22-nt and 24-nt small RNAs in maize seedling shoots, immature ears and rice seedling shoots.

**Fig. S5** Composition of genomic features that give rise to 21-nt, 22-nt and 24-nt small RNAs (sRNAs) in input (Total), total polysome (TP) and membrane-bound polysome (MBP) samples from different tissues of maize and rice.

**Fig. S6** Distinct subcellular partitioning of 22-nt and 24-nt transposable element-derived siRNAs in different tissues of maize and rice.

**Fig. S7** Retrotransposons and DNA transposons contribute differentially to polysome association of transposable element-derived siRNAs in maize seedling shoots.

**Fig. S8** Retrotransposons and DNA transposons contribute differentially to polysome association of transposable element-derived siRNAs in maize immature ears.

**Fig. S9** Overaccumulation of miRNAs on membrane-bound polysomes in maize seedling shoots, immature ears and rice seedling shoots.

**Fig. S10** Detection of miRNA-mediated target cleavage in input, total polysome and membrane-bound polysome samples from maize seedling shoots, immature ears and rice seedling shoots.

**Fig. S11** Overaccumulation of miRNA-mediated target cleavage on membrane-bound polysomes correlates with the subcellular distribution of miRNAs in maize and rice.

**Fig. S12** Reproductive 21-nt phasiRNAs are overwhelmingly enriched in maize immature tassels and rice immature panicles.

**Fig. S13** Reproductive 24-nt phasiRNAs are overwhelmingly enriched in maize immature tassels and rice immature panicles.

**Fig. S14** Abundance of reproductive *PHAS* precursors in input, total polysome and membrane-bound polysome samples from maize immature tassels and rice immature panicles.

**Fig. S15** PARE 3’ cleavage fragment coverage for reproductive *21PHAS* and *24PHAS* precursors targeted by miR2118 and miR2275, respectively, in maize immature tassels and rice immature panicles.

**Fig. S16** Distinct subcellular partitioning of reproductive 21-nt and 24-nt phasiRNAs in maize immature tassels and rice immature panicles.

**Fig. S17** Expression of genes targeted by reproductive 21-nt phasiRNAs in maize and rice.

**
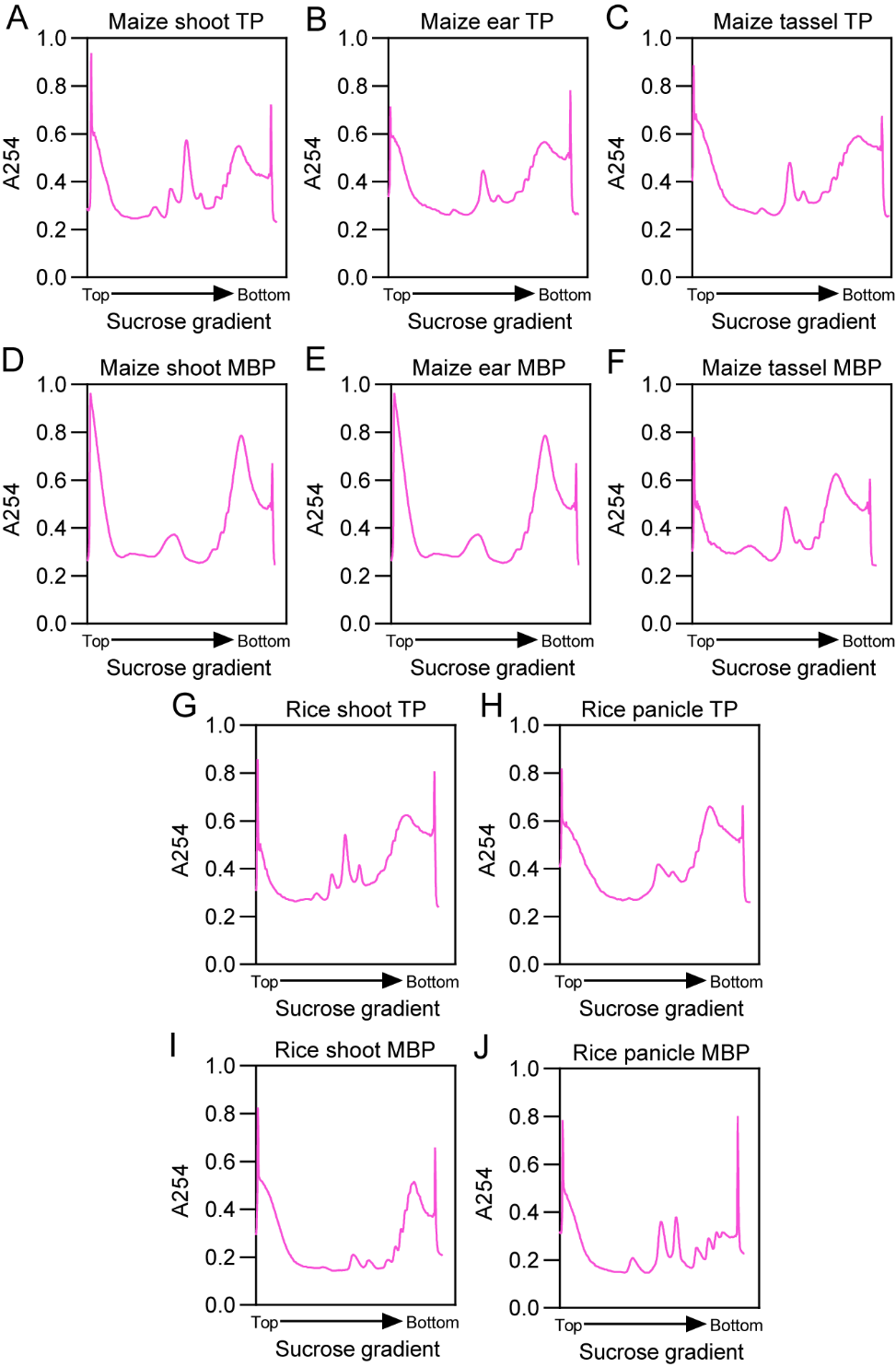
**

**Fig. S1** Profiles for total polysomes and membrane-bound polysomes from different tissues of maize and rice along 15% – 60% sucrose gradients. **(A–C, G–H)** Profiles of total polysome (TP) samples isolated from maize seedling shoots (A), immature ears (B), immature tassels (C), rice seedling shoots (G) and immature panicles (H). **(D–F, I–J)** Profiles of membrane-bound polysome (MBP) samples isolated from maize seedling shoots (D), immature ears (E), immature tassels (F), rice seedling shoots (I) and immature panicles (J). Polysome profiles were obtained by recording absorbance at 254 nm (A254) during fractionation from top to bottom of gradients.

**
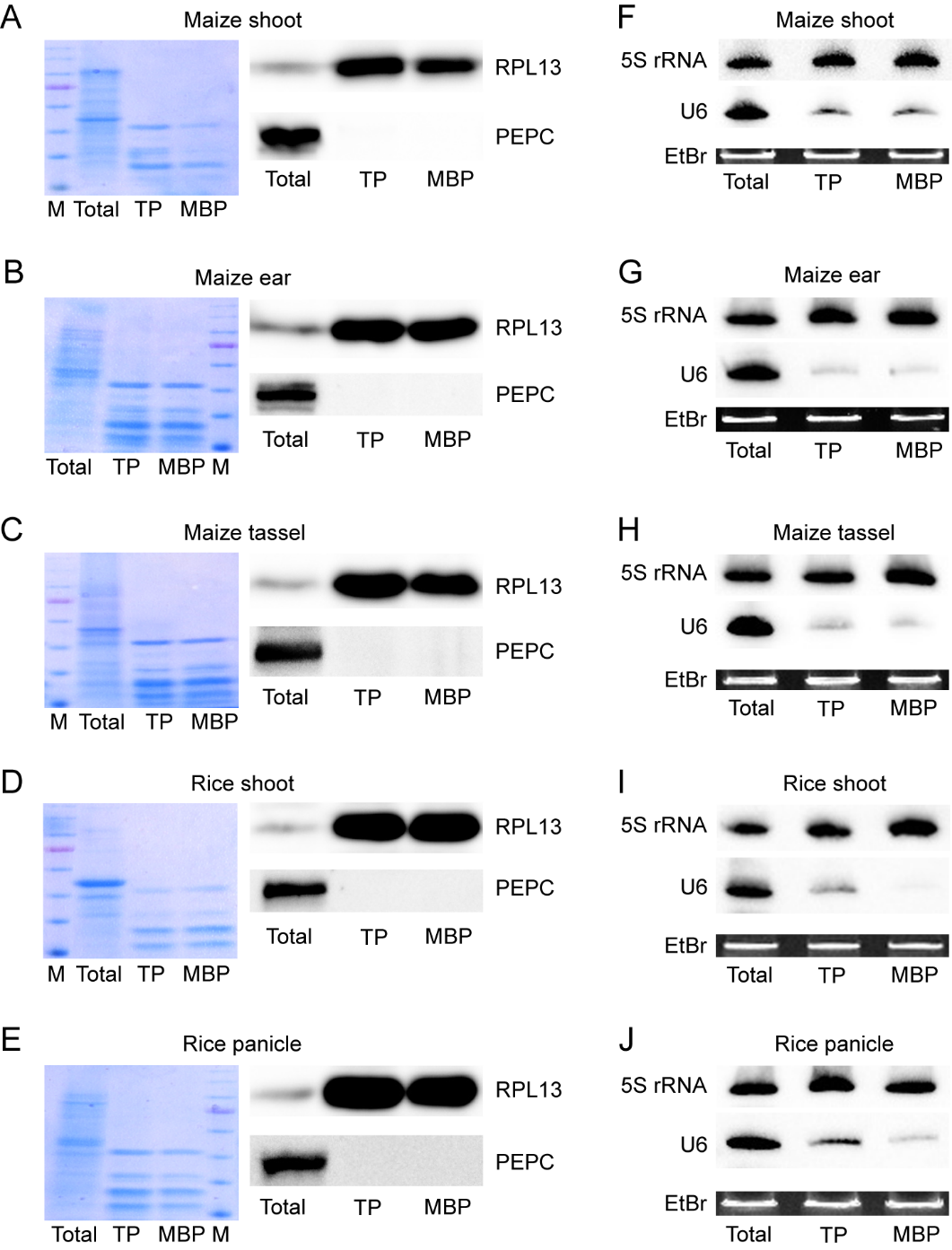
**

**Fig. S2** Effective isolation of total polysomes and membrane-bound polysomes from different tissues of maize and rice. **(A–E)** Detection of ribosomal protein L13 (RPL13) and phosphoenolpyruvate carboxylase (PEPC) by Western blotting in input (Total), total polysome (TP) and membrane-bound polysome (MBP) samples from maize seedling shoots (A), immature ears (B), immature tassels (C), rice seedling shoots (D) and immature panicles (E). RPL13 is a component of the 60S ribosome subunit and expected to be in the polysomal fractions, while PEPC is a cytosolic protein and expected to be depleted from the polysomal fractions. Images of protein staining by Coomassie Brilliant Blue are shown on the left to indicate equal loading. “M”: molecular weight standards. **(F–J)** Detection of 5S rRNA and U6 by Northern blotting in Total, TP and MBP samples from maize seedling shoots (F), immature ears (G), immature tassels (H), rice seedling shoots (I) and immature panicles (J). 5S rRNA is expected to be in the polysome and input samples, while U6 is a small nuclear RNA and expected to be depleted from polysomal fractions. Ethidium bromide (EtBr)-stained rRNA was used as a loading control.

**
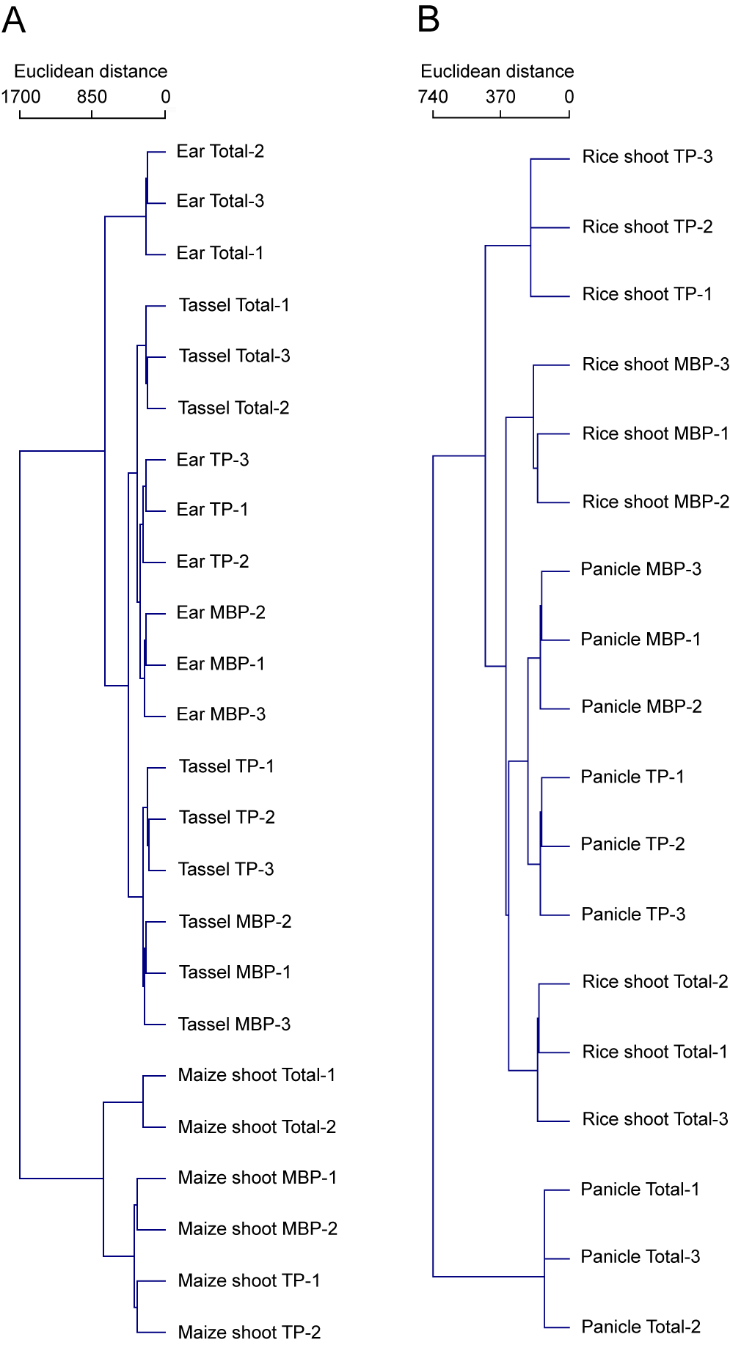
**

**Fig. S3** Clustering analysis for small RNA-seq datasets from input, total polysome and membrane-bound polysome samples from different tissues of maize and rice. **(A)** Clustering analysis for small RNA (sRNA)-seq datasets from input (Total), total polysome (TP) and membrane-bound polysome (MBP) samples from maize seedling shoots, immature ears and immature tassels. **(B)** Clustering analysis for sRNA-seq datasets from Total, TP and MBP samples from rice seedling shoots and immature panicles. “Total-1, 2, 3”, “TP-1, 2, 3” and “MBP-1, 2, 3” represent different biological repeats. The scale bars indicate Euclidean distances between samples measured by DESeq2 normalized read counts.

**
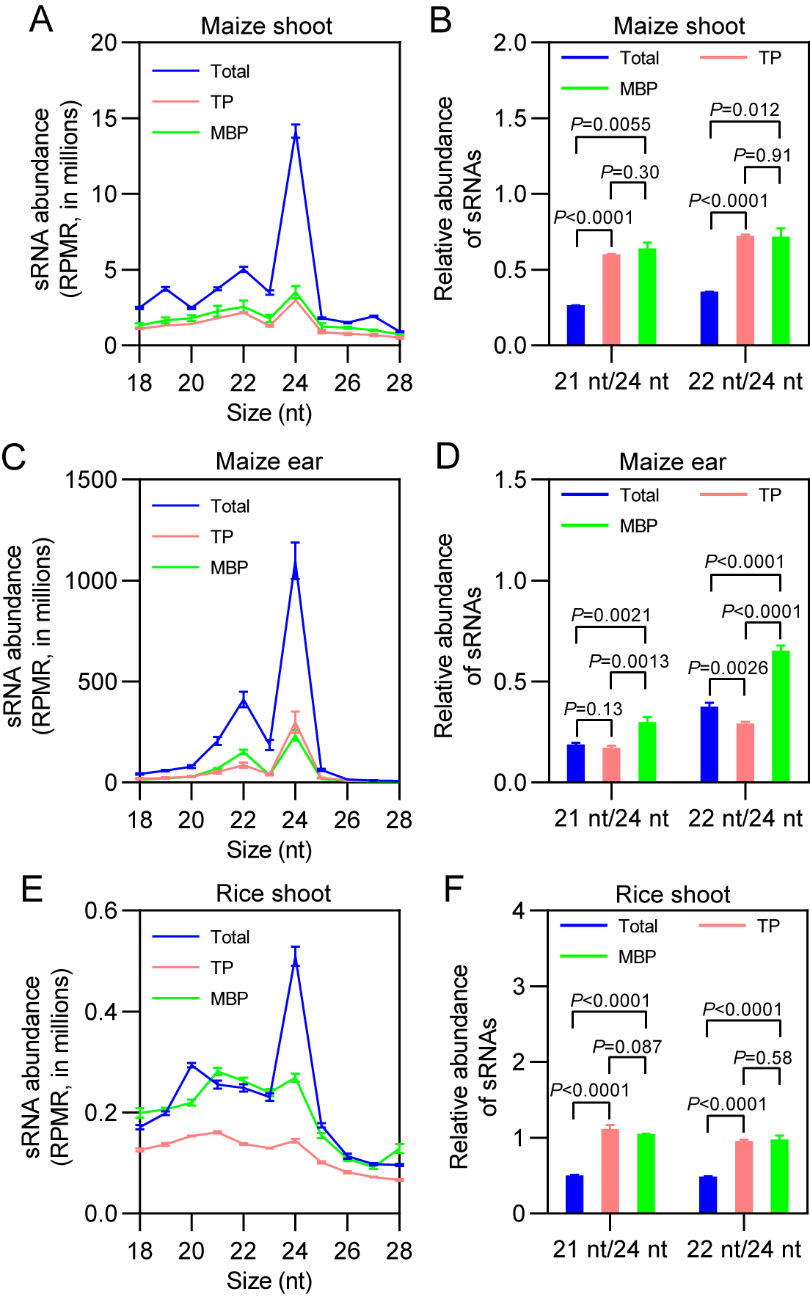
**

**Fig. S4** Distinct subcellular partitioning of 21-nt/22-nt and 24-nt small RNAs in maize seedling shoots, immature ears and rice seedling shoots. **(A, C, E)** Size distribution of small RNAs (sRNAs) in input (Total), total polysome (TP) and membrane-bound polysome (MBP) samples from maize seedling shoots (A), immature ears (C) and rice seedling shoots (E). **(B, D, F)** Ratios of sRNA abundance between 21-nt and 24-nt and between 22-nt and 24-nt classes in maize seedling shoots (B), immature ears (D) and rice seedling shoots (F). Skewed distribution of 21-nt and 22-nt sRNAs towards TP and MBP (B and F) and towards MBP (D) relative to Total samples is observed. sRNA abundance is displayed as mean ± standard deviation (SD) of two biological repeats for maize seedling shoots (A and B), and of three biological repeats for maize immature ears (C and D) and rice seedling shoots (E and F). “RPMR” is short for “reads per million rRNA fragments” (A, C and E). Comparisons between Total, TP and MBP were performed by two-tailed unpaired *t*-test, and *P*-values are displayed above the corresponding comparisons (B, D and F).

**
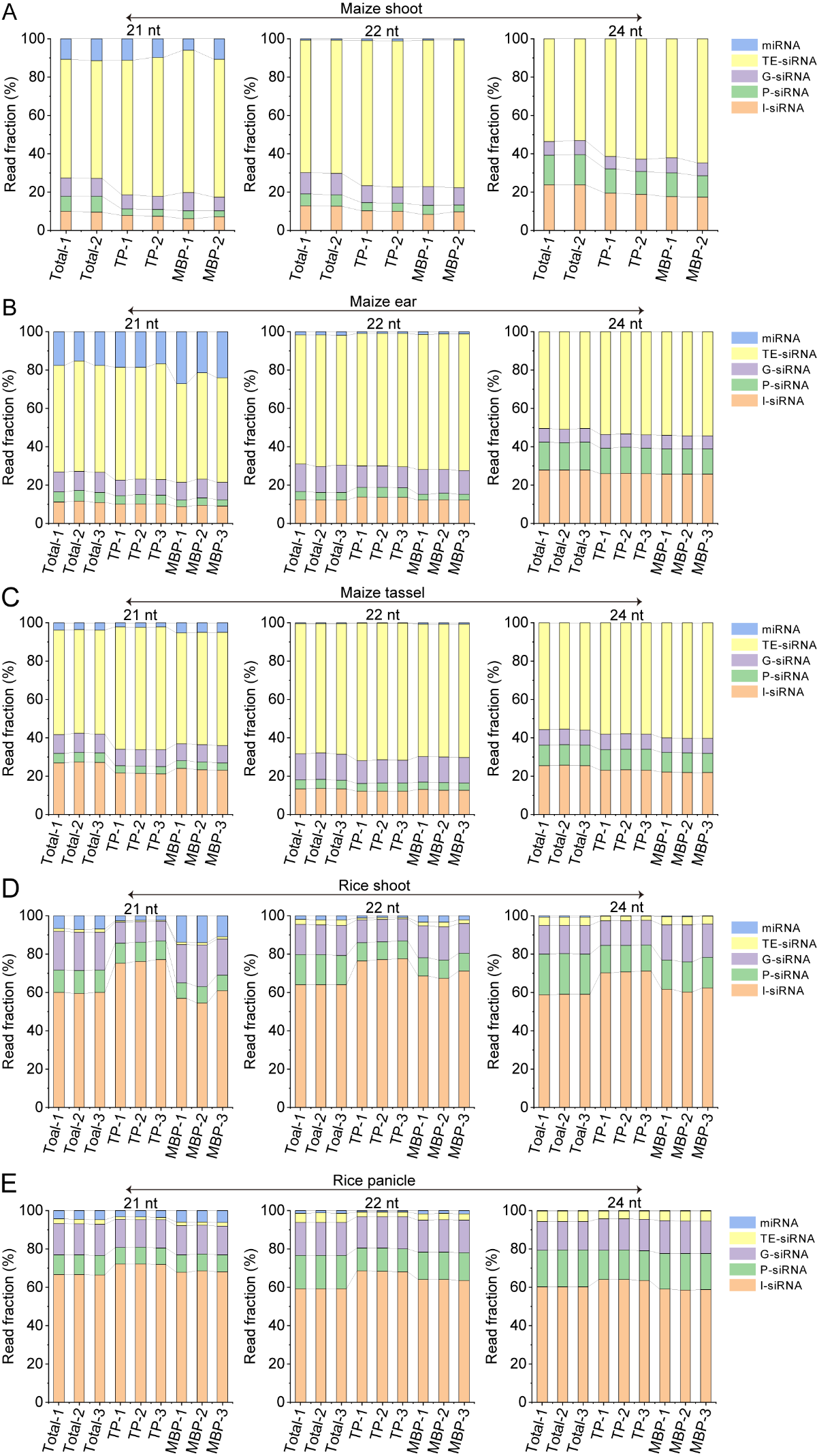
**

**Fig. S5** Composition of genomic features that give rise to 21-nt, 22-nt and 24-nt small RNAs (sRNAs) in input (Total), total polysome (TP) and membrane-bound polysome (MBP) samples from different tissues of maize and rice. **(A)** Maize seedling shoots. **(B)** Maize immature ears. **(C)** Maize immature tassels. **(D)** Rice seedling shoots. **(E)** Rice immature panicles. “Total-1, 2, 3”, “TP-1, 2, 3” and “MBP-1, 2, 3” represent different biological repeats. TE-siRNA: transposable element (TE)-derived siRNA; G-siRNA: gene-derived siRNA; P-siRNA: promoter-derived siRNA; I-siRNA: intergenic region-derived siRNA.

**
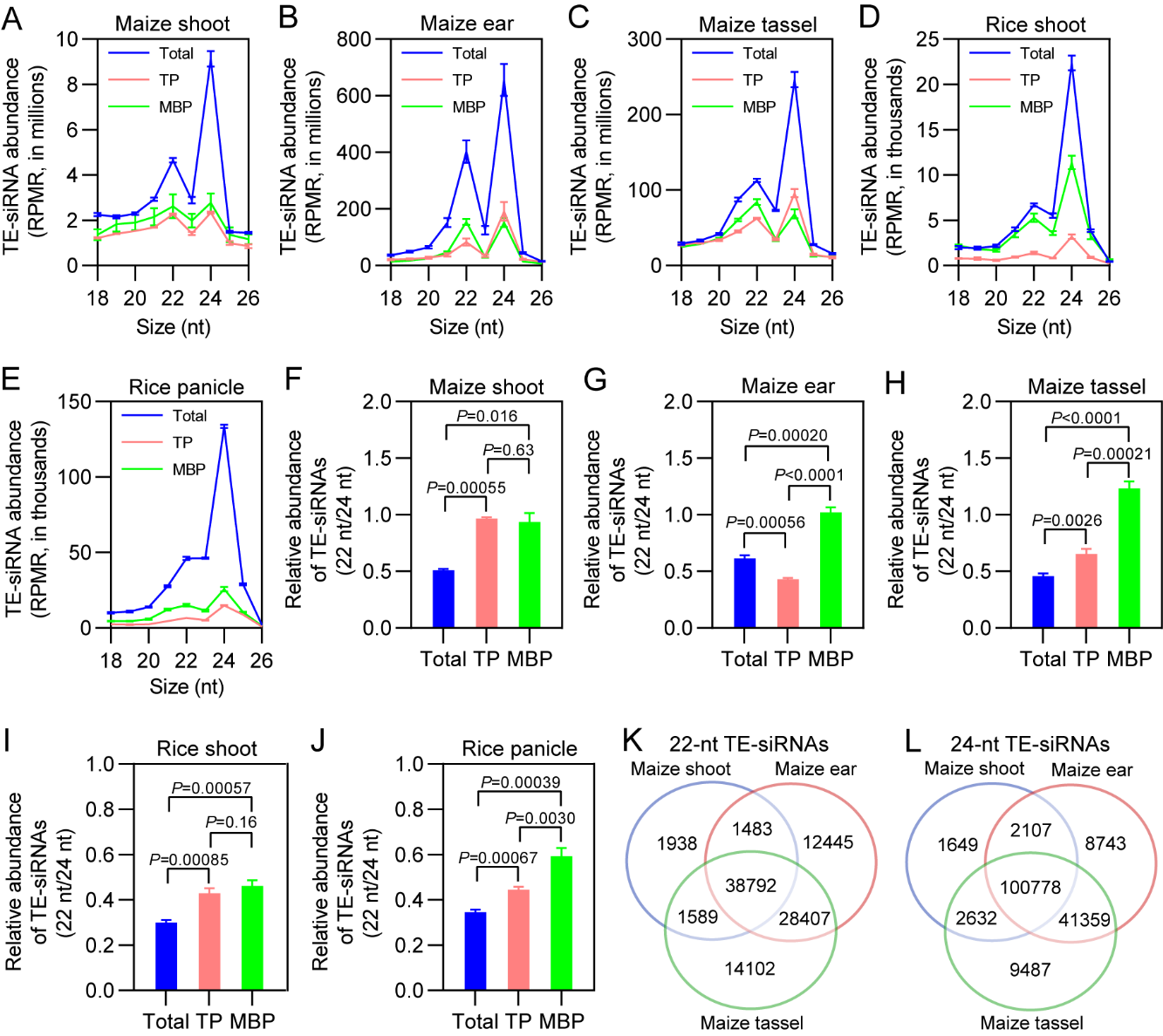
**

**Fig. S6** Distinct subcellular partitioning of 22-nt and 24-nt transposable element-derived siRNAs in different tissues of maize and rice. **(A–E)** Size distribution of transposable element (TE)-derived siRNAs (TE-siRNAs) in input (Total), total polysome (TP) and membrane-bound polysome (MBP) samples from maize seedling shoots (A), immature ears (B), immature tassels (C), rice seedling shoots (D) and immature panicles (E). **(F–J)** Ratios of TE-siRNA abundance between 22-nt and 24-nt classes in maize seedling shoots (F), immature ears (G), immature tassels (H), rice seedling shoots (I) and immature panicles (J). Skewed distribution of 22-nt TE-siRNAs towards TP and MBP (F and H–J) and towards MBP (G) relative to Total samples is observed. TE-siRNA abundance is displayed as mean ± standard deviation (SD) of two biological repeats for maize seedling shoots (A and F) and of three biological repeats for maize immature ears (B and G), immature tassels (C and H), rice seedling shoots (D and I) and immature panicles (E and J). “RPMR” is short for “reads per million rRNA fragments” (A–E). **(K–L)** Overlap of 22-nt (K) and 24-nt (L) siRNA-generating TE loci among maize seedling shoots, immature ears and immature tassels.

**
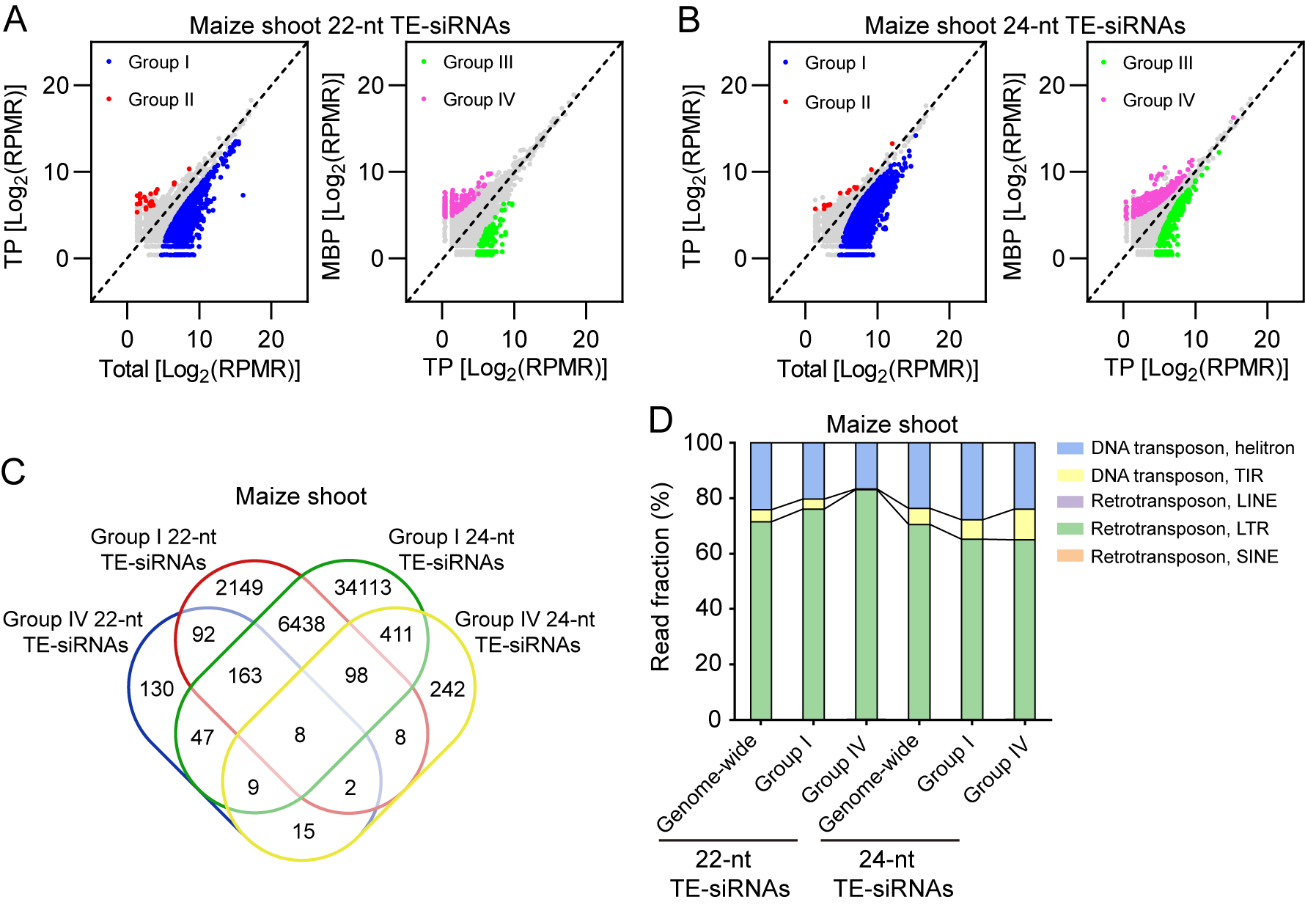
**

**Fig. S7** Retrotransposons and DNA transposons contribute differentially to polysome association of transposable element-derived siRNAs in maize seedling shoots. **(A)** Identification of differentially accumulated 22-nt transposable element (TE)-derived siRNAs (TE-siRNAs) between total polysome (TP) and input (Total) (left panel), and between membrane-bound polysome (MBP) and TP (right panel). **(B)** Identification of differentially accumulated 24-nt TE-siRNAs between TP and Total (left panel), and between MBP and TP (right panel). TE-siRNA abundance is displayed as the mean of two biological repeats. “RPMR” is short for “reads per million rRNA fragments”. **(C)** Overlap of TE loci that generated differentially accumulated TE-siRNAs in the various comparisons in (A) and (B). **(D)** Contributions of retrotransposons and DNA transposons to the differentially accumulated 22-nt and 24-nt TE-siRNAs. “Genome-wide” denotes the contributions of the transposon types to total TE-siRNAs. LINE: long interspersed nuclear element; LTR: long terminal repeat; SINE: short interspersed nuclear element; TIR: terminal inverted repeat. The cutoff parameters for differentially accumulated TE-siRNAs are fold change >= 2 and *P*-value <= 0.05. “Group I”, “Group II”, “Group III” and “Group IV” represent “TE loci with 22-nt or 24-nt siRNAs that were polysome-depleted”, “TE loci producing 22-nt or 24-nt siRNAs that were polysome-associated”, “TE loci with 22-nt or 24-nt siRNAs that were likely on free polysomes (FPs)” and “TE loci with 22-nt or 24-nt siRNAs enriched on MBPs”, respectively.

**
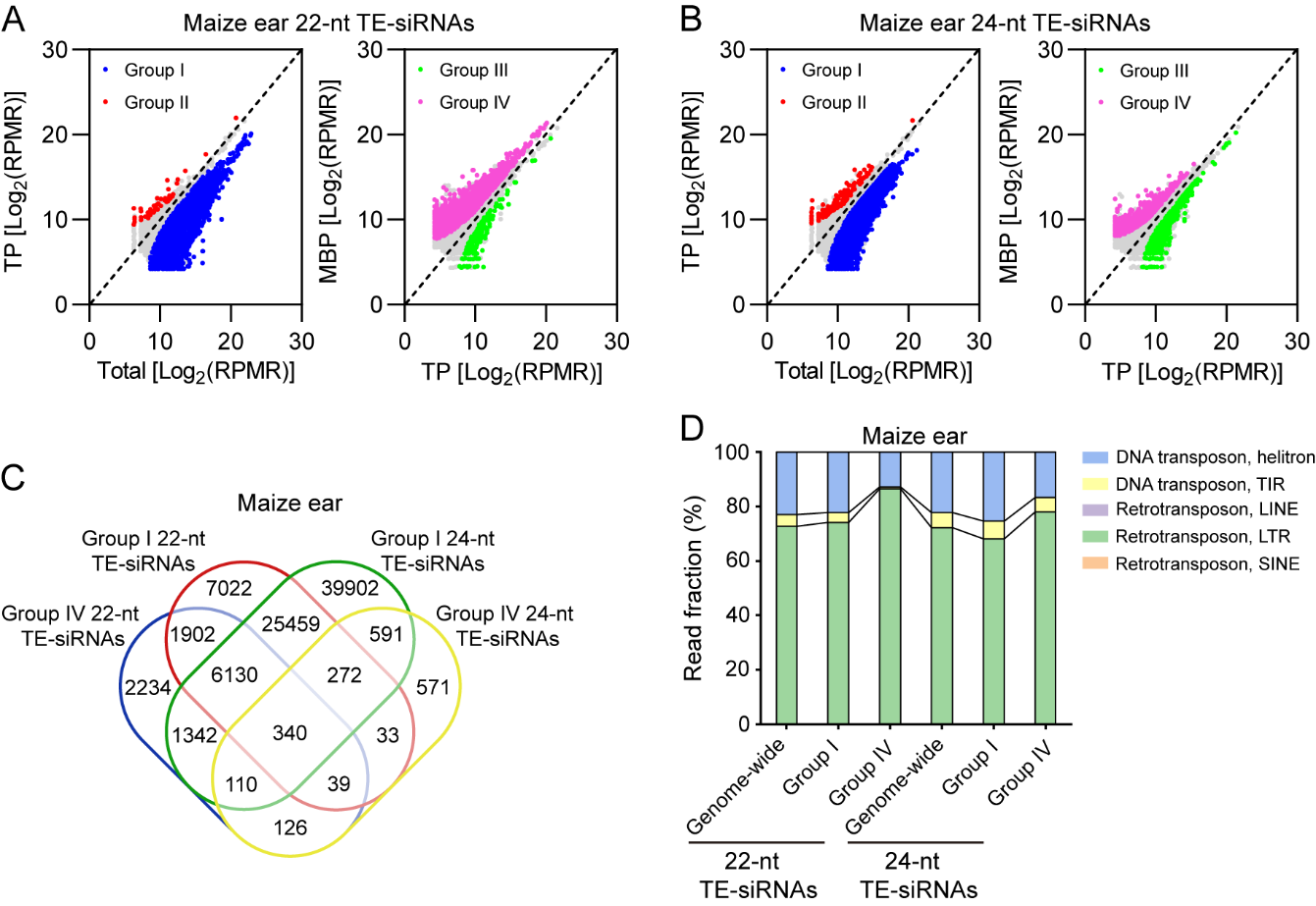
**

**Fig. S8** Retrotransposons and DNA transposons contribute differentially to polysome association of transposable element-derived siRNAs in maize immature ears. **(A)** Identification of differentially accumulated 22-nt transposable element (TE)-derived siRNAs (TE-siRNAs) between total polysome (TP) and input (Total) (left panel), and between membrane-bound polysome (MBP) and TP (right panel). **(B)** Identification of differentially accumulated 24-nt TE-siRNAs between TP and Total (left panel), and between MBP and TP (right panel). TE-siRNA abundance is displayed as the mean of three biological repeats. “RPMR” is short for “reads per million rRNA fragments”. **(C)** Overlap of TE loci that generated differentially accumulated TE-siRNAs in the various comparisons in (A) and (B). **(D)** Contributions of retrotransposons and DNA transposons to the differentially accumulated 22-nt and 24-nt TE-siRNAs. “Genome-wide” denotes the contributions of the transposon types to total TE-siRNAs. LINE: long interspersed nuclear element; LTR: long terminal repeat; SINE: short interspersed nuclear element; TIR: terminal inverted repeat. The cutoff parameters for differentially accumulated TE-siRNAs are fold change >= 2 and *P*-value <= 0.05. “Group I”, “Group II”, “Group III” and “Group IV” represent “TE loci with 22-nt or 24-nt siRNAs that were polysome-depleted”, “TE loci producing 22-nt or 24-nt siRNAs that were polysome-associated”, “TE loci with 22-nt or 24-nt siRNAs that were likely on free polysomes (FPs)” and “TE loci with 22-nt or 24-nt siRNAs enriched on MBPs”, respectively.

**
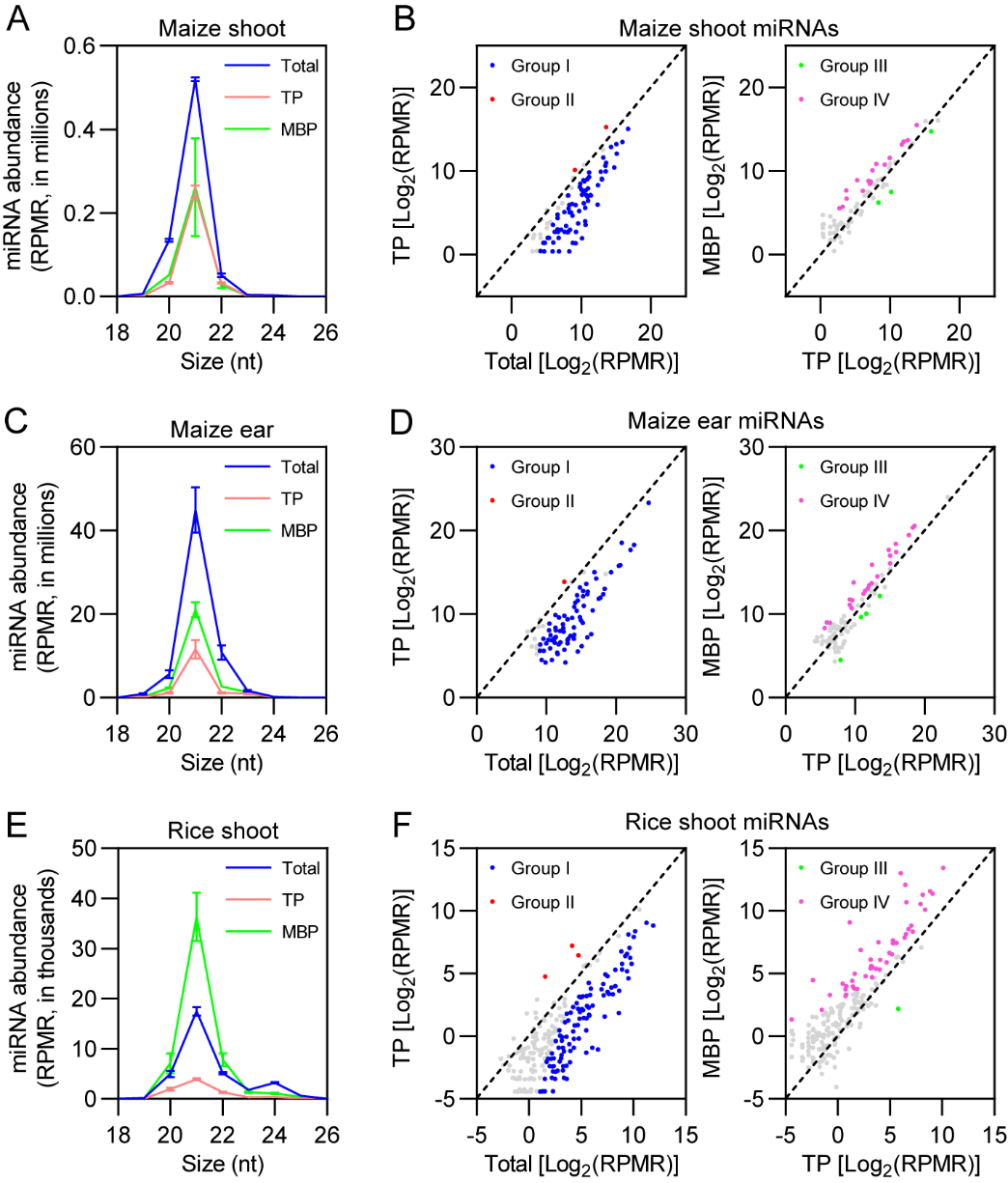
**

**Fig. S9** Overaccumulation of miRNAs on membrane-bound polysomes in maize seedling shoots, immature ears and rice seedling shoots. **(A, C, E)** Size and abundance of all miRNAs in maize seedling shoots (A), immature ears (C) and rice seedling shoots (E). **(B, D, F)** Identification of differentially accumulated miRNAs between total polysome (TP) and input (Total) (left panels), and between membrane-bound polysome (MBP) and TP (right panels) in maize seedling shoots (B), immature ears (D) and rice seedling shoots (F). miRNA abundance is displayed as mean ± standard deviation (SD) (A, C and E) or the mean of two biological repeats for maize seedling shoots (B), and of three biological repeats for maize immature ears (D) and rice seedling shoots (F). “nt” stands for “nucleotide” (A, C and E), and “RPMR” is short for “reads per million rRNA fragments”. The cutoff parameters for differentially accumulated miRNAs are fold change >= 2 and *P*-value <= 0.05. “Group I”, “Group II”, “Group III” and “Group IV” represent “miRNAs that were polysome-depleted”, “miRNAs enriched on polysomes”, “miRNAs that were associated with polysomes but MBP-depleted” and “miRNAs that were MBP-enriched”, respectively (B, D and F).

**
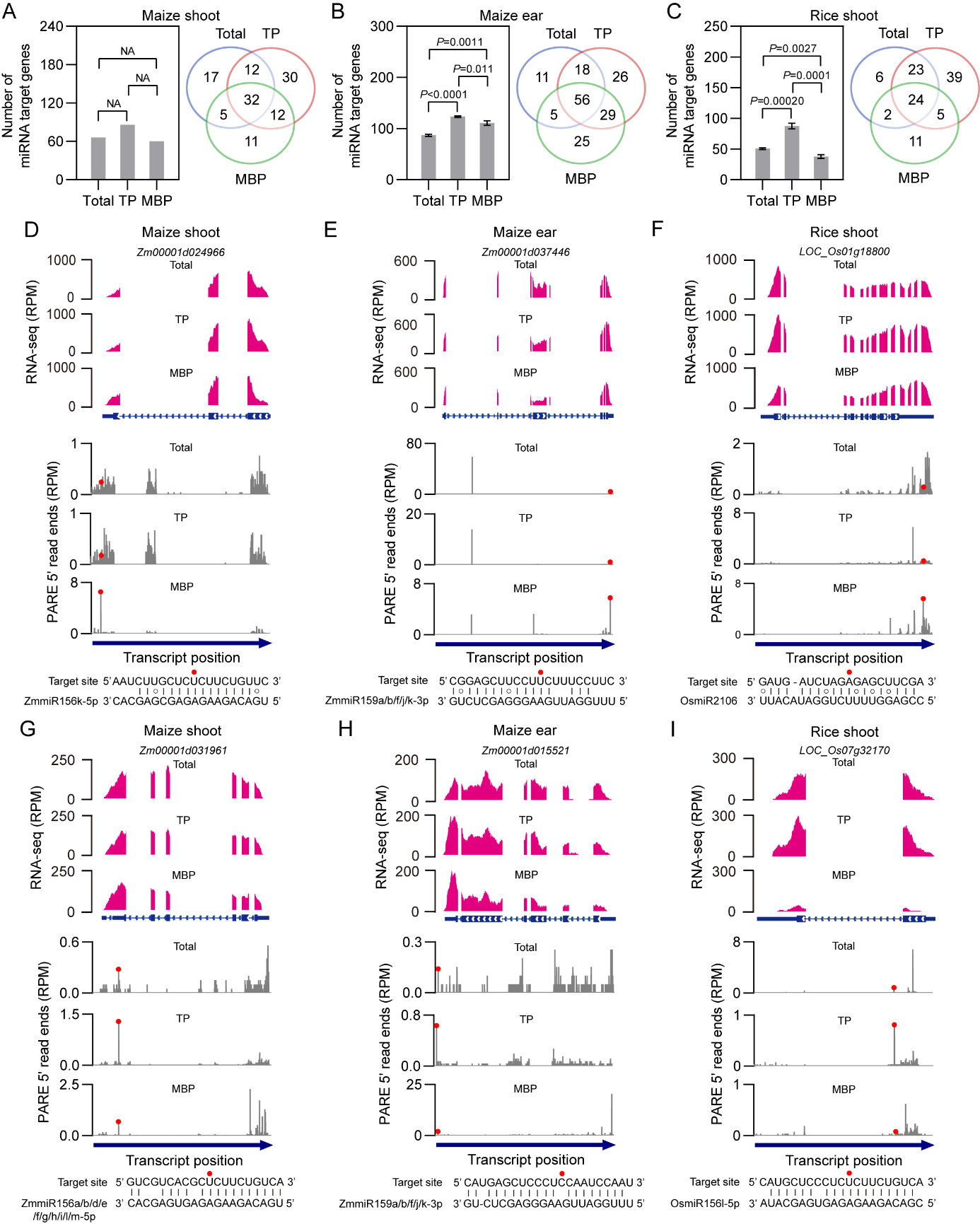
**

**Fig. S10** Detection of miRNA-mediated target cleavage in input, total polysome and membrane-bound polysome samples from maize seedling shoots, immature ears and rice seedling shoots. **(A–C)** Number (left panels) and overlap of identified miRNA target genes (right panels) in input (Total), total polysome (TP) and membrane-bound polysome (MBP) samples from maize seedling shoots (A), immature ears (B) and rice seedling shoots (C). Cleavage sites that are classified as category 0 with *P*-value <= 0.05 in at least two biological repeats were filtered as miRNA target sites. Category 0 means that the 3’ cleavage fragments with the maximum read count (>1) are mapped to only one indicated position on the transcript. Number of miRNA target genes is displayed as mean ± standard deviation (SD) of two biological repeats for maize seedling shoots (left panel of A), and of three biological repeats for maize immature ears (left panel of B) and rice seedling shoots (left panel of C). Comparisons between Total, TP and MBP were performed by two-tailed unpaired *t*-test and *P*-values are displayed above the corresponding comparisons (left panels of B and C). “NA” is short for “not available” (left panel of A). **(D)** PolyA RNA-seq read (top panel) and PARE 3’ cleavage fragment (bottom panel) coverage for *Zm00001d024966*, an identified MBP-unique target transcript cleaved by miR156k-5p, in Total, TP and MBP samples from maize seedling shoots. **(E)** PolyA RNA-seq read (top panel) and PARE 3’ cleavage fragment (bottom panel) coverage for *Zm00001d037446*, an identified MBP-unique target transcript cleaved by miR159a/b/f/j/k-3p, in Total, TP and MBP samples from maize immature ears. **(F)** PolyA RNA-seq read (top panel) and PARE 3’ cleavage fragment (bottom panel) coverage for *LOC_Os01g18800*, an identified MBP-unique target transcript cleaved by miR2106, in Total, TP and MBP samples from rice seedling shoots. **(G)** PolyA RNA-seq read (top panel) and PARE 3’ cleavage fragment (bottom panel) coverage for *Zm00001d031961*, an identified TP-unique target transcript cleaved by miR156a/b/d/e/f/g/h/i/l/m-5p, in Total, TP and MBP samples from maize seedling shoots. **(H)** PolyA RNA-seq read (top panel) and PARE 3’ cleavage fragment (bottom panel) coverage for *Zm00001d015521*, an identified TP-unique target transcript cleaved by miR159a/b/f/j/k-3p, in Total, TP and MBP samples from maize immature ears. **(I)** PolyA RNA-seq read (top panel) and PARE 3’ cleavage fragment (bottom panel) coverage for *LOC_Os07g32170*, an identified TP-unique target transcript cleaved by miR156l-5p, in Total, TP and MBP samples from rice seedling shoots. The gene models are shown below the RNA-seq panels, with the thicker rectangles, lines and thinner rectangles representing exons, introns and UTR regions, respectively. In the PARE panels, the red dots indicate the cleavage sites on the transcripts targeted by miRNAs. The sequences of the target sites and the corresponding miRNAs are shown below the PARE panels. “PARE” and “RPM” are short for “parallel analysis of RNA ends” and “reads per million mapped reads”, respectively.

**
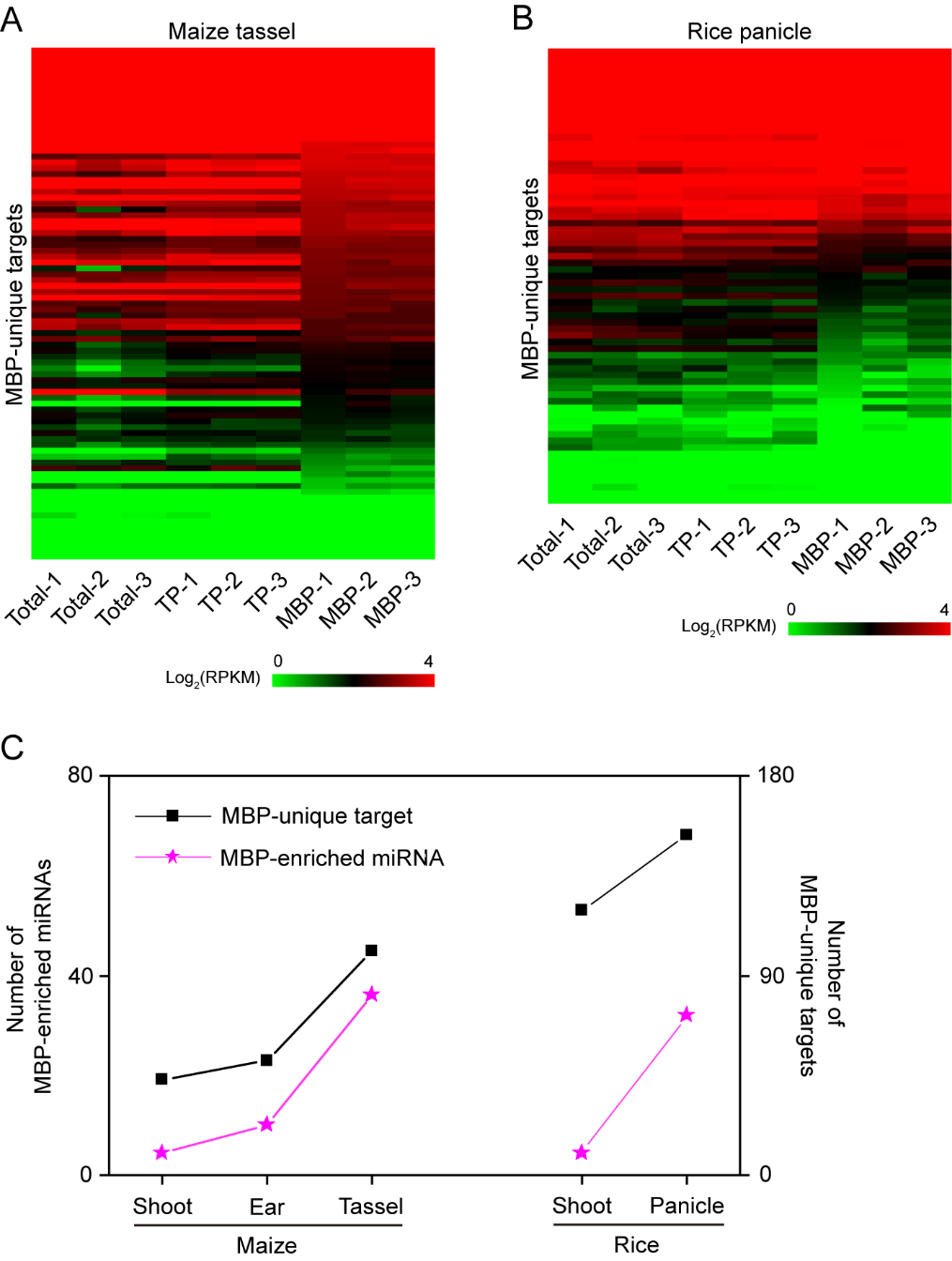
**

**Fig. S11** Overaccumulation of miRNA-mediated target cleavage on membrane-bound polysomes correlates with the subcellular distribution of miRNAs in maize and rice. **(A)** Abundance of membrane-bound polysome (MBP)-unique miRNA target transcripts in input (Total), total polysome (TP) and MBP samples from maize immature tassels. **(B)** Abundance of MBP-unique target transcripts in Total, TP and MBP samples from rice immature panicles. “Total-1, 2, 3”, “TP-1, 2, 3” and “MBP-1, 2, 3” represent different biological repeats. “RPKM” is short for “reads per kilobase of transcript per million mapped reads”. **(C)** Positive correlation between the number of MBP-enriched miRNAs and the number of MBP-unique target genes in maize and rice.

**
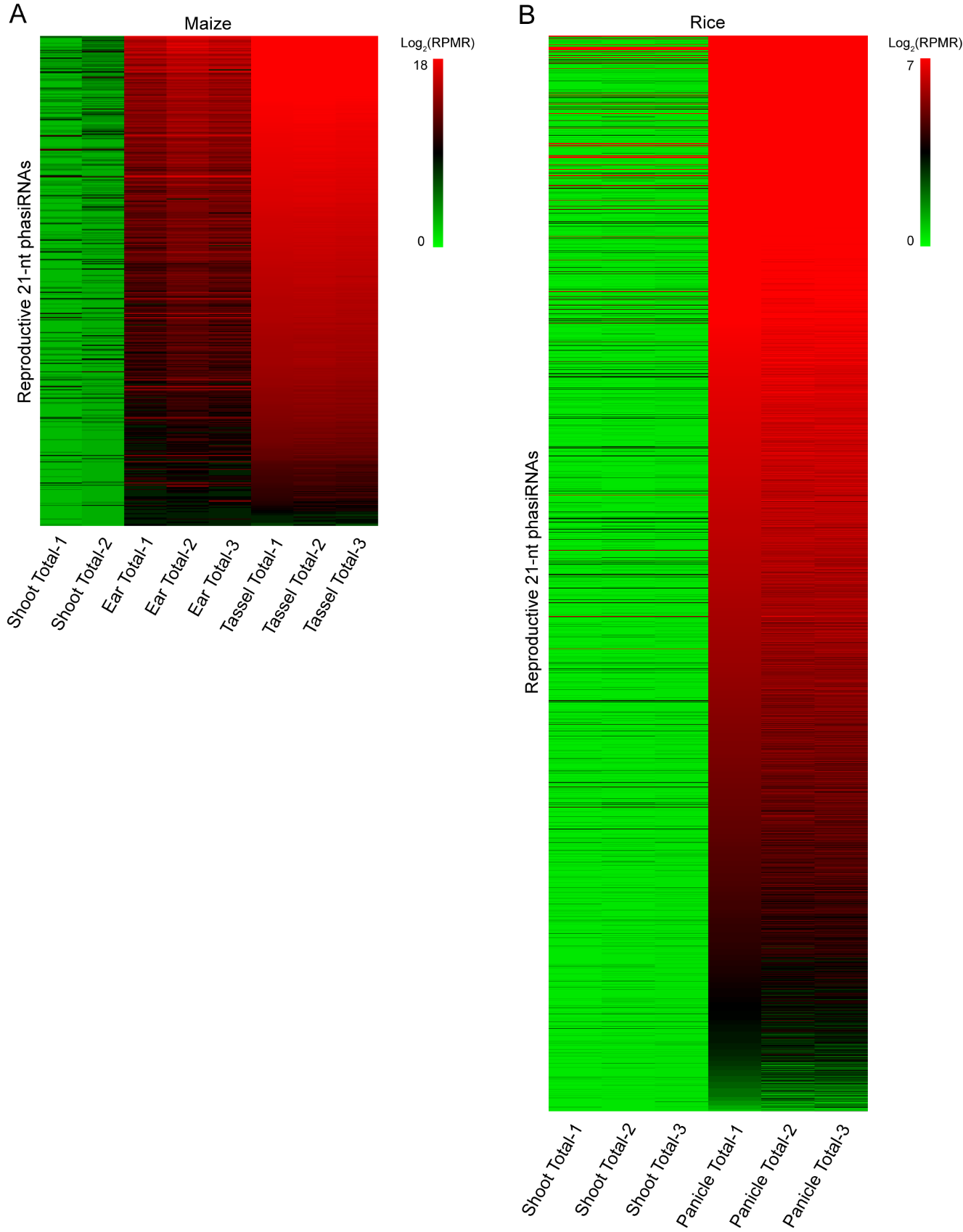
**

**Fig. S12** Reproductive 21-nt phasiRNAs are overwhelmingly enriched in maize immature tassels and rice immature panicles. **(A)** Abundance of reproductive 21-nt phasiRNAs in maize seedling shoots, immature ears and immature tassels. **(B)** Abundance of reproductive 21-nt phasiRNAs in rice seedling shoots and immature panicles. “Total-1”, “Total-2” and “Total-3” represent different biological repeats of total extracts without fractionation. “RPMR” is short for “reads per million rRNA fragments”.

**
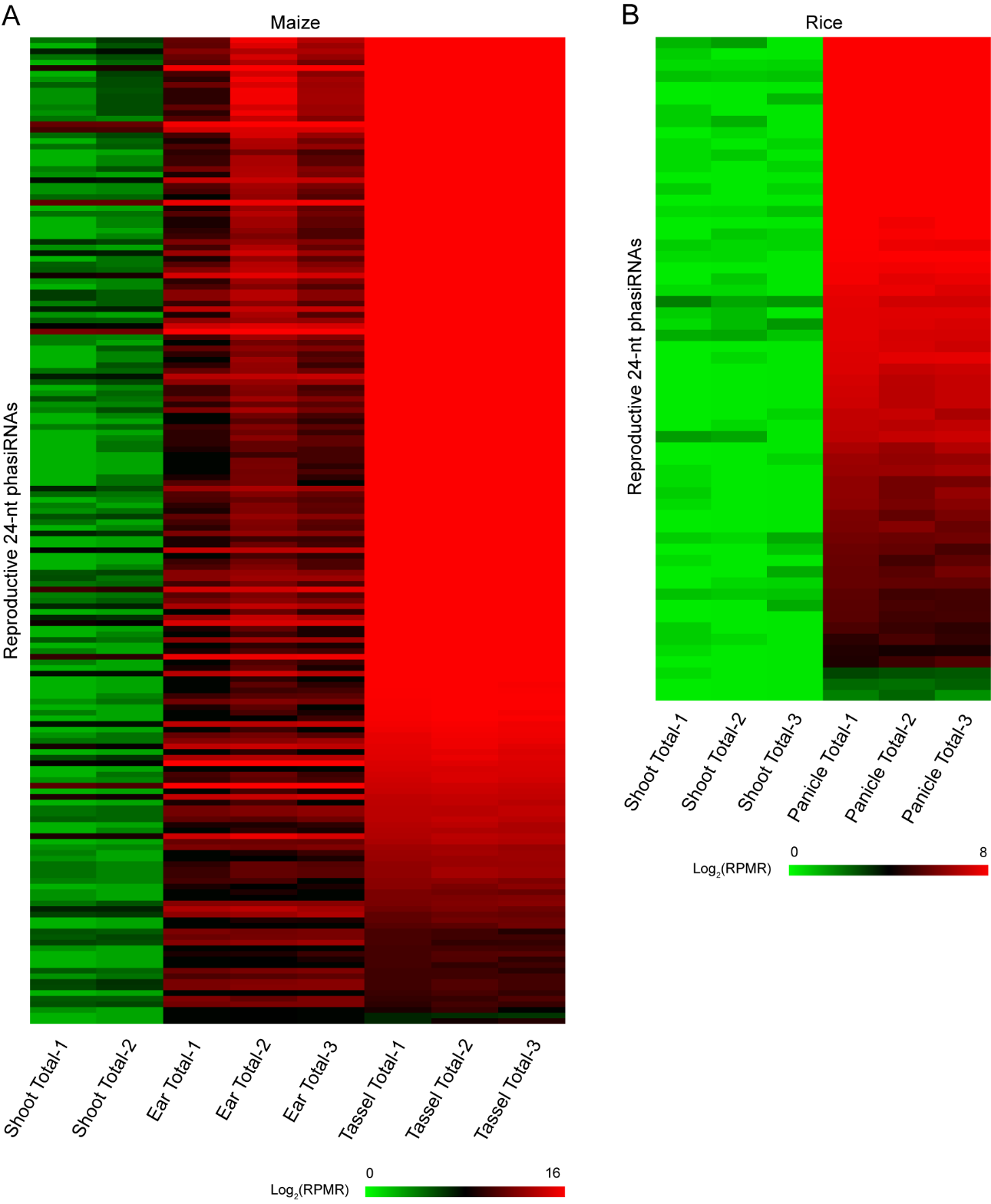
**

**Fig. S13** Reproductive 24-nt phasiRNAs are overwhelmingly enriched in maize immature tassels and rice immature panicles. **(A)** Abundance of reproductive 24-nt phasiRNAs in maize seedling shoots, immature ears and immature tassels. **(B)** Abundance of reproductive 24-nt phasiRNAs in rice seedling shoots and immature panicles. “Total-1”, “Total-2” and “Total-3” represent different biological repeats of total extracts without fractionation. “RPMR” is short for “reads per million rRNA fragments”.

**
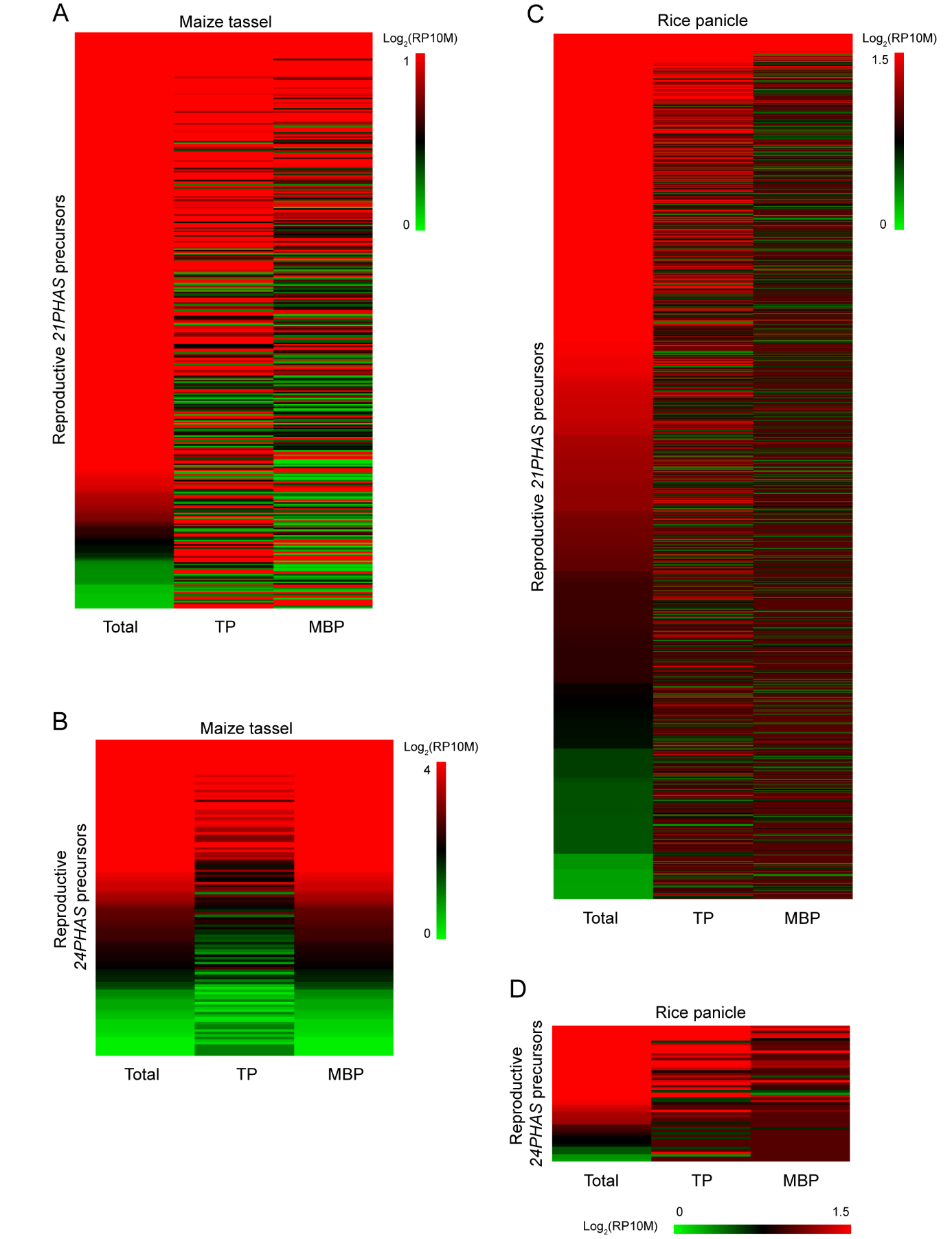
**

**Fig. S14** Abundance of reproductive *PHAS* precursors in input, total polysome and membrane-bound polysome samples from maize immature tassels and rice immature panicles. **(A, C)** Abundance of reproductive *21PHAS* (*21-nt phasiRNA-generating*) precursors in input (Total), total polysome (TP) and membrane-bound polysome (MBP) samples from maize immature tassels (A) and rice immature panicles (C). **(B, D)** Abundance of reproductive *24PHAS* (*24-nt phasiRNA-generating*) precursors in Total, TP and MBP samples from maize immature tassels (B) and rice immature panicles (D). Abundance of reproductive *PHAS* precursors is displayed as the mean of three biological repeats. “RP10M” is short for “reads per 10 million mapped reads”.

**
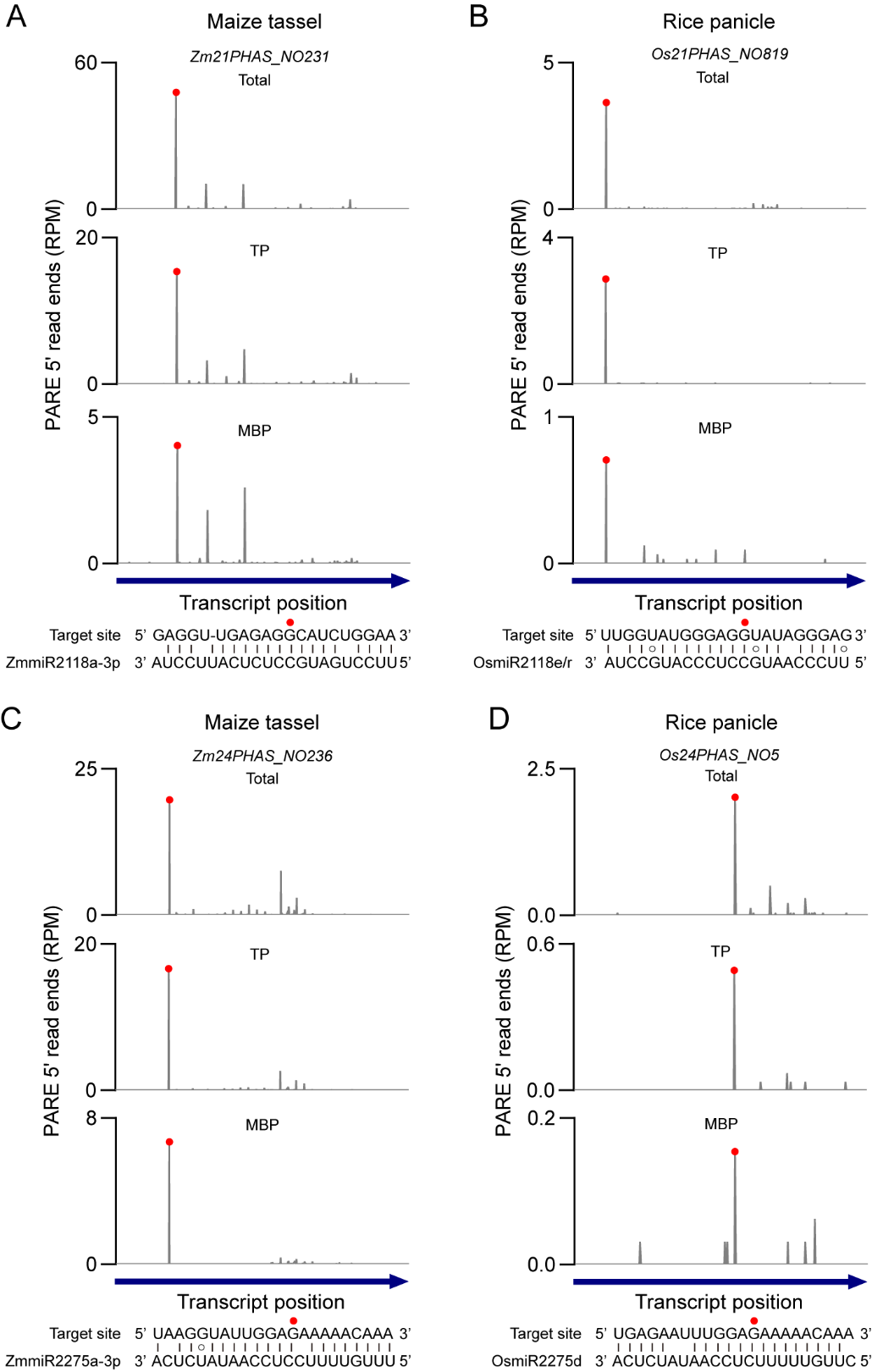
**

**Fig. S15** PARE 3’ cleavage fragment coverage for reproductive *21PHAS* and *24PHAS* precursors targeted by miR2118 and miR2275, respectively, in maize immature tassels and rice immature panicles. **(A)** PARE 3’ cleavage fragment coverage for *Zm21PHAS_NO231*, an identified *21PHAS* (*21-nt phasiRNA-generating*) precursor that was cleaved by miR2118a-3p, in input (Total), total polysome (TP) and membrane-bound polysome (MBP) samples from maize immature tassels. **(B)** PARE 3’ cleavage fragment coverage for *Os21PHAS_NO819*, an identified *21PHAS* precursor that was cleaved by miR2118e/r, in Total, TP and MBP samples from rice immature panicles. **(C)** PARE 3’ cleavage fragment coverage for *Zm24PHAS_NO236*, an identified *24PHAS* (*24-nt phasiRNA-generating*) precursor that was cleaved by miR2275a-3p, in Total, TP and MBP samples from maize immature tassels. **(D)** PARE 3’ cleavage fragment coverage for *Os24PHAS_NO5*, an identified *24PHAS* precursor that was cleaved by miR2275d, in Total, TP and MBP samples from rice immature panicles. The red dots indicate the cleavage sites on *PHAS* transcripts. The sequences of the target sites and the corresponding miRNAs are shown at the bottom. “PARE” and “RPM” are short for “parallel analysis of RNA ends” and “reads per million mapped reads”, respectively.

**
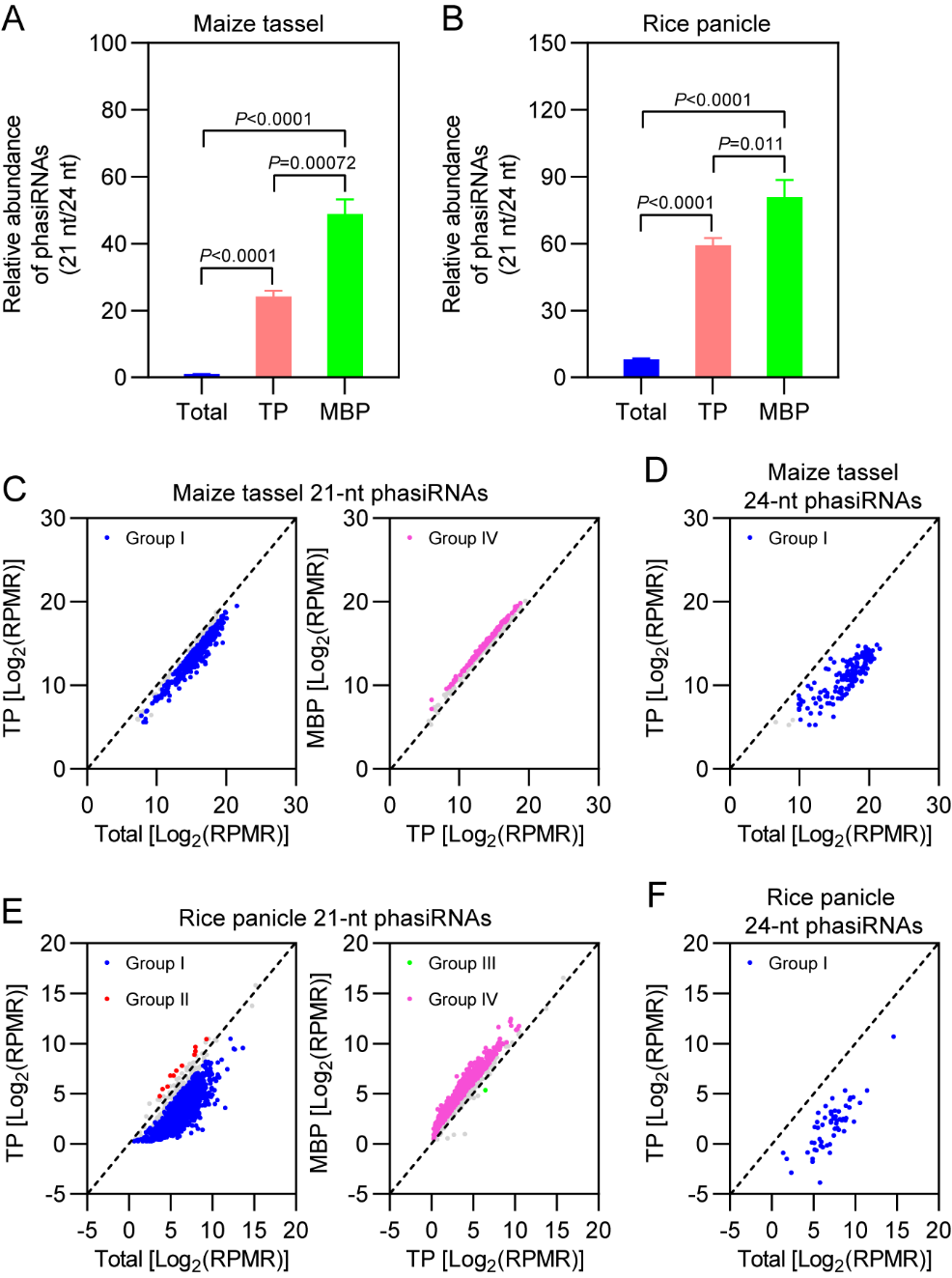
**

**Fig. S16** Distinct subcellular partitioning of reproductive 21-nt and 24-nt phasiRNAs in maize immature tassels and rice immature panicles. **(A–B)** Ratios of reproductive phasiRNA abundance between 21-nt and 24-nt classes in maize immature tassels (A) and rice immature panicles (B). Skewed distribution of 21-nt phasiRNAs towards total polysome (TP) and membrane-bound polysome (MBP) relative to input (Total) samples is observed. **(C, E)** Identification of differentially accumulated reproductive 21-nt phasiRNAs between TP and Total (left panels), and between MBP and TP (right panels) in maize immature tassels (C) and rice immature panicles (E). **(D, F)** Identification of differentially accumulated reproductive 24-nt phasiRNAs between TP and Total in maize immature tassels (D) and rice immature panicles (F). phasiRNA abundance is displayed as mean ± standard deviation (SD) (A and B) or the mean of three biological repeats (C–F). “RPMR” is short for “reads per million rRNA fragments”. The cutoff parameters for differentially accumulated phasiRNAs are fold change >= 2 and *P*-value <= 0.05. “Group I”, “Group II”, “Group III” and “Group IV” represent “phasiRNAs that were polysome-depleted”, “phasiRNAs that were polysome-enriched”, “phasiRNAs that were polysome-associated but MBP-depleted” and “phasiRNAs that were MBP-enriched”, respectively.

**
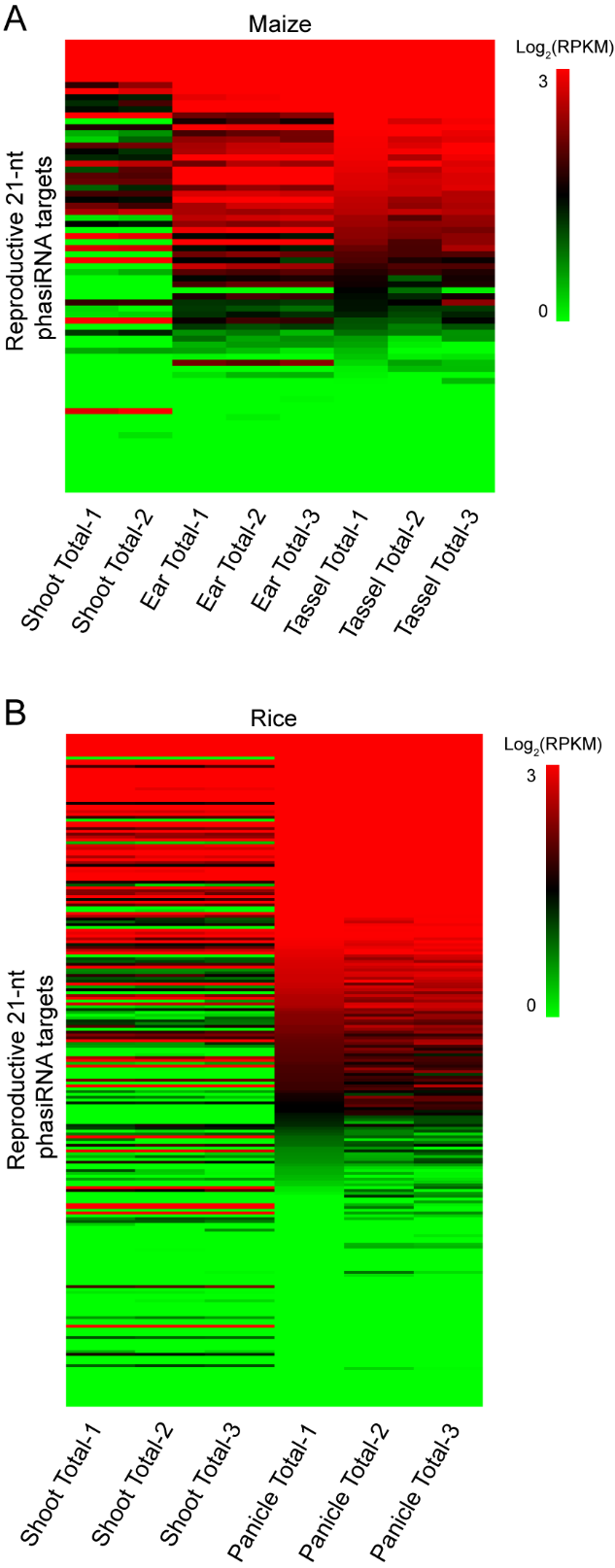
**

**Fig. S17** Expression of genes targeted by reproductive 21-nt phasiRNAs in maize and rice. **(A)** Abundance of transcripts targeted by reproductive 21-nt phasiRNAs in maize seedling shoots, immature ears and immature tassels. **(B)** Abundance of transcripts targeted by reproductive 21-nt phasiRNAs in rice seedling shoots and immature panicles. “Total-1”, “Total-2” and “Total-3” represent different biological repeats of total extracts without fractionation. “RPKM” is short for “reads per kilobase of transcript per million mapped reads”.
