# Supplementary material for: Widespread occurrence of microRNA-mediated target cleavage on membrane-bound polysomes
Source: Genome Biol. 2021 Jan 5;22:15. doi: 10.1186/s13059-020-02242-6 (PMC7784310; doi:10.1186/s13059-020-02242-6)
Supplement: Supplementary file 19 — Additional file 19. Review history. [file 13059_2020_2242_MOESM19_ESM.docx]

Review History

**First round of review**

**Reviewer 1**

**Are you able to assess all statistics in the manuscript, including the appropriateness of statistical tests used?**

No, I do not feel adequately qualified to assess the statistics.

**Comments to author:**

Yang et al. reports a study of miRNA-mediated RNA cleavage on membrane-bound polysomes (MBP). As mentioned in the introduction part, TAS and PHAS RNA cleavages by miRNA on MBP were already discovered in Arabidopsis (Li et al, 2016). This study confirms this biological phenomenon exists in two monocot species maize and rice and in different tissue types. PARE-seq data confirms well the cleavage of target RNA on MBP. TE siRNA accumulation in polysomes was more pronounced in maize and rice due to higher contents of transposable elements in these monocot species than Arabidopsis although it was not a main interest in this manuscript. The manuscript was sometimes written too lengthy especially for the results part. Here are some other questions and suggestions regarding authors' conclusions.

-	Table S1 summarizes numbers of sequenced and mapped reads for total RNA, TP and MBP sRNA samples. Sequenced/Mapped read ratios are quite consistent among three types of sRNA samples. However, 21-24 nt sRNA (e.g. Fig 1B and 1D) shows overall higher levels of sRNA in total RNA fraction than in polysome fractions. Is this because tRNA was more enriched in the TP and MBP sRNA samples? There could be other reasons such as sRNA normalization to rRNA abundance in each library and PCR cycles for library construction.

-	Any explanation about potential functions of TE siRNA enriched in MBP? Their target mRNA are also enriched in MBP? Are PARE-seq reads found at these TE mRNA? Or TE siRNA are thought to mediate translational repression.

-	Since they use multiple datasets of maize and rice plants and different tissue types. For easier interpretation, one suggestion is to label plots with plant species and tissue types throughout all the possible figures and supplementary figures.

-	Figure 1B: RPMR needs to be spelled out in Figure legend.

-	Figure 5E, 5F: It might be better to use 21nt/24nt rather than use absolute values 21 phasiRNA.

**Reviewer 2**

**Are you able to assess all statistics in the manuscript, including the appropriateness of statistical tests used?**

Yes, and I have assessed the statistics in my report.

**Comments to author:**

In this paper, Yang et al analyze miRNA targeting and phasiRNA biogenesis in different tissues of maize and rice at the subcellular level using polysome fractionations. They found that together with miRNAs, 22-nt TE-derived siRNAs and 21-nt reproductive phasiRNAs are enriched in membrane-bound polysomes (MBPs). The authors conclude that endoplasmic reticulum-bound ribosomes have a role in miRNA-induced gene silencing and reproductive phasiRNA biogenesis in these two plant species. These findings are a continuation of their work in Arabidopsis and provide confirmation and an exploration of the role of RNA silencing at the subcellular level. Nevertheless, there are some aspects of the manuscript that need to be clarified or improved. Detailed comments are listed below.

Major comments:

1. The analysis of the quality of the fractionation and their derived datasets must be improved. First, in the supplementary figure S1 A-J the profiles for the TP and MBP fractionations are provided but the enrichment in monosomes in the MBP fractions from reproductive tissues (which is not obvious) is not quantified. With the information provided it is hard to know to what extent the fractionation worked. Also, could the authors provide the analysis of any marker of the MBP or TP fractions absent in the total fraction that could be used to check that the separation worked as expected?

Second, it would be better to perform the correlation between bioreplicates including all the samples in the analysis so the clustering of all the sRNA libraries derived from the same tissues is assessed.

Finally, are the authors convinced that the normalization to rRNA reads provides an accurate overview of the sRNA profiles here? In their previous work it was useful to put all the profiles at a comparable level, but in this work the total fraction seems to have a way higher accumulation of sRNAs which makes hard to see any enrichment. How would the profiles look with standard reads-per-million normalization?

2. Although it is true that the MBP and TP fractions have somehow higher level of 21 and 22-nt sRNAs compared to the total fraction these results are strikingly different from the previous work from the same lab in similar fractions from Arabidopsis tissues. In the text it is mentioned that the skewing towards higher abundance of 21 nt sRNAs in the MBP fraction is similar to the observed in Arabidopsis. This is slightly true but, in all cases, the 21/24 and 22/24 sRNA ratios are very below 1 showing that the amount of 21 and 22-nt sRNAs is always lower than the amount of 24-nt sRNAs. I am not convinced that this data is showing any general trend towards an enrichment of 21 and 22-nt sRNAs or similarity with previous results in Arabidopsis as commented in the text.

On the similar terms, in the text it is commented that there is a skewing of TE-derived sRNAs towards the 22-nt size class in figure S5A to C, but to make this claim the skewing needs further analysis with statistical tests similar to the ones in Figure 1 C and E.

3. Categorization of sRNA increased or reduced accumulation in groups (as mentioned in the text) is very confusing. First, groups are not showed or mentioned in the figures, which are showing individual size classes. So, for a single group there could be 3 different figures (for 21, 22 and 24-nt sRNAs in the case of TEs). Could the authors find a way to show this data in a less confusing way? Maybe grouping all size classes in a single graph with different colors for each sRNA size? or at least indicating in the top of the graph what kind of group they refer to and a legend to indicate what each group means? This would tremendously help the reader. On the same terms, since the work uses tissue from 2 plant species and 5 different tissues it would be a good idea to include in the graphs the tissue that they refer to. Right now, the lack of those elements makes the manuscript hard to read and interpret.

4. Increased or reduced accumulation of sRNAs is considered if higher than 1.5-fold. This might be too low and could be prone to include several false positives. I would consider as the lowest limit at least a 2-fold increase/decrease.

5. Where PARE libraries constructed with bioreplicates? Analysis of miRNA and siRNA target sites detected by PARE is strongest if detected in multiple biological replicates. If cleavage sites were only detected in a single replicate it is prone to an overestimation of the number of cleavage sites (this is also commented in the Cleaveland manual information). Furthermore, without bioreplicates it is difficult to claim an enrichment of any kind in any fraction since no statistical test can be produced.

Also, PARE reads where not normalized to reads per million. I am afraid that PARE reads need to be normalized somehow (total mapped reads or ribosomal reads) to be able to make comparisons between the different fractions.

6. In my opinion the MBP enrichment of 22-nt TE-derived siRNAs is not analyzed in detail although it is very interesting. How do the authors justify the massive accumulation of this sRNA class enriched in MBPs? Do the authors find evidences of their activity in their PARE libraries? What are these sRNAs targeting? Do they also find transcripts from the precursors TEs in their mRNA sequencing data?

Minor comments:

1. Using the terms hypo and hyper is not preceded of any explanation. Furthermore, hyper and hypo are very commonly used in epigenetics to refer to hypermethylation and hypomethylation of regions. Since in the text they refer to TEs, could the authors use a different term to refer to increased and decreased accumulation?

2. An alternative explanation for the overlapping of 22-nt and 24-nt producing loci would be that DCL2 and DCL3 share TE transcripts that produce 22 and 24-nt sRNAs in different compartments. It would be interesting to quantify the amount of sense and antisense sRNAs for each size class to infer if they derive from Pol II-Pol IV transcription or they derive from transcripts that experimented RDR-mediated amplification.

3. In their previous publication the authors found a characteristic enrichment of miRNAs of 22 nts in length in the MBP fraction. Have this been analyzed for these datasets in maize and rice? It would be interesting to at least comment if that enrichment is not conserved in other species.

4. The enrichment of miR2118 and miR2275 members in MBPs is not obvious and, furthermore, the statistical analysis leading to that claim for individual miRNAs is not showed in the figure or the text. This claim needs to be supported by the appropriate statistical test.

**Authors Response**

**Point-by-point responses to the reviewers’ comments:**

Point-by-point responses to Reviewer 1’s comments:

Yang et al. reports a study of miRNA-mediated RNA cleavage on membrane-bound polysomes (MBP). As mentioned in the introduction part, TAS and PHAS RNA cleavages by miRNA on MBP were already discovered in Arabidopsis (Li et al, 2016). This study confirms this biological phenomenon exists in two monocot species maize and rice and in different tissue types. PARE-seq data confirms well the cleavage of target RNA on MBP. TE siRNA accumulation in polysomes was more pronounced in maize and rice due to higher contents of transposable elements in these monocot species than Arabidopsis although it was not a main interest in this manuscript.

(1) The manuscript was sometimes written too lengthy especially for the results part.

*Response: The manuscript was shortened, especially the Results section, in the revised manuscript.*

Here are some other questions and suggestions regarding authors' conclusions.

(2) Table S1 summarizes numbers of sequenced and mapped reads for total RNA, TP and MBP sRNA samples. Sequenced/Mapped read ratios are quite consistent among three types of sRNA samples. However, 21-24 nt sRNA (e.g. Fig 1B and 1D) shows overall higher levels of sRNA in total RNA fraction than in polysome fractions. Is this because tRNA was more enriched in the TP and MBP sRNA samples? There could be other reasons such as sRNA normalization to rRNA abundance in each library and PCR cycles for library construction.

*Response: In this study, we normalized sRNA reads against rRNAs instead of total reads according to the previous study (Li et al., 2016). The main reason for the higher abundance of normalized sRNAs in Total than in TP and MBP samples is the difference in rRNA abundance between Total and polysome samples. Higher abundance of rRNA-derived sRNAs was observed in TP and MBP sRNA libraries than Total sRNA libraries. We used the same number of PCR cycles (12 cycles) for sRNA library construction, thus the higher abundance of normalized sRNAs in Total than TP and MBP samples cannot be explained by the differences in PCR amplification during library preparation.*

*In fact, sRNA reads were normalized both against rRNAs and total mapped reads, a more commonly used method, when we analyzed our data. The results and conclusions, such as abundance and size distribution of sRNAs (sRNAs in general, TE-siRNAs, miRNAs and phasiRNAs) and their subcellular enrichment, were largely similar between these two normalization methods – compare Response Figures 1-5 in our response letter, which were derived from normalization against total mapped reads, to figures in the manuscript derived from normalization against rRNAs. One exception is that a higher overall abundance of sRNAs (sRNAs in general, TE-siRNAs, miRNAs and phasiRNAs) by normalization against total mapped reads was observed in MBP samples than that in both Total and TP samples in some maize and rice tissues (Response Figures 1-5 in our response letter). However, we found that the levels of miRNAs as determined by normalization against total mapped reads, such as miR390a/b-5p and miR529-5p in maize immature tassels and miR390-5p in rice immature panicles, were not consistent with Northern blotting results (Response Figure 6 in our response letter). The total read-normalized abundance of these miRNAs was exaggerated in polysome samples, especially in MBP samples. This problem was also observed in our previous study (Li et al., 2016). In contrast, we found that miRNA abundance as determined by normalization against rRNAs was consistent with Northern blotting results (Response Figure 6 in our response letter). rRNA-based normalization was thus adopted in this study.*

*Response Figure 1. Distinct subcellular partitioning of 21-nt/22-nt and 24-nt small RNAs in different tissues of maize and rice. (A, C, E, G, I) Size distribution of small RNAs (sRNAs) in input (Total), total polysome (TP) and membrane-bound polysome (MBP) samples from maize seedling shoots (A), immature ears (C), immature tassels (E), rice seedling shoots (G), and immature panicles (I). (B, D, F, H, J) Ratios of sRNA abundance between 21-nt and 24-nt and between 22-nt and 24-nt classes in maize seedling shoots (B), immature ears (D), immature tassels (F), rice seedling shoots (H), and immature panicles (J). Skewed distribution of 21-nt and 22-nt sRNAs towards TP and MBP (B, F, H and J) and towards MBP (D) relative to Total samples is observed. sRNA abundance is displayed as mean ± standard deviation (SD) of two biological repeats for maize seedling shoots (A and B), and of three biological repeats for maize immature ears (C and D), immature tassels (E and F), rice seedling shoots (G and H), and immature panicles (I and J). “RPM” is short for “reads per million mapped reads” (A, C, E, G and I). Comparisons between Total, TP and MBP were performed by two-tailed unpaired t-test, and P-values are displayed above the corresponding comparisons (B, D, F, H and J).*

*Response Figure 2. Distinct subcellular partitioning of 22-nt and 24-nt transposable element-derived siRNAs in different tissues of maize and rice. (A, C, E, G, I) Size distribution of transposable element (TE)-derived siRNAs (TE-siRNAs) in input (Total), total polysome (TP) and membrane-bound polysome (MBP) samples from maize seedling shoots (A), immature ears (C), immature tassels (E), rice seedling shoots (G), and immature panicles (I). (B, D, F, H, J) Ratios of TE-siRNA abundance between 22-nt and 24-nt classes in maize seedling shoots (B), immature ears (D), immature tassels (F), rice seedling shoots (H), and immature panicles (J). Skewed distribution of 22-nt TE-siRNAs towards TP and MBP (B, F, H and J) and towards MBP (D) relative to Total samples is observed. TE-siRNA abundance is displayed as mean ± standard deviation (SD) of two biological repeats for maize seedling shoots (A and B), and of three biological repeats for maize immature ears (C and D), immature tassels (E and F), rice seedling shoots (G and H), and immature panicles (I and J). “RPM” is short for “reads per million mapped reads” (A, C, E, G and I). Comparisons between Total, TP and MBP were performed by two-tailed unpaired t-test, and P-values are displayed above the corresponding comparisons (B, D, F, H and J).*

*Response Figure 3. Identification of differentially accumulated 22-nt and 24-nt transposable element-derived siRNAs in different maize tissues. (A, C, E) Identification of differentially accumulated 22-nt transposable element (TE)-derived siRNAs (TE-siRNAs) between total polysome (TP) and input (Total) (left panel), and between membrane-bound polysome (MBP) and TP (right panel) in maize seedling shoots (A), immature ears (C), and immature tassels (E). (B, D, F) Identification of differentially accumulated 24-nt TE-siRNAs between TP and Total (left panel), and between MBP and TP (right panel) in maize seedling shoots (B), immature ears (D), and immature tassels (F). TE-siRNA abundance is displayed as the mean of two biological repeats for maize seedling shoots (A and B), and of three biological repeats for maize immature ears (C and D) and immature tassels (E and F). “RPM” is short for “reads per million mapped reads”. The cutoff parameters for differentially accumulated TE-siRNAs are fold change >= 2 and P-value <= 0.05. “Group I”, “Group II”, “Group III” and “Group IV” represent “TE loci with siRNAs that were polysome-depleted”, “TE loci producing siRNAs that were polysome-associated”, “TE loci with siRNAs that were likely on free polysomes (FPs)” and “TE loci with siRNAs enriched on MBPs”, respectively.*

*Response Figure 4. Overaccumulation of miRNAs on membrane-bound polysomes in different tissues of maize and rice. (A, C, E, G, I) Size and abundance of all miRNAs in maize seedling shoots (A), immature ears (C), immature tassels (E), rice seedling shoots (G), and immature panicles (I). (B, D, F, H, J) Identification of differentially accumulated miRNAs between total polysome (TP) and input (Total) (left panel), and between membrane-bound polysome (MBP) and TP (right panel) in maize seedling shoots (B), immature ears (D), immature tassels (F), rice seedling shoots (H), and immature panicles (J). miRNA abundance is displayed as mean ± standard deviation (SD) (A, C, E, G and I) or the mean of two biological repeats for maize seedling shoots (B), and of three biological repeats for maize immature ears (D), immature tassels (F), rice seedling shoots (H), and immature panicles (J). “nt” stands for “nucleotide” (A, C, E, G and I), and “RPM” is short for “reads per million mapped reads”. The cutoff parameters for differentially accumulated miRNAs are fold change >= 2 and P-value <= 0.05. “Group I”, “Group II”, “Group III” and “Group IV” represent “miRNAs that were polysome-depleted”, “miRNAs overaccumulated on polysomes”, “miRNAs that were associated with polysomes but MBP-depleted” and “miRNAs that were MBP-enriched”, respectively (B, D, F, H and J).*

*Response Figure 5. Distinct subcellular partitioning of reproductive 21-nt and 24-nt phasiRNAs in maize immature tassels and rice immature panicles. (A, C) Abundance of reproductive 21-nt phasiRNAs in input (Total), total polysome (TP) and membrane-bound polysome (MBP) samples from maize immature tassels (A) and rice immature panicles (C). (B, D) Identification of differentially accumulated reproductive 21-nt phasiRNAs between TP and Total (left panel), and between MBP and TP (right panel) in maize immature tassels (B) and rice immature panicles (D). (E, G) Abundance of reproductive 24-nt phasiRNAs in Total, TP and MBP samples from maize immature tassels (E) and rice immature panicles (G). (F, H) Identification of differentially accumulated reproductive 24-nt phasiRNAs between TP and Total in maize immature tassels (F) and rice immature panicles (H). phasiRNA abundance is displayed as mean ± standard deviation (SD) (A, C, E and G) or the mean of three biological repeats (B, D, F and H). “RPM” is short for “reads per million mapped reads”. The cutoff parameters for differentially accumulated phasiRNAs are fold change >= 2 and P-value <= 0.05. “Group I”, “Group II”, “Group III” and “Group IV” represent “phasiRNAs that were polysome-depleted”, “phasiRNAs that were polysome-enriched”, “phasiRNAs that were polysome-associated but MBP-depleted” and “phasiRNAs that were MBP-enriched”, respectively (B, D, F and H).*

*Response Figure 6. Northern blotting for select miRNAs detected in sRNA libraries from different subcellular compartments of maize and rice. (A) Abundance of miR390a/b-5p from total read-normalized sRNA datasets (left panel), rRNA-normalized sRNA datasets (middle panel) and Northern blotting (right panel) in input (Total), total polysome (TP) and membrane-bound polysome (MBP) samples from maize immature tassels. (B) Abundance of miR529-5p from total read-normalized sRNA datasets (left panel), rRNA-normalized sRNA datasets (middle panel) and Northern blotting (right panel) in Total, TP and MBP samples from maize immature tassels. (C) Abundance of miR390-5p from total read-normalized sRNA datasets (left panel), rRNA-normalized sRNA datasets (middle panel) and Northern blotting (right panel) in Total, TP and MBP samples from rice immature panicles. In left and middle panels of (A-C), miRNA abundance is displayed as mean ± standard deviation (SD) of three biological repeats, and “RPM” and “RPMR” are short for “reads per million mapped reads” and “reads per million rRNA fragments” respectively. In the right panels of (A-C), 5S rRNA was used as an internal control, and U6 was used as a nuclear RNA marker.*

(3) Any explanation about potential functions of TE siRNA enriched in MBP? Their target mRNA are also enriched in MBP? Are PARE-seq reads found at these TE mRNA? Or TE siRNA are thought to mediate translational repression.

*Response: Based on your suggestion, we extracted the sequences of the 500 most abundant 22-nt MBP-associated TE-siRNAs, and then used them as queries to search for potential targets in the corresponding PARE libraries. By following the criteria of category = 0 and P-value <= 0.05 in at least two biological repeats, some target transcripts from annotated genes and TE loci were identified in MBP samples from different maize tissues, which partially support the functionality of MBP-enriched 22-nt TE-siRNAs. These results were added in the revised manuscript as Table S5 and Table S6.*

*To answer whether target cleavage by 22-nt TE-siRNAs on MBPs was associated with the enrichment of target transcripts on MBPs, we analyzed the expression levels of target genes in the RNA-seq libraries of Total, TP and MBP samples from maize immature tassels (Response Figure 7 in our response letter). No expression bias was observed between Total, TP and MBP, indicating that target cleavage by 22-nt TE-siRNAs on MBPs is not due to the enrichment of target transcripts on MBPs. These results were consistent with our findings about miRNA and 21-nt phasiRNA target genes.*

*Response Figure 7. Transcript levels of genes targeted by 22-nt TE-siRNAs on membrane-bound polysomes (MBPs) in input (Total), total polysome (TP) and MBP samples from maize immature tassels. “Total-1,2,3”, “TP-1,2,3” and “MBP-1,2,3” represent different biological repeats. “RPM” is short for “reads per million mapped reads”.*

(4) Since they use multiple datasets of maize and rice plants and different tissue types. For easier interpretation, one suggestion is to label plots with plant species and tissue types throughout all the possible figures and supplementary figures.

*Response: Labels for species and tissues were added in the figures.*

(5) Figure 1B: RPMR needs to be spelled out in Figure legend.

*Response: This was done.*

(6) Figure 5E, 5F: It might be better to use 21nt/24nt rather than use absolute values 21 phasiRNA.

*Response: The relative abundance (21nt/24nt) of phasiRNAs was provided in the manuscript in Figure S16. By using this parameter, the distinct partitioning of 21-nt and 24-nt phasiRNAs between cytosol and polysome was better reflected. However, we kept the absolute values of phasiRNAs in Figures 5 and 6 in the revised manuscript as well, because the observation that 24-nt phasiRNAs were nearly completely depleted from polysome samples could not be well represented if we only used relative abundance.*

Point-by-point responses to Reviewer 2’s comments:

In this paper, Yang et al analyze miRNA targeting and phasiRNA biogenesis in different tissues of maize and rice at the subcellular level using polysome fractionations. They found that together with miRNAs, 22-nt TE-derived siRNAs and 21-nt reproductive phasiRNAs are enriched in membrane-bound polysomes (MBPs). The authors conclude that endoplasmic reticulum-bound ribosomes have a role in miRNA-induced gene silencing and reproductive phasiRNA biogenesis in these two plant species. These findings are a continuation of their work in Arabidopsis and provide confirmation and an exploration of the role of RNA silencing at the subcellular level. Nevertheless, there are some aspects of the manuscript that need to be clarified or improved. Detailed comments are listed below.

Major comments:

The analysis of the quality of the fractionation and their derived datasets must be improved.

(1) First, in the supplementary figure S1 A-J the profiles for the TP and MBP fractionations are provided but the enrichment in monosomes in the MBP fractions from reproductive tissues (which is not obvious) is not quantified. With the information provided it is hard to know to what extent the fractionation worked. Also, could the authors provide the analysis of any marker of the MBP or TP fractions absent in the total fraction that could be used to check that the separation worked as expected?

*Response: We deleted the description about the differences in TP and MBP profiles between vegetative and reproductive tissues, because, as you pointed out, these differences were not obvious and contributed little to the main findings in our study. To further support the effective separation of polysomal fractions, we performed both Western and Northern blotting analyses for TP and MBP samples from maize seedling shoots, immature ears, immature tassels, rice seedling shoots and immature panicles (Response Figure 8 in our response letter). For Western blotting analysis, we used ribosomal protein L13 (RPL13), a component of the 60S ribosome subunit, and phosphoenolpyruvate carboxylase (PEPC), a cytosolic protein depleted from polysomal fractions, as markers to evaluate the purity of isolated TP and MBP samples. We observed that signals for RPL13 were enriched, while signals for PEPC were nearly completely depleted, in TP and MBP samples in comparison to those in Total samples, indicating that polysomal fractionation was successful. Consistent with the Western blotting results, the Northern blotting signals of U6 small nuclear RNA were almost depleted in TP and MBP samples, further supporting the successful isolation of polysomal fractions. These blotting results were added in the revised manuscript as Figure S2.*

*Response Figure 8. Effective isolation of total polysomes and membrane-bound polysomes from different tissues of maize and rice. (A-E) Detection of ribosomal protein L13 (RPL13) and phosphoenolpyruvate carboxylase (PEPC) by Western blotting in input (Total), total polysome (TP) and membrane-bound polysome (MBP) samples from maize seedling shoots (A), immature ears (B), immature tassels (C), rice seedling shoots (D), and immature panicles (E). RPL13 is a component of the 60S ribosome subunit and expected to be in the polysomal fractions, while PEPC is a cytosolic protein and expected to be depleted from the polysomal fractions. Images of protein staining by Coomassie Brilliant Blue are shown on the left to indicate equal loading. “M”: molecular weight standards. (F-J) Detection of 5S rRNA and U6 by Northern blotting in Total, TP and MBP samples from maize seedling shoots (F), immature ears (G), immature tassels (H), rice seedling shoots (I), and immature panicles (J). 5S rRNA is expected to be in the polysome and input samples, while U6 is a small nuclear RNA and expected to be depleted from polysomal fractions. Ethidium bromide (EtBr)-stained rRNA was used as a loading control.*

(2) Second, it would be better to perform the correlation between bioreplicates including all the samples in the analysis so the clustering of all the sRNA libraries derived from the same tissues is assessed.

*Response: Thank you for this suggestion. The recommended clustering analyses were performed for sRNA datasets of maize and rice (Response Figure 9 in our response letter) and the results were included in the revised manuscript as Figure S3.*

*Response Figure 9. Clustering analysis for small RNA-seq datasets from input, total polysome and membrane-bound polysome samples from different tissues of maize and rice. (A) Clustering analysis for small RNA (sRNA)-seq datasets from input (Total), total polysome (TP) and membrane-bound polysome (MBP) samples from maize seedling shoots, immature ears and immature tassels. (B) Clustering analysis for sRNA-seq datasets from Total, TP and MBP samples from rice seedling shoots and immature panicles. “Total-1, 2, 3”, “TP-1, 2, 3” and “MBP-1, 2, 3” represent different biological repeats. The scale bars indicate Euclidean distances between samples measured by DESeq2 normalized read counts.*

(3) Finally, are the authors convinced that the normalization to rRNA reads provides an accurate overview of the sRNA profiles here? In their previous work it was useful to put all the profiles at a comparable level, but in this work the total fraction seems to have a way higher accumulation of sRNAs which makes hard to see any enrichment. How would the profiles look with standard reads-per-million normalization?

*Response: Reviewer 1 also asked this question. Please see our responses above. Results with the standard reads-per-million normalization are shown in Response Figures 1-5, and issues with this normalization are shown in Response Figure 6.*

(4) Although it is true that the MBP and TP fractions have somehow higher level of 21 and 22-nt sRNAs compared to the total fraction these results are strikingly different from the previous work from the same lab in similar fractions from Arabidopsis tissues. In the text it is mentioned that the skewing towards higher abundance of 21 nt sRNAs in the MBP fraction is similar to the observed in Arabidopsis. This is slightly true but, in all cases, the 21/24 and 22/24 sRNA ratios are very below 1 showing that the amount of 21 and 22-nt sRNAs is always lower than the amount of 24-nt sRNAs. I am not convinced that this data is showing any general trend towards an enrichment of 21 and 22-nt sRNAs or similarity with previous results in Arabidopsis as commented in the text.

*Response: In the previous study on Arabidopsis seedling small RNAs, we did not compare miRNA abundance between TP and Total samples. Here, we analyzed the published Arabidopsis datasets and found that, in addition to the previously reported MBP vs. TP enrichment, miRNAs were also enriched in Total vs. TP (Response Figure 10 in our response letter), indicating that both cytosol and MBP are the major pools for miRNAs in Arabidopsis. These observations in Arabidopsis seedlings are largely consistent with our findings in maize and rice in this study. We also noted that the overall abundance of sRNAs in MBP samples from Arabidopsis seedlings was higher than that in Total and TP samples, while in most cases of maize and rice the overall abundance of sRNAs in MBP samples was higher than TP samples but lower than Total samples. The possible reasons for this inconsistency were discussed in the manuscript. First, the composition of 21-nt/22-nt sRNAs were quite different between the two monocots and Arabidopsis. For the two monocots, siRNAs including TE-siRNAs, I-siRNAs, G-siRNAs and P-siRNAs accounted for the overwhelming majority of 21-nt/22-nt siRNAs, while miRNAs only occupied a very small proportion. On the contrary, the major component of 21-nt/22-nt sRNAs, particularly 21-nt sRNAs, were miRNAs, while siRNAs accounted for a minor proportion in Arabidopsis seedlings. Therefore, the abundance of 21-nt/22-nt sRNAs was largely determined by miRNAs in Arabidopsis, while it was largely determined by siRNAs in maize and rice. As miRNAs are more enriched in the MBP fractions as compared to siRNAs, it is reasonable that the enrichment of 21-nt/22-nt sRNAs in the MPB samples is more striking in Arabidopsis. Consistent with the observation in Arabidopsis, we also found that polysome-associated miRNAs were enriched on MBPs in the different tissues of maize and rice. A second possible explanation is the tissue type. The Arabidopsis paper only examined seedlings. We cannot exclude the possibility that the enrichment of sRNAs exhibits tissue-to-tissue variations. In fact, miRNA and 21-nt sRNA abundance in rice seedling shoots exhibited the highest level in MBP samples, the same distribution pattern as in Arabidopsis seedlings.*

*To make the description about the MBP enrichment of miRNAs and some 21-nt/22-nt siRNAs more precise, in the Discussion section, we emphasized that this enrichment was relative to other sRNAs such as 24-nt siRNAs or relative to polysomes in general, and many miRNAs and 21-nt/22-nt siRNAs also accumulated in polysome-depleted fractions, probably the cytosol.*

*Response Figure 10. Distinct subcellular partitioning of miRNAs in Arabidopsis seedlings (Li et al., 2016). Identification of differentially accumulated miRNAs between total polysome (TP) and input (Total) (left panel), and between membrane-bound polysome (MBP) and TP (right panel) in Arabidopsis seedlings. miRNA abundance is displayed as the mean of three biological repeats, and “RPMR” is short for “reads per million rRNA fragments”. The cutoff parameters for differentially accumulated miRNAs are fold change >= 2 and P-value <= 0.05. “Group I”, “Group II” and “Group IV” represent “miRNAs that were polysome-depleted”, “miRNAs overaccumulated on polysomes” and “miRNAs that were MBP-enriched”, respectively.*

(5) On the similar terms, in the text it is commented that there is a skewing of TE-derived sRNAs towards the 22-nt size class in figure S5A to C, but to make this claim the skewing needs further analysis with statistical tests similar to the ones in Figure 1 C and E.

*Response: The recommended analysis was done for TE-siRNAs and the results were included in Figure S6 in the revised manuscript.*

(6) Categorization of sRNA increased or reduced accumulation in groups (as mentioned in the text) is very confusing. First, groups are not showed or mentioned in the figures, which are showing individual size classes. So, for a single group there could be 3 different figures (for 21, 22 and 24-nt sRNAs in the case of TEs). Could the authors find a way to show this data in a less confusing way? Maybe grouping all size classes in a single graph with different colors for each sRNA size? or at least indicating in the top of the graph what kind of group they refer to and a legend to indicate what each group means? This would tremendously help the reader. On the same terms, since the work uses tissue from 2 plant species and 5 different tissues it would be a good idea to include in the graphs the tissue that they refer to. Right now, the lack of those elements makes the manuscript hard to read and interpret.

*Response: Labels to indicate the different enrichment groups and the different maize and rice tissues were added in the figures, and information about enrichment groups was also provided in the legends in the revised manuscript.*

(7) Increased or reduced accumulation of sRNAs is considered if higher than 1.5-fold. This might be too low and could be prone to include several false positives. I would consider as the lowest limit at least a 2-fold increase/decrease.

*Response: Thank you for this suggestion. Enrichment analyses for sRNAs (sRNAs in general, TE-siRNAs, miRNAs and phasiRNAs) in different comparisons were done based on cutoff parameters of fold change >=2, instead of >=1.5, and P-value <= 0.05, and results from the new analyses were included in the revised manuscript.*

(8) Where PARE libraries constructed with bioreplicates? Analysis of miRNA and siRNA target sites detected by PARE is strongest if detected in multiple biological replicates. If cleavage sites were only detected in a single replicate it is prone to an overestimation of the number of cleavage sites (this is also commented in the Cleaveland manual information). Furthermore, without bioreplicates it is difficult to claim an enrichment of any kind in any fraction since no statistical test can be produced.

*Response: We performed PARE libraries with two biological repeats for maize seedling shoots, and three biological repeats for maize immature ears, immature tassels, rice seedling shoots and immature panicles. The data we provided in the original manuscript were the summary of target genes that were identified in each biological repeat by following the criteria of category = 0 and P-value <= 0.05. Based on your suggestions, we selected the target genes from PARE results by following the new criteria: category = 0, P-value <= 0.05, and being detected in at least two biological repeats. The statistical analyses for the number of targets genes were then carried out between Total, TP and MBP of different maize and rice tissues to support the claimed enrichment, and the results were included in the figures. Furthermore, information about P-values and PARE 5’ read end depth for target transcripts in each biological repeat was included in Tables S6, S9, S12 and S15.*

(9) Also, PARE reads where not normalized to reads per million. I am afraid that PARE reads need to be normalized somehow (total mapped reads or ribosomal reads) to be able to make comparisons between the different fractions.

*Response: Thank you for this suggestion. PARE reads were normalized against total mapped reads, and the data were included in the relevant figures and tables in the revised manuscript.*

(10) In my opinion the MBP enrichment of 22-nt TE-derived siRNAs is not analyzed in detail although it is very interesting. How do the authors justify the massive accumulation of this sRNA class enriched in MBPs? Do the authors find evidences of their activity in their PARE libraries? What are these sRNAs targeting? Do they also find transcripts from the precursors TEs in their mRNA sequencing data?

*Response: Based on your suggestion, we extracted the sequences of the 500 most abundant 22-nt MBP-associated TE-siRNAs, and then used them as queries to search for potential targets in the corresponding PARE libraries. By following the criteria of category = 0 and P-value <= 0.05 in at least two biological repeats, some target transcripts from annotated genes and TE loci were identified in MBP samples from different maize tissues, which partially support the functionality of MBP-enriched 22-nt TE-siRNAs. These results were added in the revised manuscript as Table S5 and Table S6.*

*To provide insights into the expression patterns of TE precursors in different subcellular compartments, we further analyzed the abundance of TE transcripts in the RNA-seq libraries of Total, TP and MBP samples from maize immature tassels. We mapped adaptor-trimmed reads from RNA-seq datasets to the AGPv4 genome for “B73” maize (https://www.maizegdb.org/) by using Bowtie2, and reads mapped to multiple positions were discarded. Then the number of remaining reads that were mapped to each annotated TE locus was counted and used for comparison of normalized TE expression with the R package DESeq2. The results showed that the expression of TE precursors was detected in not only Total samples but also TP and MBP samples, and no obvious expression bias was observed (Response Figure 11 in our response letter).*

*Response Figure 11. Levels of transposable element (TE) transcripts in input (Total), total polysome (TP) and membrane-bound polysome (MBP) samples from maize immature tassels. “Total-1,2,3”, “TP-1,2,3” and “MBP-1,2,3” represent different biological repeats. “RPM” is short for “reads per million mapped reads”.*

Minor comments:

(1) Using the terms hypo and hyper is not preceded of any explanation. Furthermore, hyper and hypo are very commonly used in epigenetics to refer to hypermethylation and hypomethylation of regions. Since in the text they refer to TEs, could the authors use a different term to refer to increased and decreased accumulation?

*Response: We used “Group I” “Group II”, “Group III” and “Group IV” to describe enriched sRNAs (sRNAs in general, TE-siRNAs, miRNAs and phasiRNAs) in different subcellular compartments instead of “Hypo in TP vs. Total”, “Hyper in TP vs. Total”, “Hypo in MBP vs. TP” and “Hyper in MBP vs. TP”, respectively, in the revised manuscript.*

(2) An alternative explanation for the overlapping of 22-nt and 24-nt producing loci would be that DCL2 and DCL3 share TE transcripts that produce 22 and 24-nt sRNAs in different compartments. It would be interesting to quantify the amount of sense and antisense sRNAs for each size class to infer if they derive from Pol II-Pol IV transcription or they derive from transcripts that experimented RDR-mediated amplification.

*Response: Based your suggestions, we calculated the ratios of sense to antisense 22-nt or 24-nt TE-siRNA abundance in different subcellular compartments of maize seedling shoots, immature ears and immature tassels. Significant differences in the ratios were observed between Total, TP and MBP samples from different maize tissues (Response Figure 12 in our response letter). For maize seedling shoots, we observed that the ratios of sense to antisense 22-nt TE-siRNAs were 0.66, 0.32 and 0.27 in Total, TP and MBP samples, respectively, and the ratios of sense to antisense 24-nt TE-siRNAs were 0.83, 0.47 and 0.35 in Total, TP and MBP samples, respectively. For maize immature ears, the ratios of sense to antisense 22-nt TE-siRNAs were 1.0, 0.69 and 0.91 in Total, TP and MBP samples, respectively, and the ratios of sense to antisense 24-nt TE-siRNAs were 0.97, 0.86 and 0.91 in Total, TP and MBP samples. For maize immature tassels, we found that the ratios of sense to antisense 22-nt TE-siRNAs were 0.81, 0.57 and 0.65 in Total, TP and MBP samples, respectively, and the ratios of sense to antisense 24-nt TE-siRNAs were 0.90, 0.77 and 0.66 in Total, TP and MBP samples, respectively. Thus, lower sense/antisense values were observed for both 22-nt and 24-nt TE-siRNAs in polysomal fractions from different maize tissues. This suggests that a portion of the sRNAs observed in the Total samples, and to a smaller extent in the TP and MBP samples, was probably degradation products of TE precursor transcripts. The lower abundance of degradation products in polysomal fractions suggests that TE transcript degradation is less efficient on polysomes.*

*In this study, we observed that the 22-nt TE-siRNAs, if polysome-associated, were on MBPs, suggesting that ER might be a novel regulatory layer for TE-siRNA-mediated PTGS (post-transcriptional gene silencing). To further support this claiming, we performed enrichment analysis for 22-nt antisense TE-siRNAs, which are generated from RDR6-mediated siRNA biosynthesis instead of degradation, in different maize tissues. Being consistent with our observations in the manuscript, Group IV (1,399 loci for maize seedling shoots, 5,333 loci for immature ears and 2,623 loci for immature tassels) also occupied the second largest proportion among the four enrichment groups (Response Figure 13 in our response letter), demonstrating the MBP enrichment of polysome-associated 22-nt antisense TE-siRNAs. While these results are very interesting, they were not included in the revised manuscript because these findings alone could not provide an explanation for the overlap of 22-nt and 24-nt TE-siRNA-generating loci.*

*Response Figure 12. Ratios of sense to antisense TE-siRNAs in different subcellular compartments of maize. (A, C, E) Ratios of sense to antisense 22-nt transposable element (TE)-derived siRNA (TE-siRNAs) in input (Total), total polysome (TP) and membrane-bound polysome (MBP) samples from maize seedling shoots (A), immature ears (C), and immature tassels (E). (B, D, F) Ratios of sense to antisense 24-nt TE-siRNAs in Total, TP and MBP samples from maize seedling shoots (B), immature ears (D), and immature tassels (F). Ratios of sense to antisense TE-siRNAs is displayed as mean ± standard deviation (SD) of two biological repeats for maize seedling shoots (A and B), and of three biological repeats for maize immature ears (C and D) and immature tassels (E and F). Comparisons between Total, TP and MBP were performed by two-tailed unpaired t-test, and P-values are displayed above the corresponding comparisons.*

*Response Figure 13. Distinct subcellular partitioning of 22-nt antisense TE-siRNAs in different tissues of maize. (A) Identification of differentially accumulated 22-nt antisense transposable element (TE)-derived siRNAs (TE-siRNAs) between total polysome (TP) and input (Total) (left panel), and between membrane-bound polysome (MBP) and TP (right panel) in maize seedling shoots. (B) Identification of differentially accumulated 22-nt antisense TE-siRNAs between TP and Total (left panel), and between MBP and TP (right panel) in maize immature ears. (C) Identification of differentially accumulated 22-nt antisense TE-siRNAs between TP and Total (left panel), and between MBP and TP (right panel) in maize immature tassels. TE-siRNA abundance is displayed as the mean of two biological repeats for maize seedling shoots (A), and of three biological repeats for maize immature ears (B) and immature tassels (C). “RPMR” is short for “reads per million rRNA fragments”. The cutoff parameters for differentially accumulated TE-siRNAs are fold change >= 2 and P-value <= 0.05. “Group I”, “Group II”, “Group III” and “Group IV” represent “TE-siRNAs that were polysome-depleted”, “TE-siRNAs enriched on polysomes”, “TE-siRNAs that were associated with polysomes but MBP-depleted and “TE-siRNAs that were MBP-enriched”, respectively.*

(3) In their previous publication the authors found a characteristic enrichment of miRNAs of 22 nt in length in the MBP fraction. Have this been analyzed for these datasets in maize and rice? It would be interesting to at least comment if that enrichment is not conserved in other species.

*Response: The subcellular partitioning of 22-nt miRNAs was characterized in different tissues of maize and rice. Consistent with the observations in Arabidopsis seedlings, these 22-nt miRNAs were enriched both in the cytosol and on MBPs, though occasionally overall abundance of miRNAs in MBP samples was not higher than that in TP samples (Response Figure 14 in our response letter). These results suggest that MBP enrichment of 22-nt miRNAs relative to polysomes in general is conserved in plants.*

(4) The enrichment of miR2118 and miR2275 members in MBPs is not obvious and, furthermore, the statistical analysis leading to that claim for individual miRNAs is not showed in the figure or the text. This claim needs to be supported by the appropriate statistical test.

*Response: The comparison and statistical analysis for the abundance of miR2118 and miR2275 members were carried out between MBP and TP in maize immature tassels and rice immature panicles, and P-values were provided in the relevant figures in the revised manuscript.*

*Reference*

*Li S, Le B, Ma X, Li S, You C, Yu Y, et al. Biogenesis of phased siRNAs on membrane-bound polysomes in Arabidopsis. eLife. 2016;5:e22750*

**Second round of review**

**Reviewer 2**

The authors have now addressed all my comments.
